# Supplementary material for: Ancestry and demography and descendants of Iron Age nomads of the Eurasian Steppe
Source: Nat Commun. 2017 Mar 3;8:14615. doi: 10.1038/ncomms14615 (PMC5337992; doi:10.1038/ncomms14615)
Supplement: Supplementary Information — Supplementary Figures 1-14, Supplementary Tables 1-28, Supplementary Note 1 and Supplementary References [file ncomms14615-s1.pdf]

Supplementary Figures

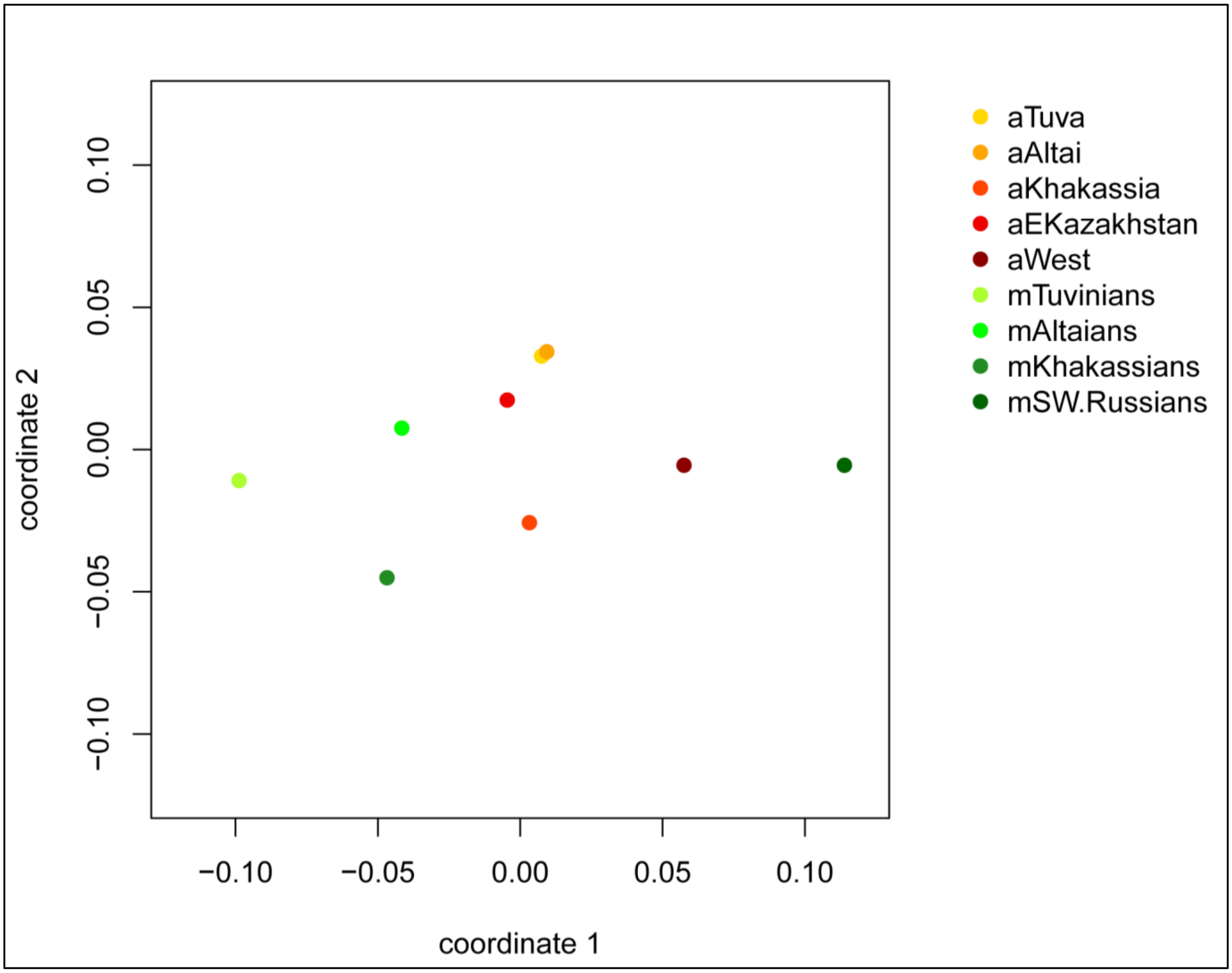

Supplementary Fig. 1. MDS plot of Iron Age populations (aX) associated with the Scythian culture and modern populations (mX) of the same geographical area[84].
Stress-value: 0.0389

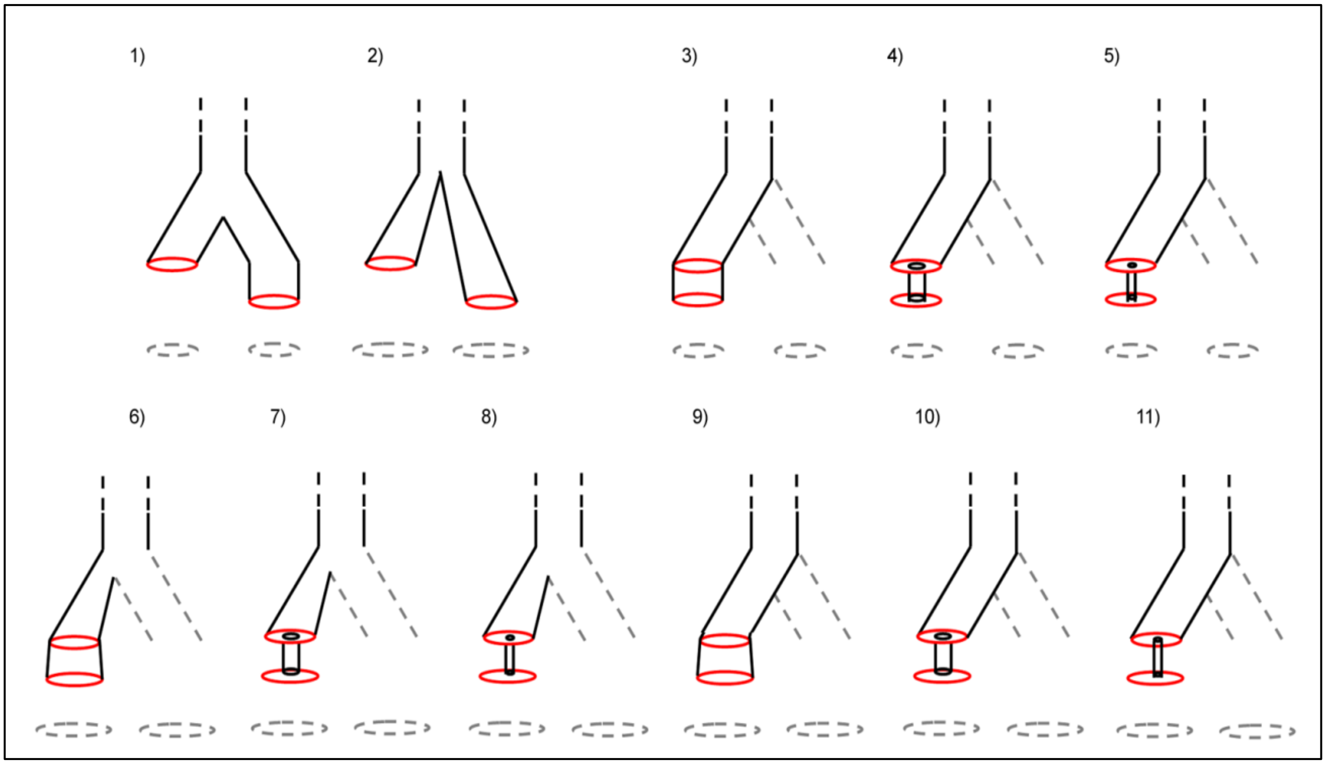

Supplementary Fig. 2. Eleven candidate scenarios for the demographic history underlying two population samples taken from the Eastern Scythians. These samples (taken at *t* = 94 g BP and *t* = 108 g BP) can be derived from two different populations (with size *N*) that previously split (at *t* = 108 - 4,000 g BP, scenarios 1 and 2) or derived from the same genetically continuous population (with size *N*) (scenarios 3–11). Moreover, these populations can be of constant size (scenarios 1, 3–5) or expanding (with growth rate *r*) (scenarios 2, 6–11) where the onset of population expansion can be modelled as occurring at the time of the oldest eastern Scythian sample (scenarios 9–11) or earlier (scenarios 2, 6–8), Finally, populations may have undergone bottlenecks during the period between the two sampling time points. These bottlenecks were moderate (10% in scenarios 4, 7 and 10) or severe in size (1% in scenarios 5, 8 and 11). Please note that the timing of demographic events is not displayed to scale.

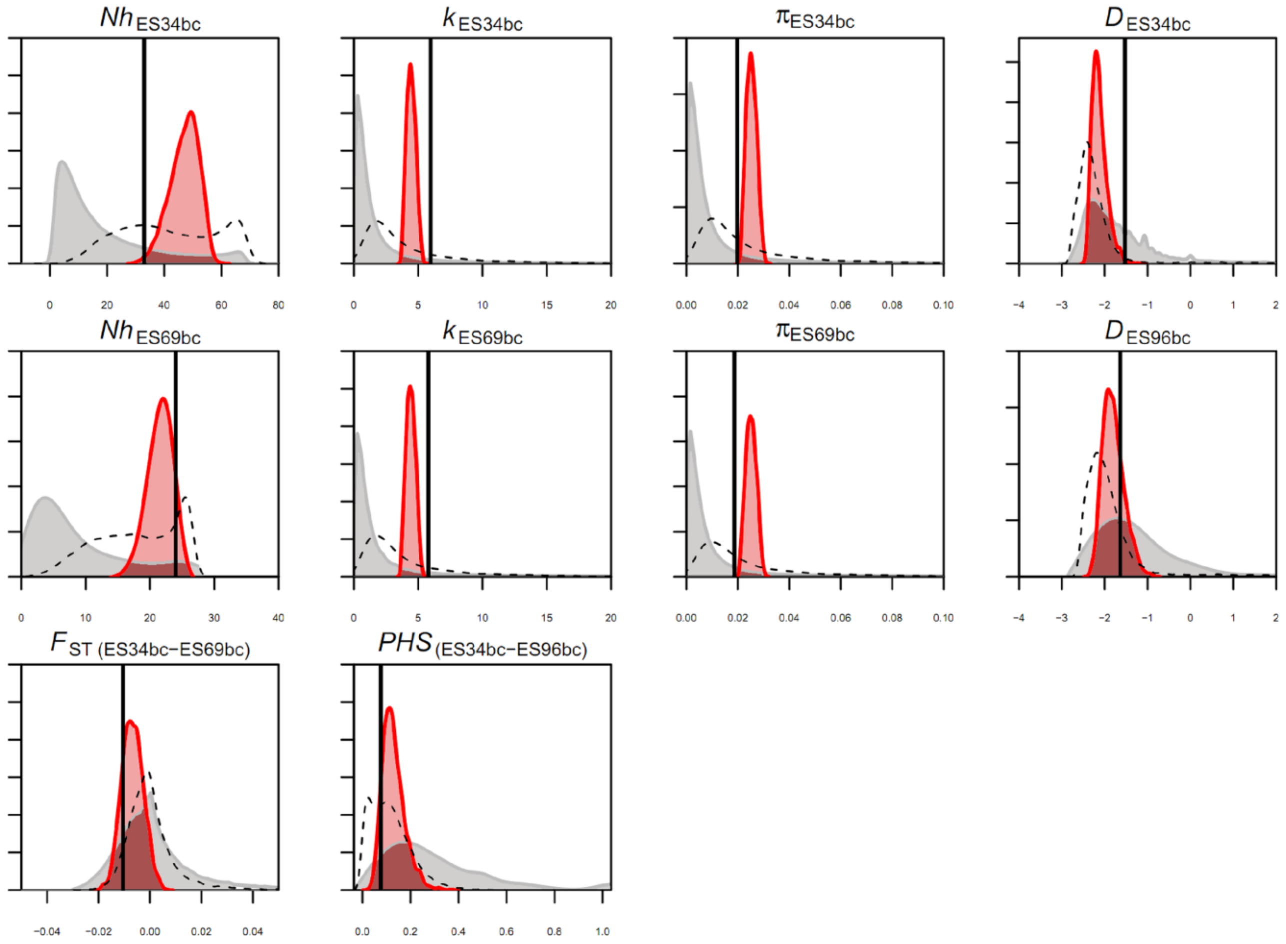

Supplementary Fig. 3. Prior and posterior distributions of 10 summary statistics used for the *ABC* analyses involving eastern Scythian sample groups. For two sample groups, taken at t = 94 g BP (ES34bc) and t = 108 g BP (ES69bc), within-population summary statistics include the number of haplotypes (*Nh*), the mean number of pairwise difference (*k*), nucleotide diversity (π) and Tajima's *D*. Between-population summary statistics include genetic differentiation (F_{ST}) and the percentage of haplotypes shared (*PHS*). For each summary statistic, prior distributions (grey) and posterior distributions (red) are given, as well as the values observed for the empirical data (solid black vertical lines). Posterior distributions represent values retained by the model selection procedure (these were subsequently used for model parameter posterior estimation). Finally, for the best-fitting model, the distributions of summary statistics simulated under model parameters drawn post-hoc from their own posterior distributions are also included (black dashed lines).

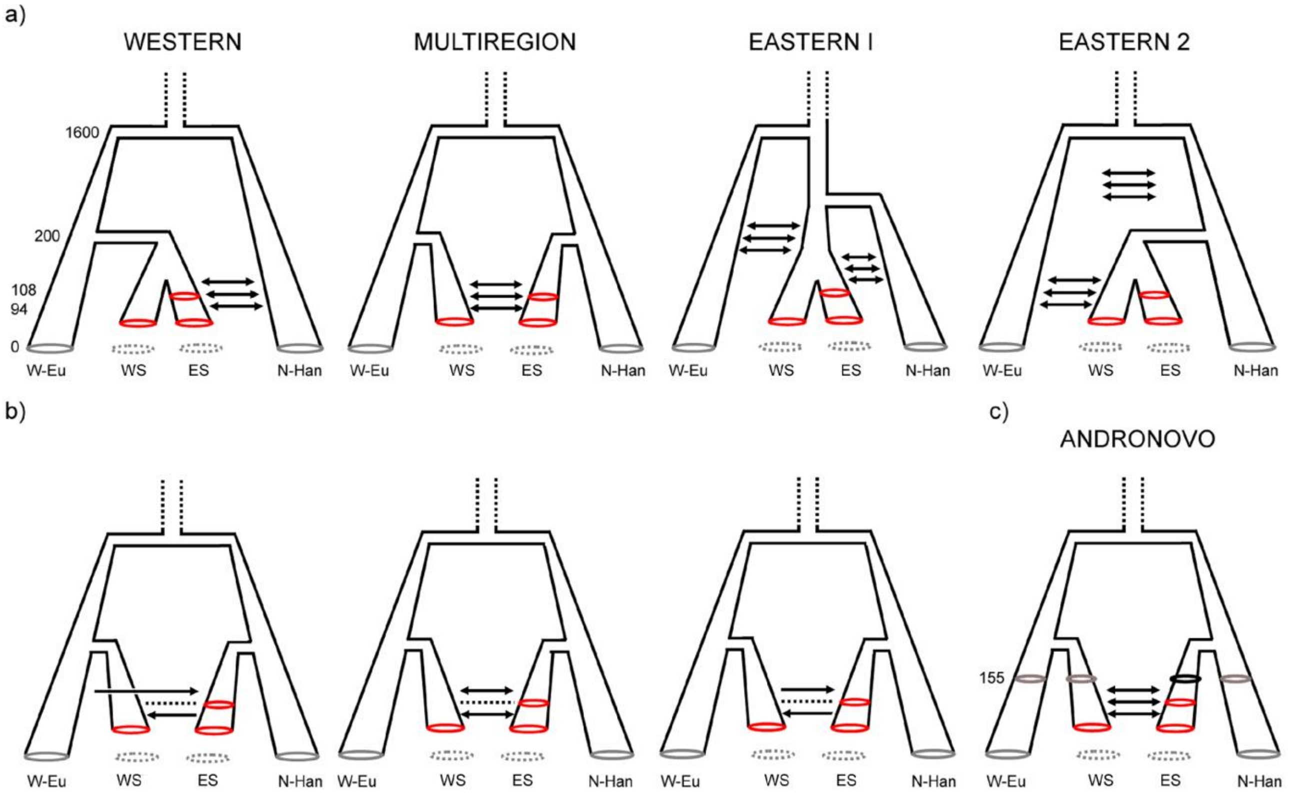

Supplementary Fig. 4. Overview of candidate scenarios for the origins of, and relations between, Scythian populations from the Iron Age. (a) In the first analysis, four candidate scenarios were evaluated, where the western (WS) and eastern (ES) populations are descended either from western Europeans (W-Eu) or Han Chinese (N-Han), while potentially exchanging gene flow. The most recent common ancestor of all these populations is assumed to be at 1600 g BP (~40 ky BP). The timing of population splits and sampling are displayed on the left (in g BP). (b) Additional scenarios evaluating the timing of gene flow compared to the preferred population tree from the first analysis (Multi-region) (c) Analysis fitting a Bronze Age sample of Andronovo/Fedorovo (taken on average at t = 155 g BP). This sample was evaluated as being putatively ancestral to western Europeans, western Scythians, eastern Scythians or Han Chinese. See Supplementary Note 1 for full explanation. Please note that the timing of demographic events is not displayed to scale.

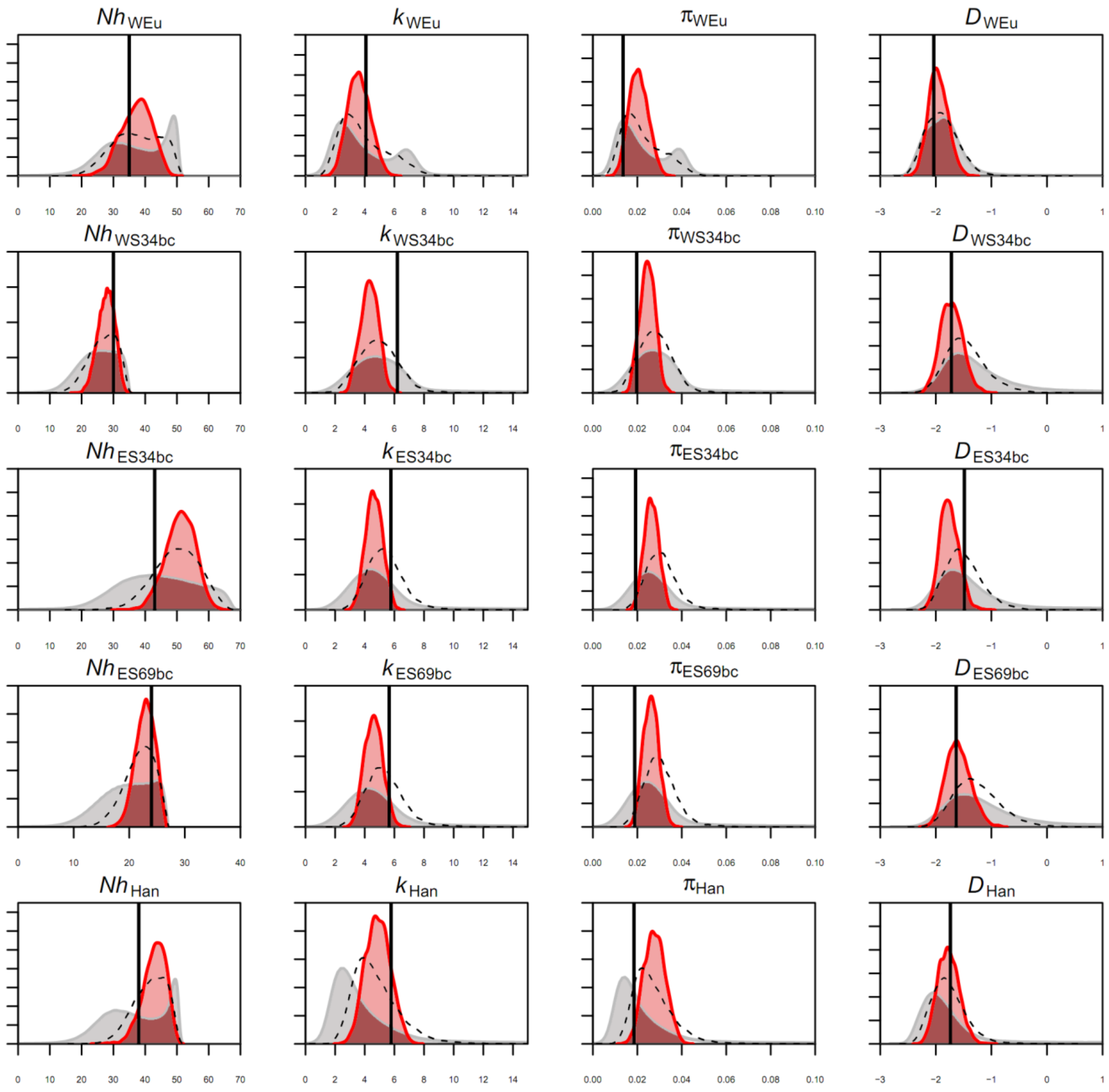

Supplementary Fig. 5. Prior and posterior distributions of summary statistics used for the *ABC* analyses on the origins of Scythian populations from the Iron Age. Included are sample groups from western Scythians (WS34bc) and eastern Scythians (ES34bc and ES69bc), in relation to representative samples from Western Europe (WEu) and Han Chinese (Han). For each population sample, within-population summary statistics include the number of haplotypes (*Nh*), the mean number of pairwise difference (*k*), nucleotide diversity (π) and Tajima's *D*. Between-population summary statistics include genetic differentiation (F_{ST}) and the percentage of haplotypes shared (*PHS*). For each summary statistic, the prior (grey) and posterior distributions (red) are given, as well as empirically observed values (solid black vertical lines). Finally, for the best-fitting model, distributions of summary statistics simulated under model parameters drawn post-hoc from their posterior distributions are also given (black dashed lines).

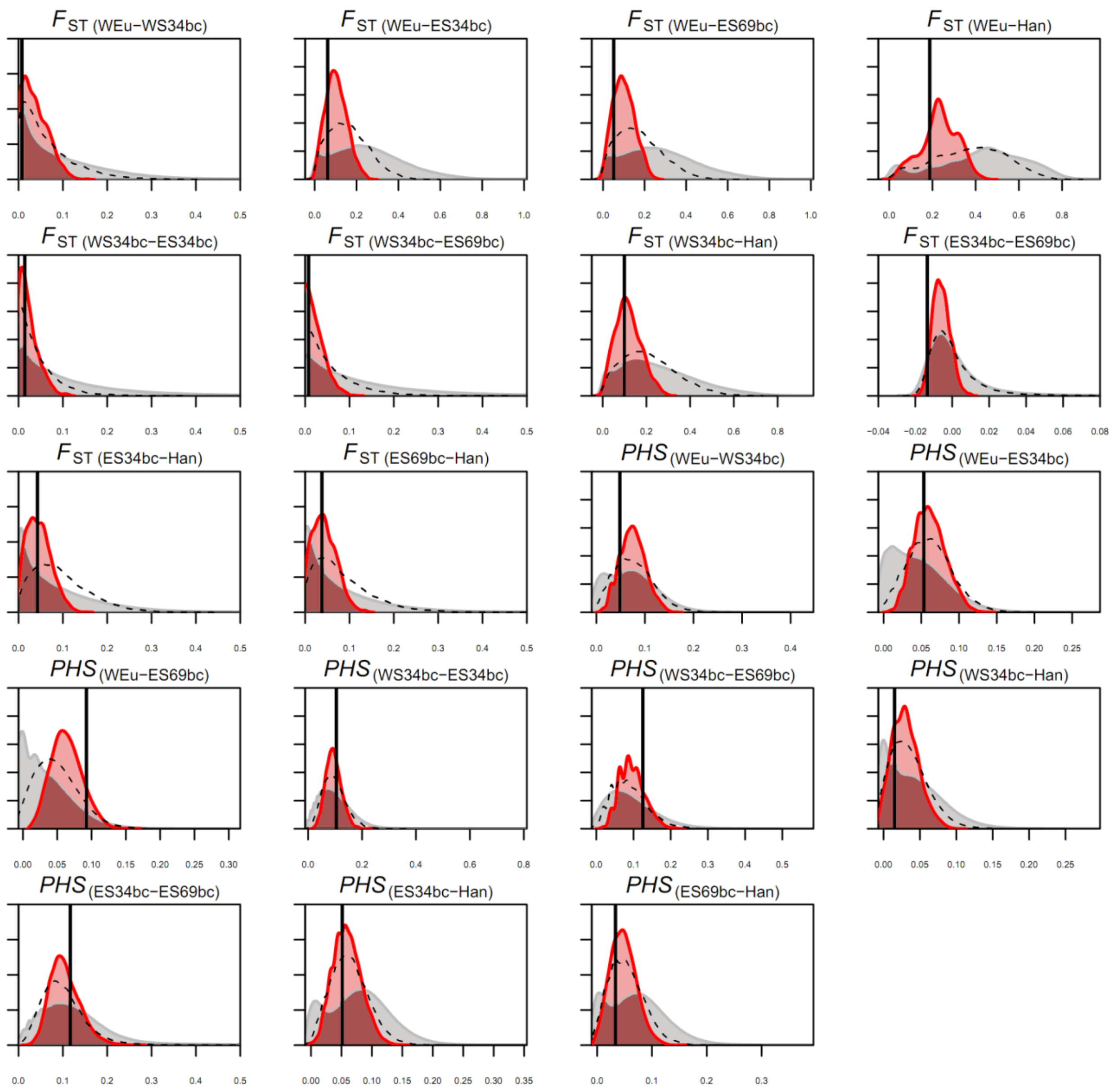

Supplementary Fig. 5 *continued*

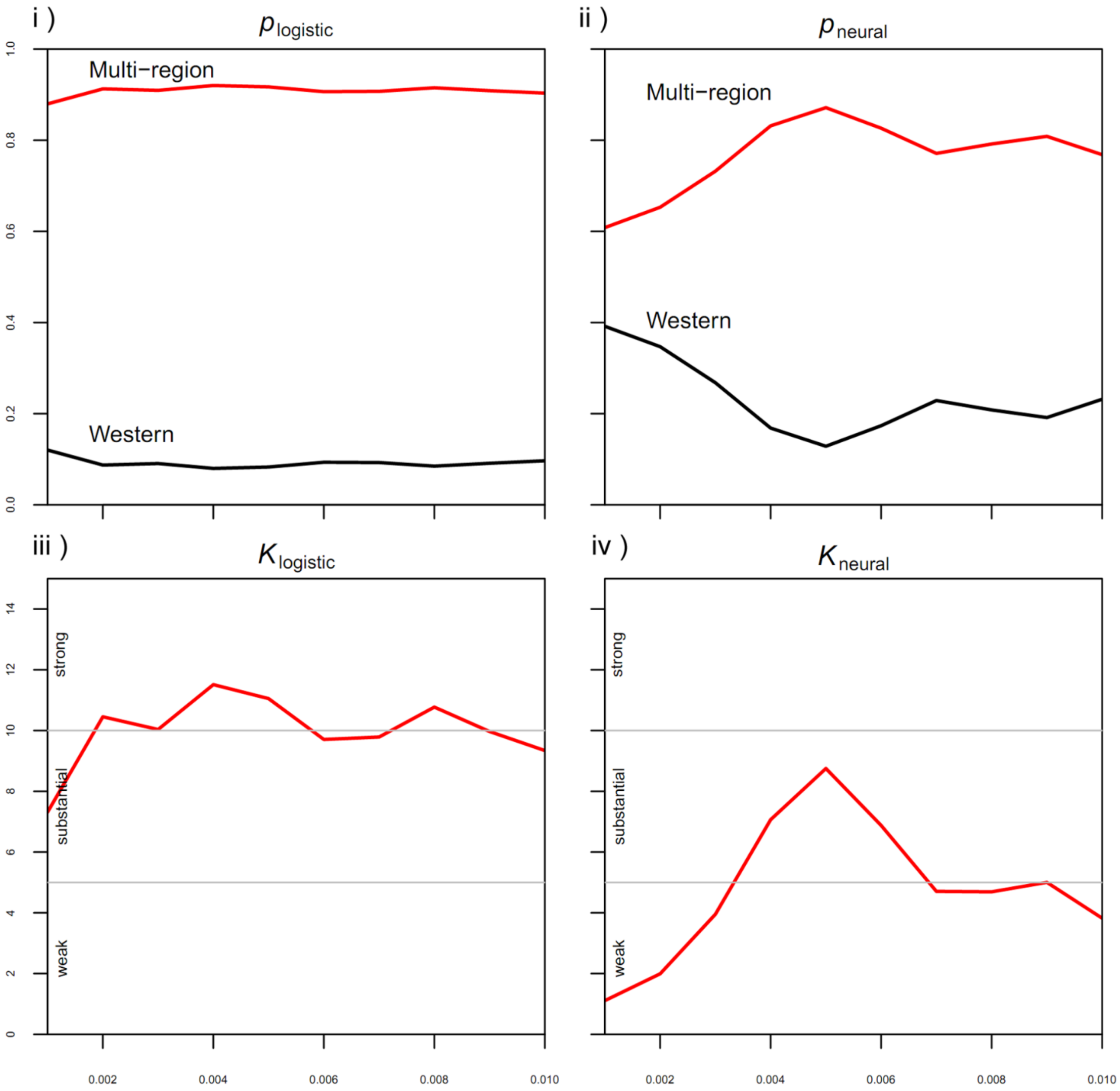

Supplementary Figure 5b. Detailed model selection procedure considering only a two-way comparison between the western and multiregion models of Scythian origins. Given are the model posterior estimates (*p*) for both models (in black and red, respectively) under the logistic regression method (panel i) and the neural networks method (panel ii), over a range of tolerance rates. In addition is given the Bayes factor (*K*), for each comparison and model selection method (panels iii, iv) which scales the plausibility of the multi-region model relative to that of the western model. Interpretation scales are added for clarity: values of *K* indicate weak (0-5), substantial (5-10) or strong (10-15) support for the preferred (here: multi-region) model. All analyses conducted using the package *abc*[28] in R v.2.15.1[29].

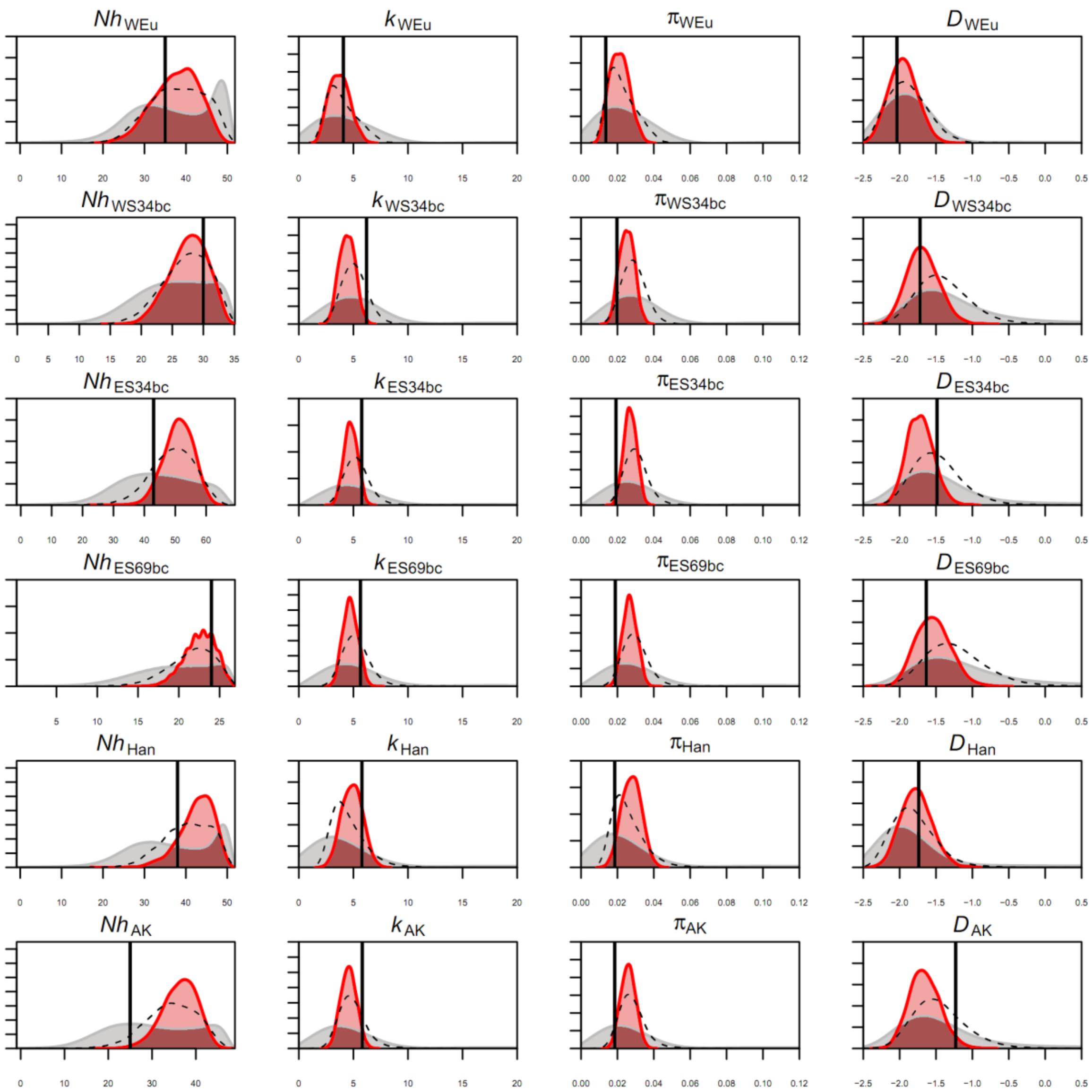

Supplementary Fig. 6. Prior and posterior distributions of 54 summary statistics considered for the ABC analyses on the relationship between Bronze Age nomadic groups and Iron Age Scythian populations. Considered here were samples from western Scythians (WS34bc) and eastern Scythians (ES34bc and ES69bc), and a sample consisting mainly of Andronovo and Karasuk (AK), in relation to representative samples from Western Europe (WEu) and Han Chinese (Han). Within-population summary statistics include the number of haplotypes (*Nh*), the mean number of pairwise difference (*k*), nucleotide diversity (π) and Tajima's *D*. Between-population summary statistics include genetic differentiation (F_{ST}) and the percentage of haplotypes shared (*PHS*). For each summary statistic, the prior (grey) and posterior distributions (red) are given, as well as empirically observed values (solid black vertical lines). Finally, for the best-fitting model, distributions of summary statistics simulated under model parameters drawn post-hoc from their posterior distributions are also given (black dashed lines).

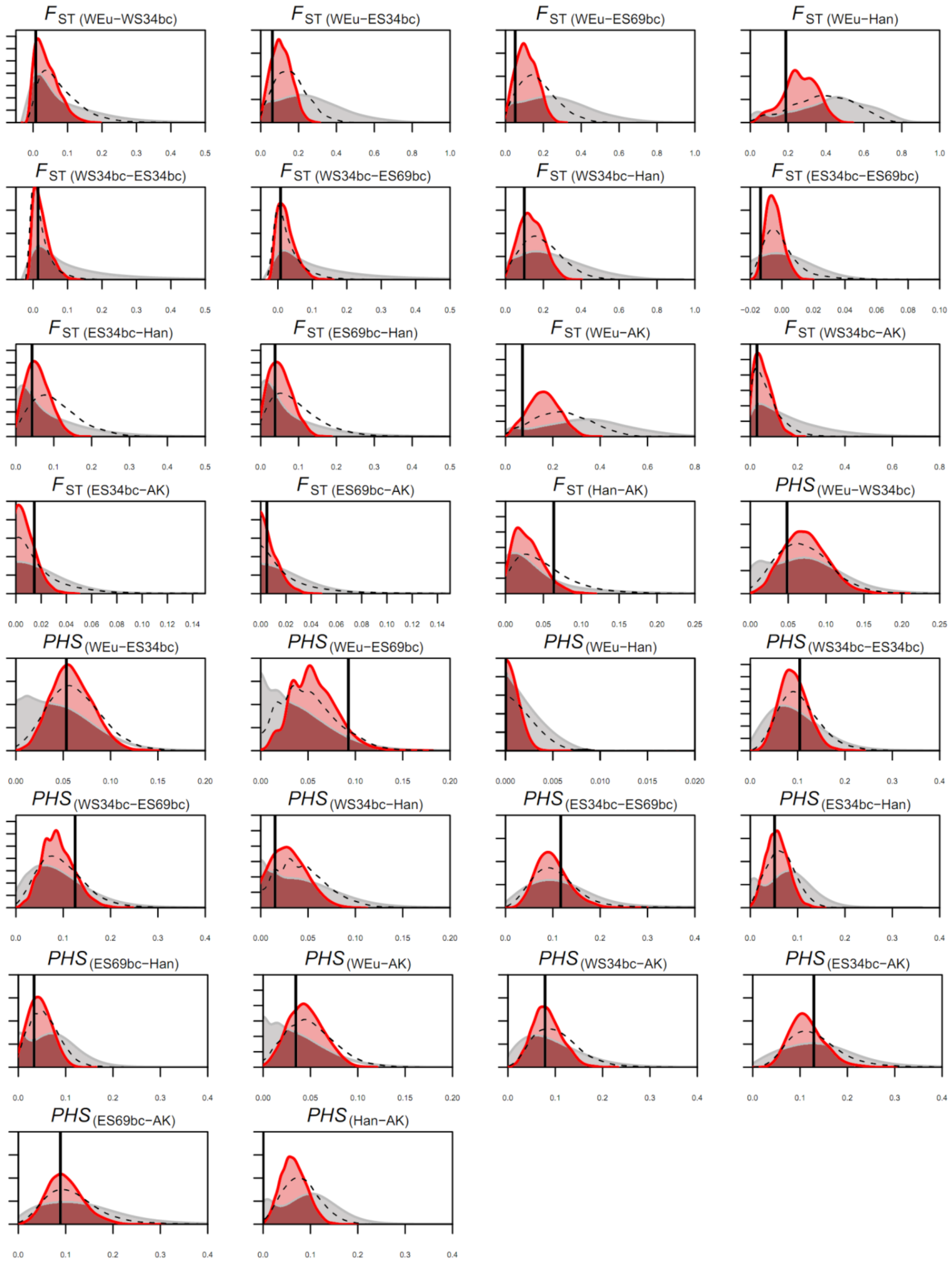

Supplementary Fig. 6 ***continued***

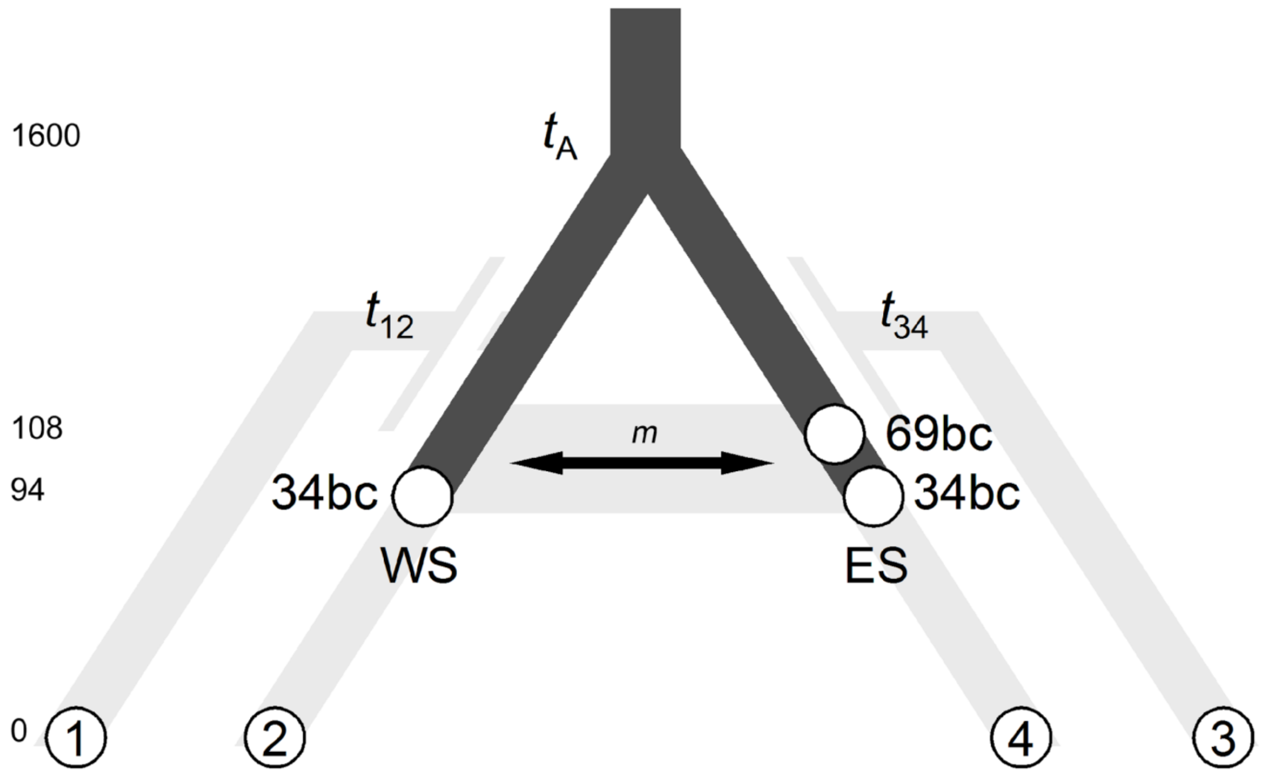

Supplementary Fig. 7. Four demographic scenarios that can explain the origins of contemporary Eurasian populations in relation to Bronze and Iron Age Scythian populations. The western Scythian (WS) and eastern Scythian (ES) populations are presumed to have split at t_A (1600 g BP), and to have exchanged migrants (roughly) during the last millennium BC. Contemporary populations could have descended directly from these Scythian populations (scenarios 2 and 4) or share a common ancestor in the more distant past (scenarios 1 and 3). The range of these latter splitting times (t_{12} and t_{34}), was sampled from the range t_{min} - 800 g BP. The minimum splitting time (t_{min}) was chosen in order to attain sufficient power to distinguish between each pair of competing candidate scenarios (see Supplementary Fig. 8).

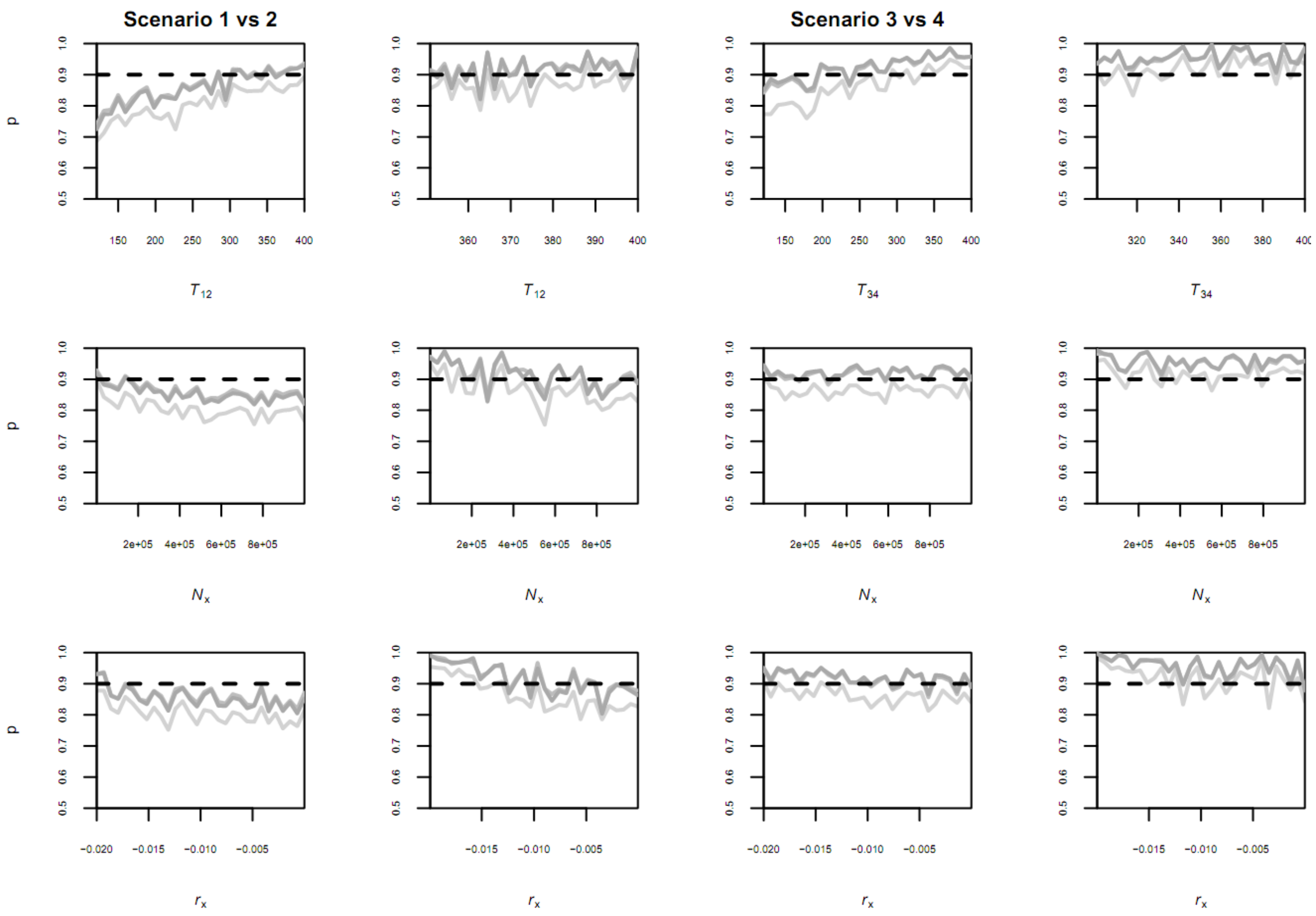

Supplementary Fig. 8. Power to identify a scenario of ancestral relatedness as a function of splitting time (*T*), population size (*N*) and population growth rate (*r*), for simulated contemporary population *x*. Results are given as posterior model probabilities for the correct scenario, for the comparison between scenarios 1 (ancestral to western Scythians) and 2 (descent from western Scythians) (first column). The same results are given for the model selection involving scenarios 3 (ancestral to eastern Scythians) and 4 (descent from eastern Scythians) (third column). Probabilities are shown for two different model selection methods: a logistic regression method (light grey lines) and a non-linear neural networks method (dark grey lines). Posterior probabilities higher than 0.9 were used as a cut-off value to determine the minimal splitting time (t_{min}) for subsequent simulations, resulting in $t_{12(min)}$ > 350 g BP and $t_{34(min)}$ > 300 g BP. As can be seen for scenarios 1-2 (second column) and scenarios 3-4 (fourth column), simulations with these values of t_{min} resulted in high confidence to correctly identify ancestral relatedness. These results illustrate a generally higher power to correctly identify ancestry to the eastern Scythian sample than ancestry to the western Scythian sample. Furthermore, effective population size (*N*) and growth rate (*r*) of the target population *x* had little influence on statistical power. Results based on 3000 evaluations per splitting time.

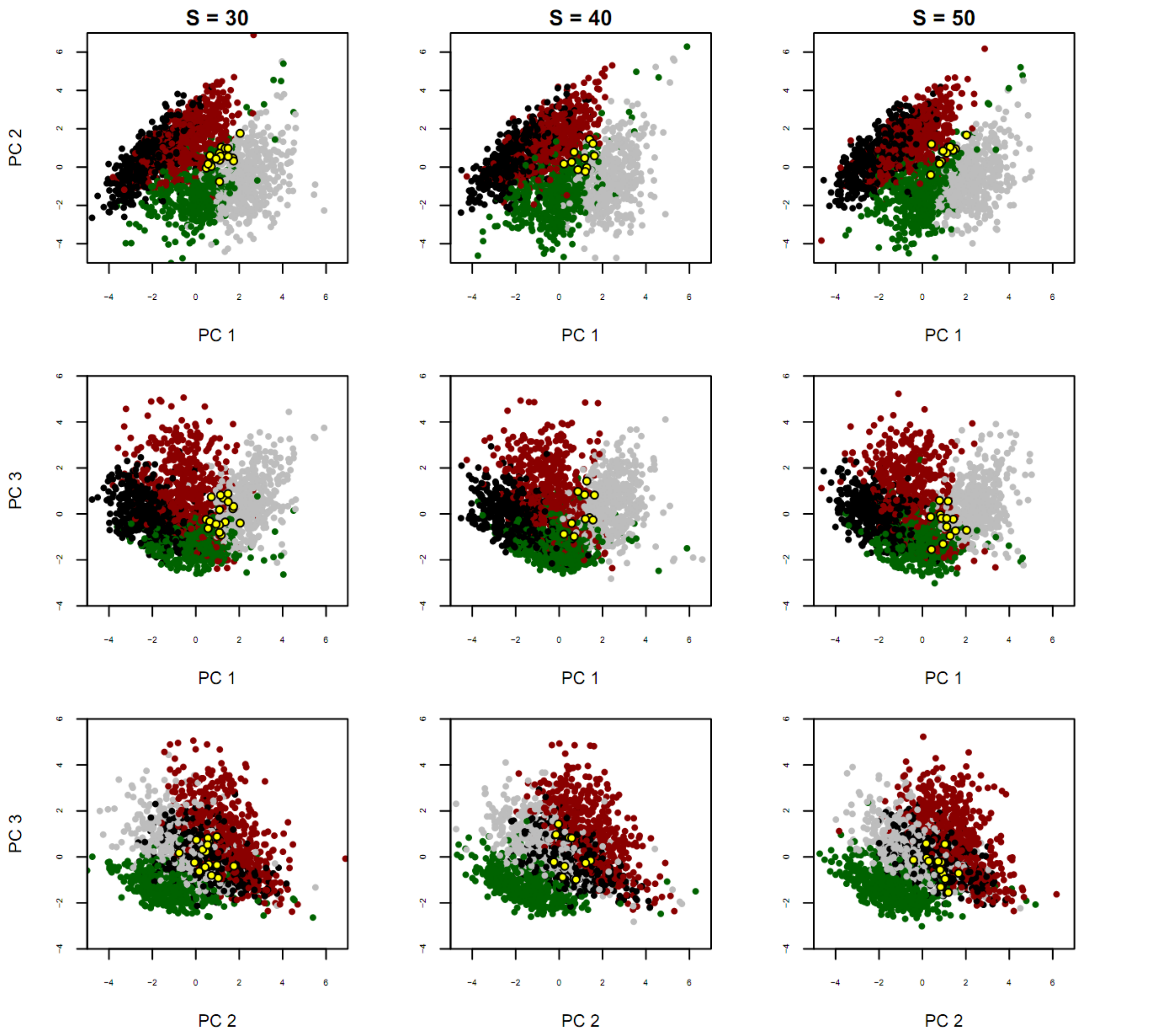

Supplementary Fig. 9. Fit of simulated data to observed summary statistics, for populations simulated with sample sizes S=30, S=40 and S=50. Given are simulated data points for scenario 1 (ancestral to western Scythians, black), scenario 2 (descent from western Scythians, dark red), scenario 3 (ancestral to eastern Scythians, green) and scenario 4 (descent from eastern Scythians, grey). Also plotted are observed summary statistics for contemporary populations (yellow). The first three components of the PCA explain approximately 70% of overall observed variance.

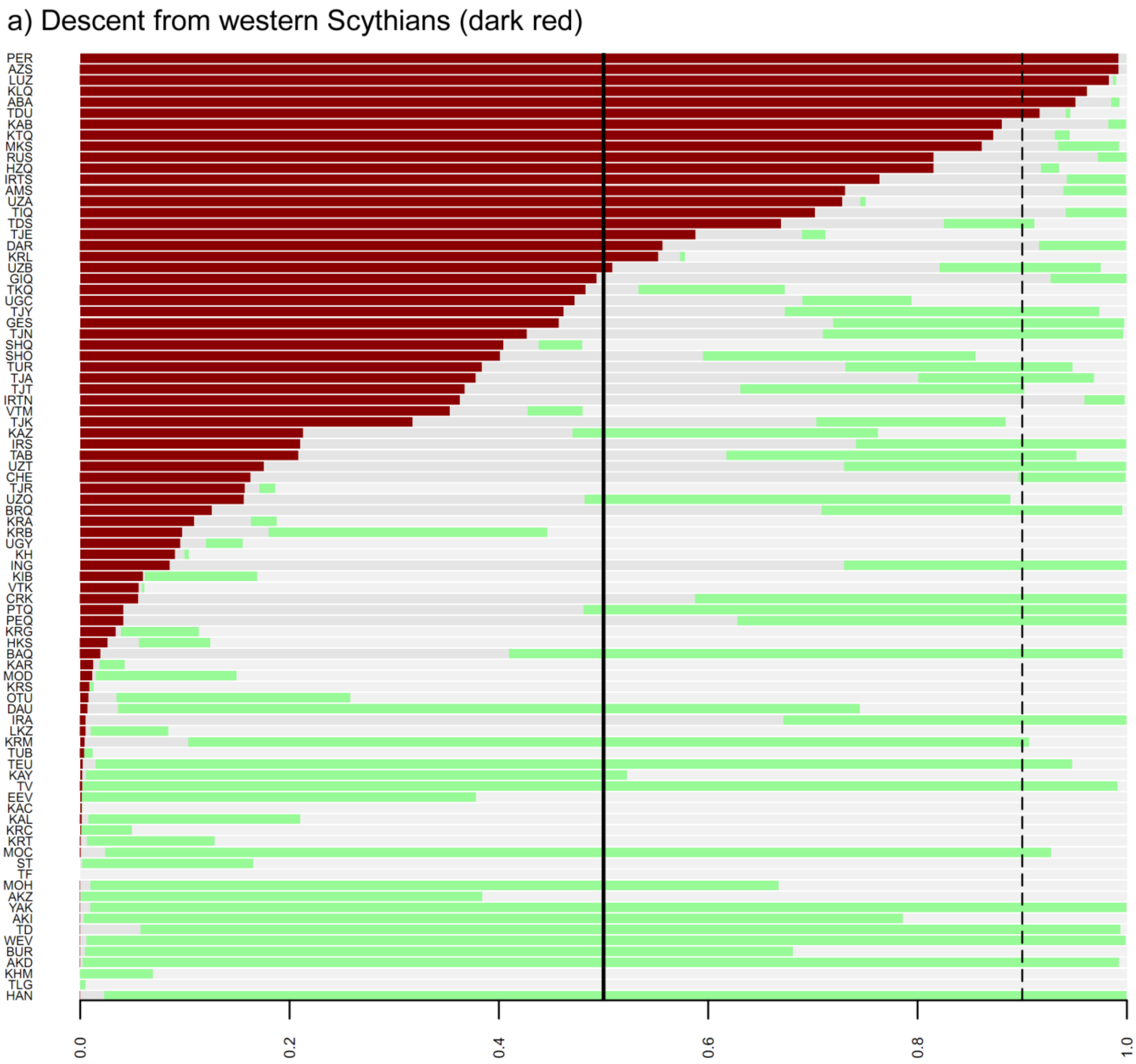

Supplementary Fig. 10. Posterior probabilities of four candidate scenarios of relation to Iron Age Scythians for 86 contemporary Eurasian populations. Each panel shows model posteriors sorted by a) descent from western Scythians (dark red), b) descent from eastern Scythians (dark grey), c) ancestral relatedness to western Scythians (black) and d) ancestral relatedness to eastern Scythians (dark green). Colours of other scenarios are dimmed in these panels to aid in interpretation. The horizontal bars represent the 50% and 90% cut-off values for model posterior probability. See Supplementary Table 19 for population codes on the left.

b) Descent from eastern Scythians (dark grey)

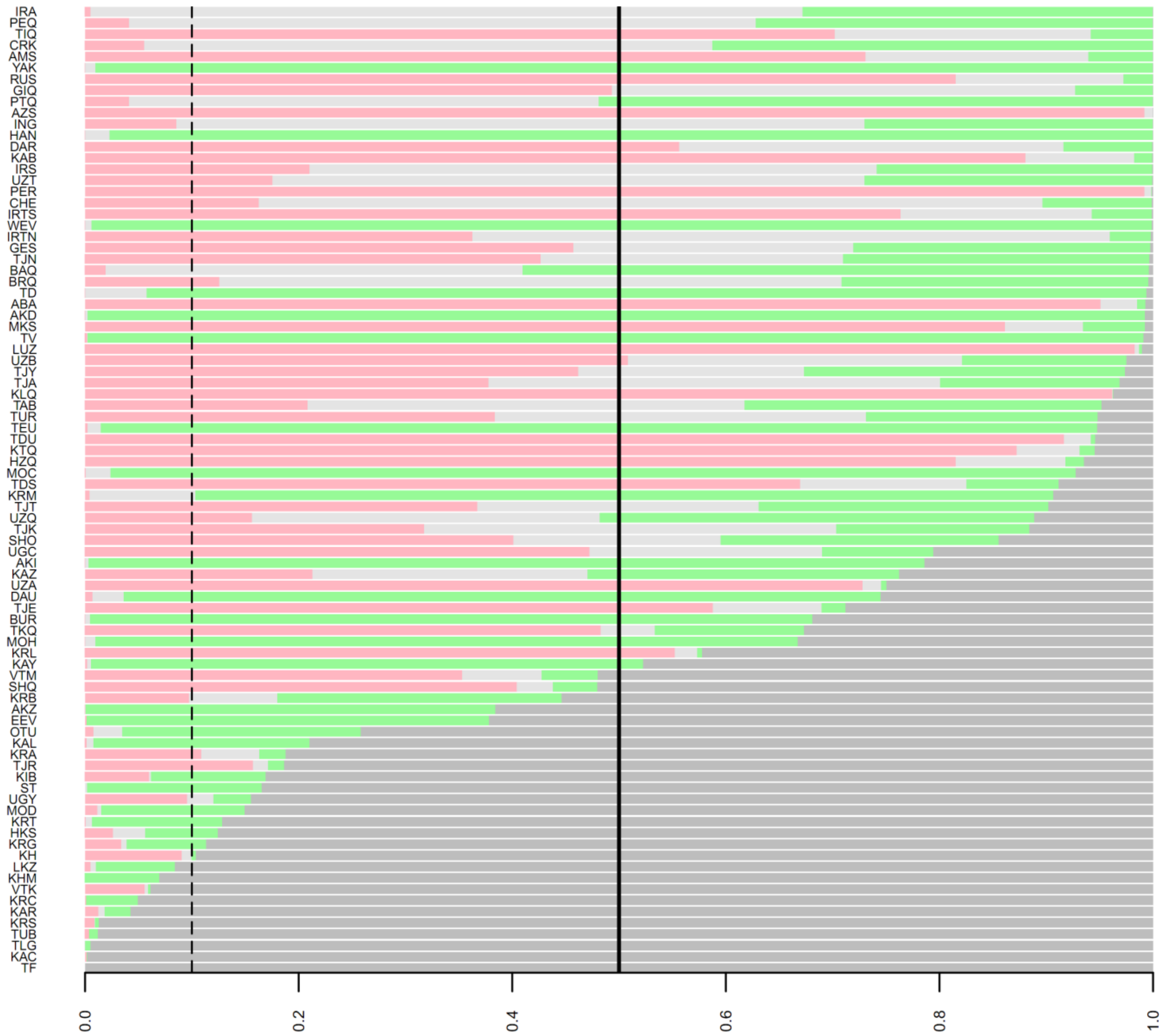

Supplementary Fig. 10 ***continued***

c) Ancestral relatedness to western Scythians (black)

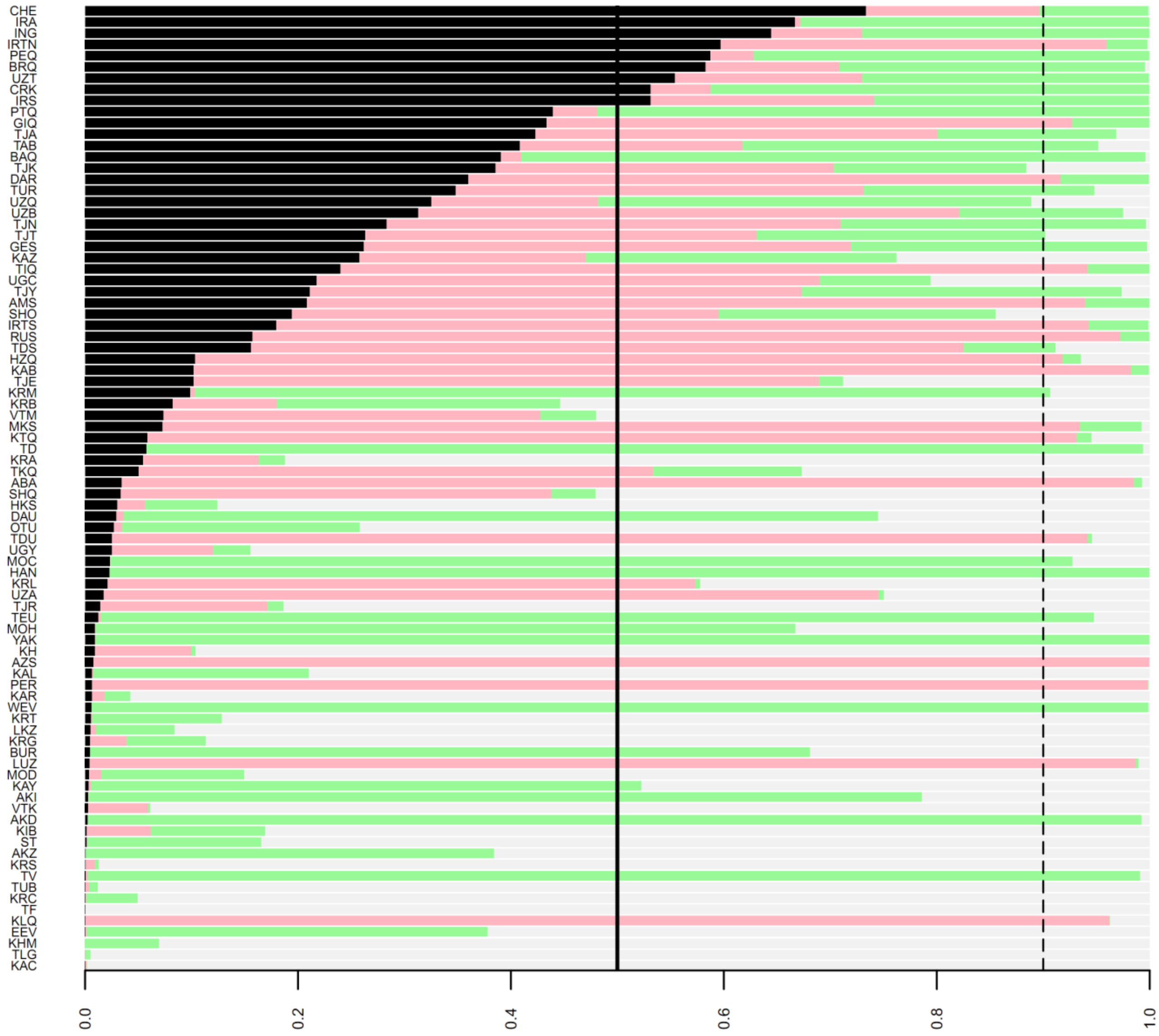

Supplementary Fig. 10 *continued*

d) Ancestral relatedness to eastern Scythians (dark green)

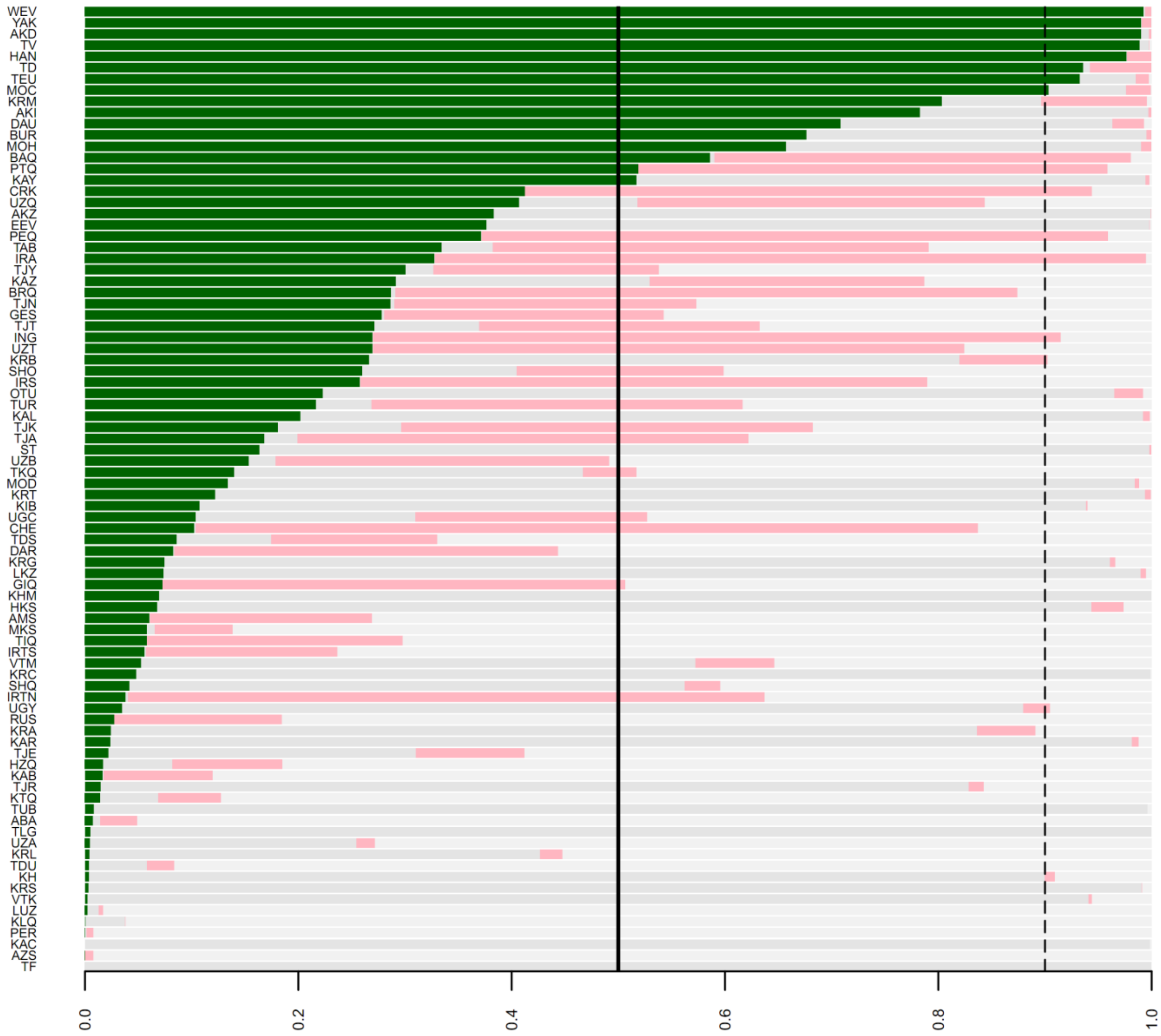

Supplementary Fig. 10 ***continued***

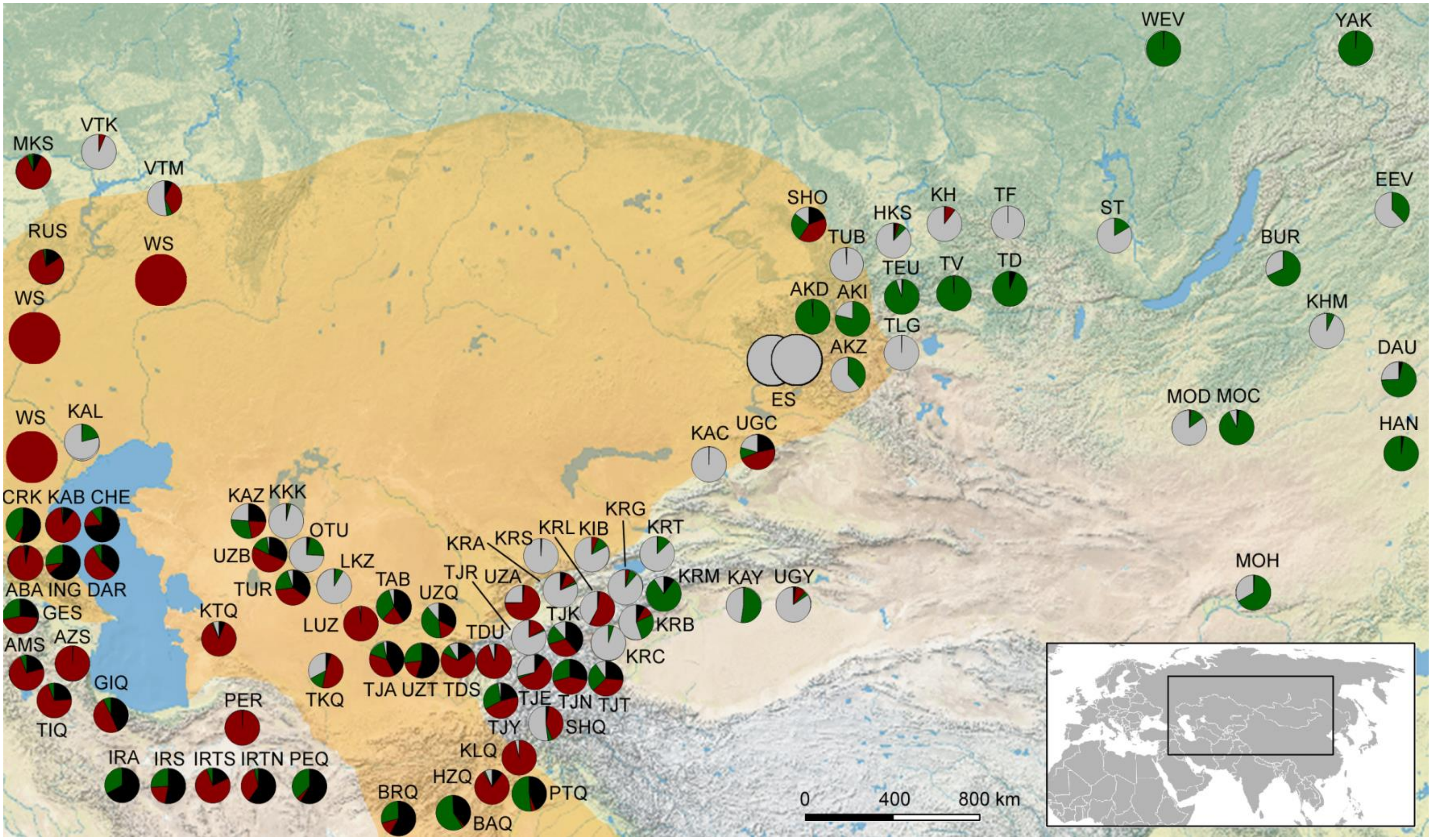

Supplementary Fig. 11. Model posteriors for descent from Scythian populations for 86 contemporary human populations in Central Asia. Given for each contemporary sample are pie-charts representing the model posteriors for descent from western Scythians (black), descent from eastern Scythians (grey) ancestral relatedness to western Scythians (red) or eastern Scythian groups (green). Also given are the approximate locations of the ancient DNA samples and the historical range of Iron Age Scythian tribes (orange area). See Supplementary Table 19 for detailed information on contemporary populations. Source: underlying map was created by Tom Patterson, and downloaded from http://www.shadedrelief.com.

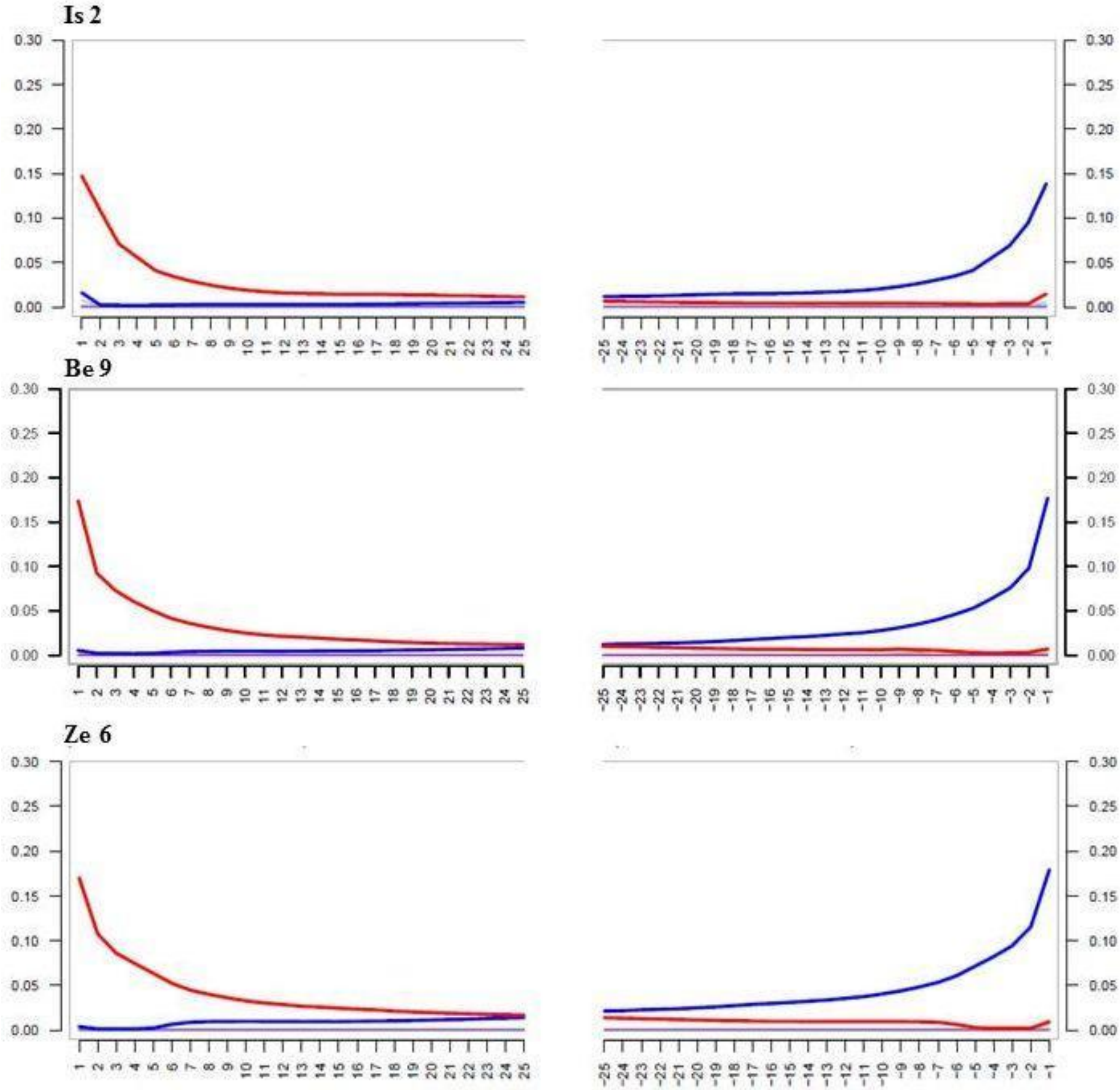

Supplementary Fig. 12. Damage patterns of the shotgun samples. Damage patterns were generated with mapDamage 2.0[62] of the three shotgun samples Is2, Be9 and Ze6 (from top to bottom); damage is shown as C to T (red line) or G to A (blue line) transition rates relative to the position of the mismatch in a read.

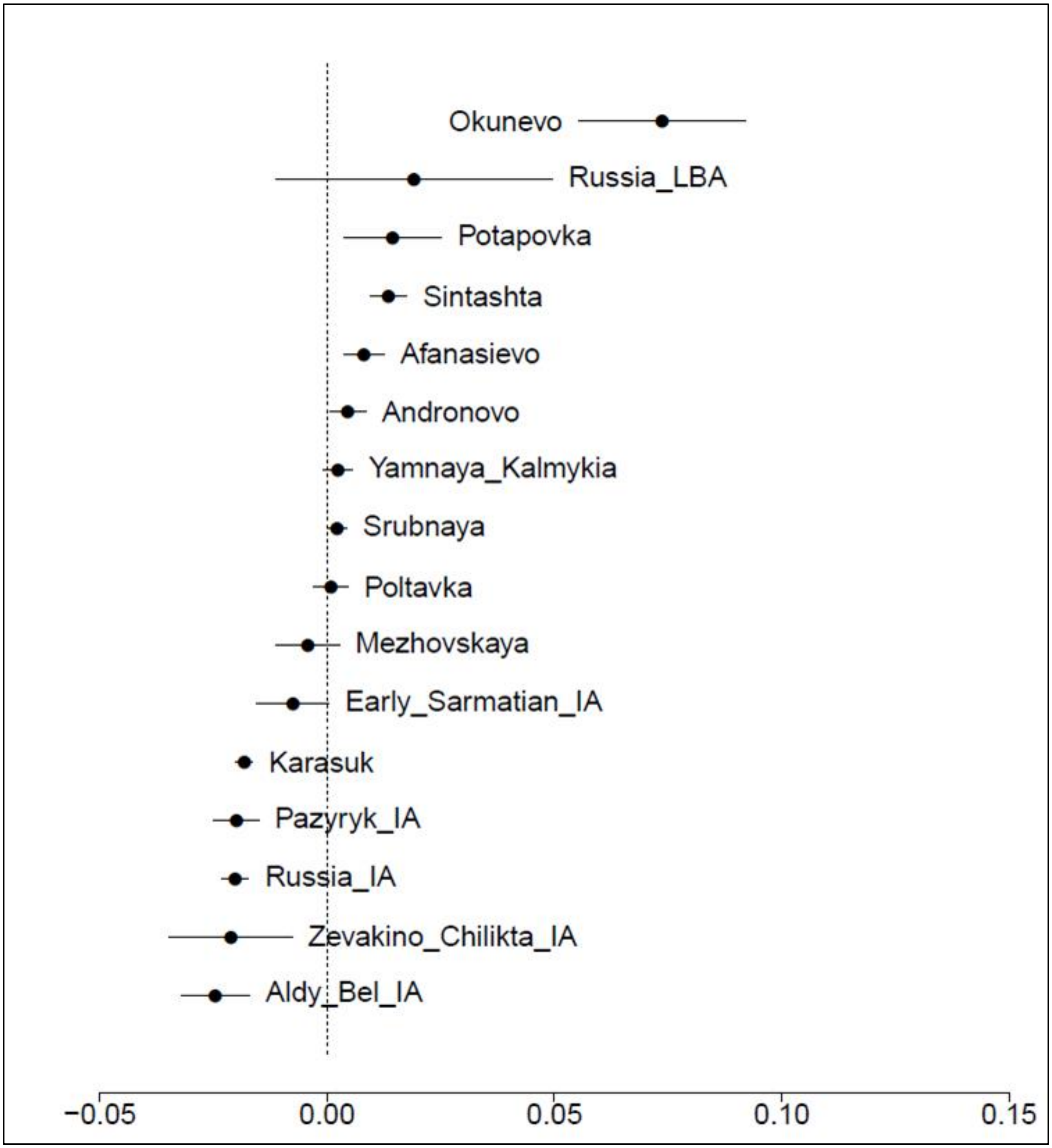

Supplementary Fig. 13. Results for *f*3(Test; Yamnaya_Samara, Han). The values ordered by size, negative values signify admixture between Yamnaya-like and Han-like populations in ancestry of Test population.

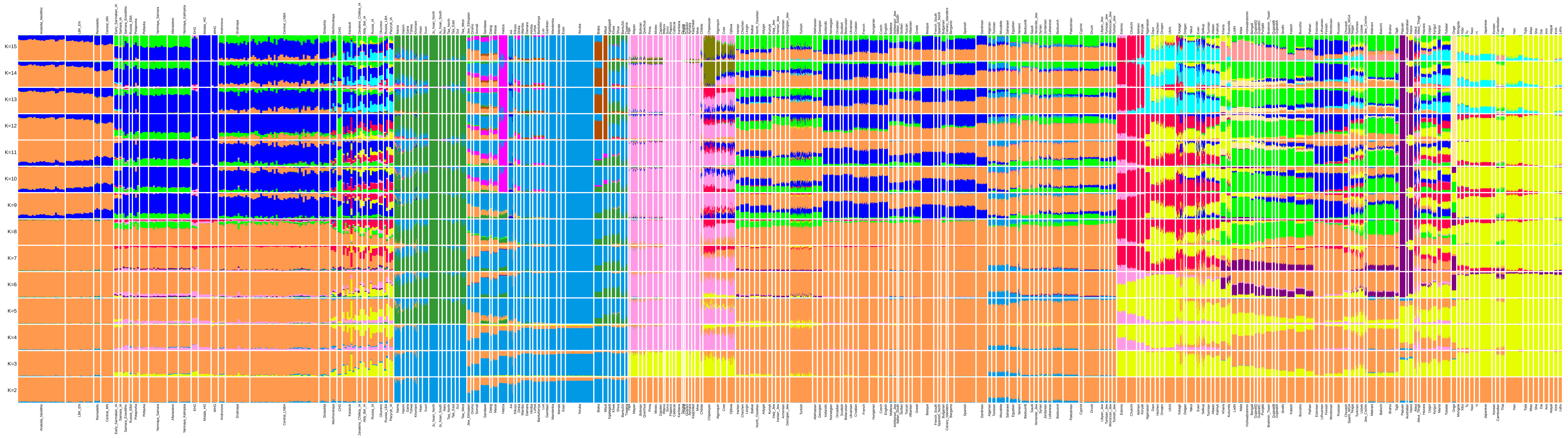

Supplementary Fig. 14. ADMIXTURE result for ancient populations. ADMIXTURE results are shown for all tested populations from K=2 to K=15.

Supplementary Table 1. Sample material, assignment and analyses

Samples analysed for this study including data from the literature are listed according to their geographical region, dating and cultural assignment. Specified in the last columns are the analyses that have been performed: ABC: HVR1 sequences were used for Approximate Bayesian computation; capture: genome capture has been performed; samples labelled with shotgun were used for shotgun analyses.

Steppe	*Region*	*Culture*	*Dating*	*Site/Area*	*Lab code*	*Source*	*Haplogroup***	*Analyses*		
East	East Kazakhstan	Zevakino-Chilikta	9th–7th c. BCE	Ismailovo	Is_1	this study	D4h1	ABC		
	East Kazakhstan	Zevakino-Chilikta	9th–7th c. BCE	Ismailovo	Is_2	this study	HV-CRS	ABC		shotgun
	East Kazakhstan	Zevakino-Chilikta	9th–7th c. BCE	Ismailovo	Is_4	this study	H2a1	ABC		
	East Kazakhstan	Zevakino-Chilikta	9th–7th c. BCE	Zevakino	Ze_2	this study	T2b	ABC		
	East Kazakhstan	Zevakino-Chilikta	9th–7th c. BCE	Zevakino	Ze_3	this study	K1	ABC		
	East Kazakhstan	Zevakino-Chilikta	9th–7th c. BCE	Zevakino	Ze_4	this study	D4	ABC		
	East Kazakhstan	Zevakino-Chilikta	9th–7th c. BCE	Zevakino	Ze_5	this study	I	ABC		
	East Kazakhstan	Zevakino-Chilikta	9th–7th c. BCE	Zevakino	Ze_6	this study	C4	ABC		shotgun
	East Kazakhstan	Zevakino-Chilikta	9th–7th c. BCE	Zevakino	Ze_7	this study	U4a1	ABC		
	East Kazakhstan	Zevakino-Chilikta	9th–7th c. BCE	Zevakino	Ze_8	this study	D4	ABC		
	East Kazakhstan	Zevakino-Chilikta	9th–7th c. BCE	Zevakino	Ze_9	this study	D4j3	ABC		
	Tuva	Aldy Bel	7th–6th c. BCE	Arzhan 2	A_2	this study	A8	ABC		
	Tuva	Aldy Bel	7th–6th c. BCE	Arzhan 2	A_3	this study	G	ABC		
	Tuva	Aldy Bel	7th–6th c. BCE	Arzhan 2	A_4	this study	D4b1a2a	ABC		
	Tuva	Aldy Bel	7th–6th c. BCE	Arzhan 2	A_5	this study	C5b1	ABC		
	Tuva	Aldy Bel	7th–6th c. BCE	Arzhan 2	A_6	this study	U5a1f1	ABC		
	Tuva	Aldy Bel	7th–6th c. BCE	Arzhan 2	A_7	this study	Y1	ABC		
	Tuva	Aldy Bel	7th–6th c. BCE	Arzhan 2	A_8	this study	U4a3	ABC		
	Tuva	Aldy Bel	7th–6th c. BCE	Arzhan 2	A_9	this study	U5a1d2b	ABC		
	Tuva	Aldy Bel	7th–6th c. BCE	Arzhan 2	A_10	this study	A	ABC	capture	
	Tuva	Aldy Bel	7th–6th c. BCE	Arzhan 2	A_11	this study	G	ABC		
	Tuva	Aldy Bel	7th–6th c. BCE	Arzhan 2	A_14	this study	T1a	ABC		
	Tuva	Aldy Bel	7th–6th c. BCE	Arzhan 2	A_17	this study	H	ABC	capture	
	Tuva	Aldy Bel	7th–6th c. BCE	Arzhan 2	A_19	this study	C4	ABC		
	Tuva	Aldy Bel	7th–6th c. BCE	Arzhan 2	A_20	this study	G2a	ABC		
	Tuva	Aldy Bel	7th–6th c. BCE	Arzhan 2	A_21	this study	A4	ABC		
	Khakassia	Tagar	5th c. BCE	Barsucij Log	BL_1	this study	C5+16093			
	Khakassia	Tagar	5th c. BCE	Barsucij Log	BL_2	this study	U5a			

Steppe	*Region*	*Culture*	*Dating*	*Site/Area*	*Lab code*	*Source*	*Haplogroup***	*Analyses*		
	Khakassia	Tagar	5th c. BCE	Barsucij Log	BL_3	this study	C5+16093			
	Khakassia	Tagar	5th c. BCE	Barsucij Log	BL_4	this study	C5+16093			
	Khakassia	Tagar	5th c. BCE	Barsucij Log	BL_5	this study	U2e2			
	Khakassia	Tagar	5th c. BCE	Barsucij Log	BL_6	this study	A4			
	Khakassia	Tagar/Tes	8th c. BCE – 1st c. CE	Anach	S21	15	T2b2b			
	Khakassia	Tagar/Tes	8th c. BCE – 1st c. CE	Anach	S22	15	T2b2b			
	Khakassia	Tagar/Tes	8th c. BCE – 1st c. CE	Chernogorsk	S23	15	T2c1			
	Khakassia	Tagar/Tes	8th c. BCE – 1st c. CE	Chernogorsk	S24	15	M25			
	Khakassia	Tagar/Tes	8th c. BCE – 1st c. CE	Oust-Abakabsty	S25	15	G2a			
	Khakassia	Tagar/Tes	8th c. BCE – 1st c. CE	Beysky region	S26	15	C5b1			
	Khakassia	Tagar/Tes	8th c. BCE – 1st c. CE	Bogratsky region	S27	15	CRS			
	Khakassia	Tagar/Tes	8th c. BCE – 1st c. CE	Bogratsky region	S28	15	F1b			
	Khakassia	Tagar/Tes	8th c. BCE – 1st c. CE	Bogratsky region	S29	15	CRS			
	Khakassia	Tagar/Tes	8th c. BCE – 1st c. CE	Bogratsky region	S32	15	H5			
	Kazakh Altai	Pazyryk	4th–3rd c. BCE	Berel'	Be_2	this study	D4	ABC		
	Kazakh Altai	Pazyryk	4th–3rd c. BCE	Berel'	Be_3	this study	H-CRS	ABC		
	Kazakh Altai	Pazyryk	4th–3rd c. BCE	Berel'	Be_4	this study	A	ABC		
	Kazakh Altai	Pazyryk	4th–3rd c. BCE	Berel'	Be_6	this study	A6	ABC		
	Kazakh Altai	Pazyryk	4th–3rd c. BCE	Berel'	Be_8	this study	D4	ABC		
	Kazakh Altai	Pazyryk	4th–3rd c. BCE	Berel'	Be_9	this study	A4f	ABC	capture	shotgun
	Kazakh Altai	Pazyryk	4th–3rd c. BCE	Berel'	Be_11	this study	C4a1+16129	ABC	capture	
	Kazakh Altai	Pazyryk	4th–3rd c. BCE	Berel'	Be_12	this study	HV2	ABC		
	Kazakh Altai	Pazyryk	4th–3rd c. BCE	Berel'	Be_14	this study	A6	ABC		
	Kazakh Altai	Pazyryk	4th–3rd c. BCE	Berel'	BeK11S1	69	CRS	ABC		
	Kazakh Altai	Pazyryk	4th–3rd c. BCE	Berel'	BeK11S2	69	D4g1	ABC		
	Kazakh Altai	Pazyryk	4th–3rd c. BCE	Tar Asu	Ta_1	this study	K2b1a	ABC		
	Russian Altai (Ukok)	Pazyryk	4th–3rd c. BCE	Ak Alakha 1	Ak1_1*	this study	C4a1+16129	ABC		
	Russian Altai (Ukok)	Pazyryk	4th–3rd c. BCE	Ak Alakha 4	Ak4_1	this study	A	ABC		
	Russian Altai (Ukok)	Pazyryk	4th–3rd c. BCE	Ak Alakha 5	Ak5_1*	this study	C4	ABC		
	Russian Altai (Ukok)	Pazyryk	4th–3rd c. BCE	Ak Alakha 5	Ak5_4*	this study	D4b1	ABC		
	Russian Altai (Ukok)	Pazyryk	4th–3rd c. BCE	Ak Alakha 5	Ak5_5*	this study	U2e1a	ABC		
	Russian Altai (Ukok)	Pazyryk	4th–3rd c. BCE	Ak Alakha 5	Ak5_6*	this study	U2e1a	ABC		
	Russian Altai (Ukok)	Pazyryk	4th–3rd c. BCE	Ak Alakha 5	Ak5_7*	this study	C4a1+16129	ABC		
	Russian Altai (Ukok)	Pazyryk	4th–3rd c. BCE	Ak Alakha 5	Ak5_8*	this study	A	ABC		
	Russian Altai (Ukok)	Pazyryk	4th–3rd c. BCE	Kuturguntas 1	K_1*	this study	H-CRS	ABC		

Steppe	*Region*	*Culture*	*Dating*	*Site/Area*	*Lab code*	*Source*	*Haplogroup***	*Analyses*		
	Russian Altai (Ukok)	Pazyryk	4th–3rd c. BCE	Moinak 2	Mo_1*	this study	D4b1a2a1	ABC		
	Russian Altai (Ukok)	Pazyryk	4th–3rd c. BCE	Moinak 2	Mo_2*	this study	D4h4a	ABC		
	Russian Altai (Ukok)	Pazyryk	4th–3rd c. BCE	Verch Kal'dzin 1	VK1_1	this study	K1	ABC		
	Russian Altai (Ukok)	Pazyryk	4th–3rd c. BCE	Verch Kal'dzin 2	VK2_K1	70	C	ABC		
	Russian Altai (Ukok)	Pazyryk	4th–3rd c. BCE	Verch Kal'dzin 2	VK2_K3	70	U5a1d2b	ABC		
	Russian Altai	Pazyryk	4th–3rd c. BCE	Balik Sook	BS_1	this study	X2b	ABC		
	Russian Altai	Pazyryk	4th–3rd c. BCE	Balik Sook	BS_2*	this study	U4b1a4	ABC		
	Russian Altai	Pazyryk	4th–3rd c. BCE	Kizil	95KBI52	71	N1a1a1a1a	ABC		
	Russian Altai (Chuya)	Pazyryk	4th–3rd c. BCE	Sebystei	SEB96K1	72	F2a	ABC		
	Russian Altai (Chuya)	Pazyryk	4th–3rd c. BCE	Sebystei	SEB96K2	72	D4	ABC		
	Russian Altai (Chuya)	Pazyryk	4th–3rd c. BCE	Alagail 2	Ala_1	this study	C4a1+16129	ABC		
	Russian Altai (Chuya)	Pazyryk	4th–3rd c. BCE	Alagail 2	Ala_2	this study	C4	ABC		
	Russian Altai (Chuya)	Pazyryk	4th–3rd c. BCE	Alagail 2	Ala_4*	this study	T	ABC		
	Russian Altai (Chuya)	Pazyryk	4th–3rd c. BCE	Barburgazy 1	B1_1*	this study	G	ABC		
	Russian Altai (Chuya)	Pazyryk	4th–3rd c. BCE	Barburgazy 1	B1_2*	this study	D4m2	ABC		
	Russian Altai (Chuya)	Pazyryk	4th–3rd c. BCE	Barburgazy 3	B3_1*	this study	T1a1b	ABC		
	Russian Altai (Chuya)	Pazyryk	4th–3rd c. BCE	Borotal 2	Bt_1	this study	T2b+@16296	ABC		
	Russian Altai (Chuya)	Pazyryk	4th–3rd c. BCE	Borotal 2	Bt_2*	this study	U2e2a	ABC		
	Russian Altai (Chuya)	Pazyryk	4th–3rd c. BCE	Dcholin 2	D_1*	this study	T1a1b	ABC		
	Russian Altai (Chuya)	Pazyryk	4th–3rd c. BCE	Justyd 12	J12_1	this study	T2b+@16296	ABC		
	Russian Altai (Chuya)	Pazyryk	4th–3rd c. BCE	Justyd 12	J12_3	this study	U7	ABC		
	Russian Altai (Chuya)	Pazyryk	4th–3rd c. BCE	Justyd 12	J12_6*	this study	Z	ABC		
	Russian Altai (Chuya)	Pazyryk	4th–3rd c. BCE	Justyd 12	J12_7*	this study	T2b+@16296	ABC		
	Russian Altai (Chuya)	Pazyryk	4th–3rd c. BCE	Justyd 12	J12_8*	this study	A8	ABC		
	Russian Altai (Chuya)	Pazyryk	4th–3rd c. BCE	Justyd 12	J12_9	this study	G1a1	ABC		
	Russian Altai (Chuya)	Pazyryk	4th–3rd c. BCE	Justyd 22	J22_1*	this study	T2b+@16296	ABC		
	Russian Altai (Chuya)	Pazyryk	4th–3rd c. BCE	Ulandryk 1	U1_1	this study	W4a	ABC		
	Russian Altai (Chuya)	Pazyryk	4th–3rd c. BCE	Ulandryk 1	U1_2	this study	Z1a	ABC		
	Russian Altai (Chuya)	Pazyryk	4th–3rd c. BCE	Ulandryk 2	U2_1*	this study	F2a	ABC		
	Russian Altai (Chuya)	Pazyryk	4th–3rd c. BCE	Ulandryk 2	U2_2	this study	F2a	ABC		
	Russian Altai (Chuya)	Pazyryk	4th–3rd c. BCE	Ulandryk 4	U4_1	this study	U2e2	ABC		
	Russian Altai (Chuya)	Pazyryk	4th–3rd c. BCE	Ulandryk 4	U4_4	this study	K	ABC		
	NW Mongolia	Pazyryk	4th–3rd c. BCE	Baga Turgen Gol	BTG05.T1	73	D4b1	ABC		
	NW Mongolia	Pazyryk	4th–3rd c. BCE	Baga Turgen Gol	BTG05.T2	73	K	ABC		
	NW Mongolia	Pazyryk	4th–3rd c. BCE	Baga Turgen Gol	BTG05.T8.1	73	U5a1	ABC		

Steppe	*Region*	*Culture*	*Dating*	*Site/Area*	*Lab code*	*Source*	*Haplogroup***	*Analyses*		
	NW Mongolia	Pazyryk	4th–3rd c. BCE	Baga Turgen Gol	BTG05.T8.2	73	C	ABC		
	NW Mongolia	Pazyryk	4th–3rd c. BCE	Baga Turgen Gol	BTG05.T8.3	73	D	ABC		
	NW Mongolia	Pazyryk	4th–3rd c. BCE	Baga Turgen Gol	BTG06.T3	73	J	ABC		
	NW Mongolia	Pazyryk	4th–3rd c. BCE	Baga Turgen Gol	BTG06.T8	73	D	ABC		
	NW Mongolia	Pazyryk	4th–3rd c. BCE	Baga Turgen Gol	BTG06.T10A	73	HV6	ABC		
	NW Mongolia	Pazyryk	4th–3rd c. BCE	Baga Turgen Gol	BTG06.T10B	73	D	ABC		
	NW Mongolia	Pazyryk	4th–3rd c. BCE	Baga Turgen Gol	BTG06.T11A	73	K	ABC		
	NW Mongolia	Pazyryk	4th–3rd c. BCE	Baga Turgen Gol	BTG06.T11B	73	K	ABC		
	NW Mongolia	Pazyryk	4th–3rd c. BCE	Baga Turgen Gol	BTG06.T12	73	U5a1	ABC		
	NW Mongolia	Pazyryk	4th–3rd c. BCE	Baga Turgen Gol	BTG03.T13	73	C4a1+16129	ABC		
	NW Mongolia	Pazyryk	4th–3rd c. BCE	Tsengel Khairkhan	TSK07.T1	73	A	ABC		
	NW Mongolia	Pazyryk	4th–3rd c. BCE	Tsengel Khairkhan	TSK07.T2A	73	G2a	ABC		
	NW Mongolia	Pazyryk	4th–3rd c. BCE	Tsengel Khairkhan	TSK07.T2B	73	T1a	ABC		
	NW Mongolia	Pazyryk	4th–3rd c. BCE	Olon Kurin Gol	OKG6	74	HV2	ABC		
	NW Mongolia	Pazyryk	4th–3rd c. BCE	Olon Kurin Gol	OKG10	74	U5a1	ABC		
West	North Caucasus	initial Scythian	8th–6th c. BCE	Novozavedennoe 2	NOV_5	this study	H-CRS	ABC		
	North Caucasus	initial Scythian	8th–6th c. BCE	Novozavedennoe 2	NOV_7	this study	H1c	ABC		
	North Caucasus	initial Scythian	8th–6th c. BCE	Novozavedennoe 2	NOV_9	this study	T2g1			
	North Caucasus	initial Scythian	8th–6th c. BCE	Novozavedennoe 2	NOV_10	this study	X	ABC		
	North Pontic region	classic Scythian	3rd c. BCE	Kolbino 1	KOL_1	this study	X4	ABC		
	North Pontic region	classic Scythian	3rd c. BCE	Kolbino 1	KOL_2	this study	H8c	ABC		
	North Pontic region	classic Scythian	3rd c. BCE	Kolbino 1	KOL_3	this study	U4	ABC		
	North Pontic region	classic Scythian	3rd c. BCE	Kolbino 1	KOL_5	this study	J2b1a	ABC		
	North Pontic region	classic Scythian	6th–2nd c. BCE	Rostov-Don	RD1	75	F1b	ABC		
	North Pontic region	classic Scythian	6th–2nd c. BCE	Rostov-Don	RD2	75	C	ABC		
	North Pontic region	classic Scythian	6th–2nd c. BCE	Rostov-Don	RD5	75	U5a1	ABC		
	North Pontic region	classic Scythian	6th–2nd c. BCE	Rostov-Don	RD6	75	T1a	ABC		
	North Pontic region	classic Scythian	6th–2nd c. BCE	Rostov-Don	RD7	75	T2	ABC		
	North Pontic region	classic Scythian	6th–2nd c. BCE	Rostov-Don	RD8	75	A4	ABC		
	North Pontic region	classic Scythian	6th–2nd c. BCE	Rostov-Don	RD9	75	CRS	ABC		
	North Pontic region	classic Scythian	6th–2nd c. BCE	Rostov-Don	RD10	75	H2a1	ABC		
	North Pontic region	classic Scythian	6th–2nd c. BCE	Rostov-Don	RD11	75	T1a	ABC		
	North Pontic region	classic Scythian	6th–2nd c. BCE	Rostov-Don	RD12	75	U2e	ABC		
	North Pontic region	classic Scythian	6th–2nd c. BCE	Rostov-Don	RD13	75	D	ABC		
	North Pontic region	classic Scythian	6th–2nd c. BCE	Rostov-Don	RD14	75	I3	ABC		

Steppe	*Region*	*Culture*	*Dating*	*Site/Area*	*Lab code*	*Source*	*Haplogroup***	*Analyses*		
	North Pontic region	classic Scythian	6th–2nd c. BCE	Rostov-Don	RD15	75	I3	ABC		
	North Pontic region	classic Scythian	6th–2nd c. BCE	Rostov-Don	RD16	75	U5a	ABC		
	North Pontic region	classic Scythian	6th–2nd c. BCE	Rostov-Don	RD17	75	D4b1	ABC		
	North Pontic region	Sarmatian	6th–2nd c. BCE	Rostov-Don	RD3	75	U7	ABC		
	Southern Ural	Early Sarmatian	5th–2nd c. BCE	Pokrovka	Pr1	this study	U3	ABC		
	Southern Ural	Early Sarmatian	5th–2nd c. BCE	Pokrovka	Pr3	this study	M	ABC	capture	
	Southern Ural	Early Sarmatian	5th–2nd c. BCE	Pokrovka	Pr4	this study	U1a'c	ABC		
	Southern Ural	Early Sarmatian	5th–2nd c. BCE	Pokrovka	Pr5	this study	T	ABC		
	Southern Ural	Early Sarmatian	5th–2nd c. BCE	Pokrovka	Pr6	this study	F1b	ABC		
	Southern Ural	Early Sarmatian	5th–2nd c. BCE	Pokrovka	Pr7	this study	N1a1a1a1a	ABC		
	Southern Ural	Early Sarmatian	5th–2nd c. BCE	Pokrovka	Pr8	this study	T2	ABC		
	Southern Ural	Early Sarmatian	5th–2nd c. BCE	Pokrovka	Pr9	this study	U2e2	ABC	capture	
	Southern Ural	Early Sarmatian	5th–2nd c. BCE	Pokrovka	Pr10	this study	H2a1f	ABC		
	Southern Ural	Early Sarmatian	5th–2nd c. BCE	Pokrovka	Pr11	this study	T1a	ABC		
	Southern Ural	Early Sarmatian	5th–2nd c. BCE	Pokrovka	Pr13	this study	U5a1d2b	ABC		

* These samples were independently prepared and their HVR1 sequences analysed in Novosibirsk by A. Pilipenko. The results were all consistent with the results obtained in Mainz

** for details see Supplementary Table 4–6

only SNPs were analysed (no HVR1 results)

only the HVR1 was analysed

HVR1 and some specific SNPs were analysed

Supplementary Table 2. Sample sites and DNA preservation

Sample sites are listed according to their geographical region, dating and cultural assignment. Samples are given as follows used/analysed. For the samples that were not incorporated in any analyses the reason is given in the last column with abbreviations: a = no DNA; b = poor DNA; c = uncertain dating; d = sample assignment (e.g. double sampling of one individual); e = insufficient DNA extract for validation. Overall amplification success rate is given for the different geographical regions.

region	sub-region	site	coordinates	date [c BCE]	culture	samples	reason for exclusion	amplification success
EAST	East Kazakhstan	Ismailovo	~ 50°18'N 81°26'E	9th - 7th	Zevakino-Chilikta	3		96.1%
		Zevakino	~ 50°12'N 81°49'E	9th - 7th	Zevakino-Chilikta	8/9	c	
		Majemir	~ 49°10'N 84°57'E	not known	not known	0/3	3xc	
	Tuva	Arzhan 2	~ 52°05'N 93°42'E	7th - 6th	Aldy Bel	15/24	6xc,3xd	97.3%
	Khakassia	Barsučij Log	~ 54°00'N 91°11'E	5th	Tagar	6		100%
	Altai	Berel'	~ 49°20'N 86°12'E	4th - 3rd	Pazyryk	9/10	d	98.8%
		Tar Asu	~ 49°17'N 86°19'E	4th - 3rd	Pazyryk	1		
		Ak-Alakha 1, 4, 5	~ 49°17'N 87°32'E	4th - 3rd	Pazyryk	8/11	2xb, d	
		Alagail 2	~ 50°12'N 87°42'E	4th - 3rd	Pazyryk	3		
		Balik-Sook	~ 50°48'N 86°00'E	4th - 3rd	Pazyryk	2		
		Borotal 2	~ 50°12'N 87°42'E	4th - 3rd	Pazyryk	2		
		Barburgazy 1, 3	~ 49°49'N 89°08'E	4th - 3rd	Pazyryk	3		
		Dcholin 2	~ 49°48'N 89°22'E	4th - 3rd	Pazyryk	1		
		Justyd 12, 22	~ 49°46'N 89°18'E	4th - 3rd	Pazyryk	7/8	b	
		Moinak 2	~ 49°19'N 87°35'E	4th - 3rd	Pazyryk	2		
		Kuturguntas 1	~ 49°25'N 87°35'E	4th - 3rd	Pazyryk	1/2	d	
		Ulandryk 1, 2, 4	~ 49°41'N 89°06'E	4th - 3rd	Pazyryk	6		
		Verch Kal'dzin 1	~ 49°23'N 87°34'E	4th - 3rd	Pazyryk	1		
	South Kazakhstan	Cirikrabat	~ 44°05'N 62°54'E	4th - 2nd	Sacae	0/3	2xa,b	14.3%
WEST	SW Russia	Kolbinho	~ 51°37'N 39°11'E	3rd	Scythian	4/5	b	90.2%
		Novozavedennoe 2	~ 44°16'N 43°38'E	8th	Initial Scythian	4/10	3xa,2xb,e	
		Pokrovka	~ 52°04'N 53°56'E	5th-2nd	Early Sarmatian	11/12	b	

Supplementary Table 3. Primer sequences

Given are the primer sequences for the coding region SNPs and two alternative sets to amplify the HVR1. Primer names indicate the position of the first base following the primer. T_m = melting temperature; T_{ann} = annealing temperature; L_{prod} = product length (including primer); MP = indicates in which of the three multiplex PCR setups (A, B or C) the respective marker was amplified.

Primer	Sequence 5' --> 3'	Length	T_m	T_{ann}	L_{prod}	MP
423_U	AATTTTATCTTTTGGCGGTATGCACTT	27-mer	58.6	52.8	110	A
485_L	GATGGGCGGGGGTTGTATTG	20-mer	58.5			
654_U	CTCACATCACCCCATAAACAAATAGG	26-mer	56.8	51.3	94	B
699_L	AACTCACTGGAACGGGGATGCT	22-mer	58.5			
2992_U	CAACAATAGGGTTTACGACCTCGAT	25-mer	56.6	53.6	116	C
3057_L	CTCCGGTCTGAACTCAGATCACGTA	25-mer	58.4			
4155_U	TACCCCCGATTCCGCTACGA	20-mer	58.9	52.9	113	A
4221_L	ATGCTGGAGATTGTAATGGGTATGGA	26-mer	58.7			
4499_U	CTGGCCCAACCCGTCATCTA	20-mer	57.0	53.5	103	B
4554_L	GCATGTTTATTTCTAGGCCTACTCAGG	27-mer	57.1			
4549_U	ACAGCGCTAAGCTCGCACTGAT	22-mer	58.0	51.9	113	C
4617_L	ATGGCAGCTTCTGTGGAACGAG	22-mer	58.0			
4815_U	GAATAGCCCCCTTTCACTTCTGAGTC	26-mer	58.7	55.3	101	A
4864_L	TGAGATGGGGGCTAGTTTTTGTCAT	25-mer	58.9			
4871_U	GGCCTGCTTCTTCTCACATGACA	23-mer	58.0	53.4	120	B
4940_L	ACTGCCTGCTATGATGGATAAGATTGA	27-mer	58.3			
5163_U	CCAGCACCACGACCCTACTACTATCT	26-mer	58.0	51.5	71	C
5179_L	GGATGGAATTAAGGGTGTTAGTCATGTT	28-mer	58.0			
5836_U	AAATCACCTCGGAGCTGGTAAAAAG	25-mer	58.0	52.2	88	A
5875_L	GGGGTGAGGTAAAATGGCTGAGT	23-mer	57.2			
6336_U	CACCCTGGAGCCTCCGTAGAC	21-mer	57.3	53.5	116	B
6403_L	ATGGCAGGGGGTTTTATATTGATAATT	27-mer	57.5			
6764_U	CAATTGGCTTCCTAGGGTTTATCGT	25-mer	57.8	51.8	101	C
6814_L	GATGATTATGGTAGCGGAGGTGAAA	25-mer	57.4			
6975_U	GGTGGCCTGACTGGCATTGTA	21-mer	57.1	53.1	120	A
7046_L	TATGATGGCAAATACAGCTCCTATTGA	27-mer	57.2			
8226_U	CATGCCCATCGTCCTAGAATTAA	23-mer	54.9	52.6	112	B
8287_L	GCTAAGTTAGCTTTACAGTGGGCTCTA	27-mer	55.4			
8385_U	TACAGTGAAATGCCCCAACTAAATACTA	28-mer	55.9	50.7	89	C
8417_L	TTTAGTTGGGTGATGAGGAATAGTGTAA	28-mer	55.7			
8932_U	ACTTCTTACCACAAGGCACACCTACA	26-mer	57.2	53.4	116	A
8996_L	AGTAATGTTAGCGGTTAGGCGTACG	25-mer	57.0			
9072_U	GAAGCGCCACCCTAGCAATATC	22-mer	56.7	51.3	101	B
9124_L	TAAGGCGACAGCGATTTCTAGGATAG	26-mer	58.3			
10000_U	CATCTATTGATGAGGGTCTTACTCTTTTA	29-mer	54.3	47.2	107	C
10048_L	AAATTAAGGCGAAGTTTATTACTCTTTTT	29-mer	54.3			
10105_U	TTAATAATCAACACCCTCCTAGCCTTAC	28-mer	56.0	51.1	109	A
10166_L	GGTCGAAGCCGCACTCGTA	19-mer	56.1			
10387_U	TCTGGCCTATGAGTGACTACAAAAAG	26-mer	55.1	48.8	117	B
10451_L	AGGGGCATTTGGTAAATATGATTATC	26-mer	55.1			

Primer	Sequence 5' --> 3'	Length	T_m	T_{ann}	L_{prod}	MP
10865_U	CAACCACCCACAGCCTAATTATTAGC	26mer	58.2	49.9	83	C
10895_L	TGGGGAACAGCTAAATAGGTTGTTGT	26mer	58.2			
11700_U	AGCTTCACCGGCGCAGTCA	19-mer	59.0	53.2	89	A
11743_L	GTGCGTTCGTAGTTTGAGTTTGCTAG	26-mer	57.6			
11935_U	ACCACGTTCTCCTGATCAAATATCAC	26-mer	56.5	51.6	101	B
11983_L	CCCCATTGTGTTGTGGTAAATATGTA	26-mer	55.9			
12303_U	GATAACAGCTATCCATTGGTCTTAGGC	27-mer	58.2	51.1	103	C
12352_L	GGAAGTCAGGGTTAGGGTGGTTATAG	26-mer	58.0			
12692_U	CAGACCCAAACATTAATCAGTTCTTCA	27-mer	56.8	51.0	110	A
12754_L	GCCCTCTCAGCCGATGAACA	20-mer	57.4			
13231_U	GCGCCCTTACACAAAATGACATC	23-mer	57.3	51.1	94	B
13275_L	GGTTGGTTGATGCCGATTGTAACTAT	26-mer	58.3			
13620_U	AAGCGCCTATAGCACTCGAATAATTCT	27-mer	58.3	53.8	116	C
13683_L	CCAGGCGTTTAATGGGGTTTAGTAG	25-mer	58.2			
13701_U	ACCCCACCCTACTAAACCCCATTAA	25-mer	58.9	53.7	88	A
13740_L	GATGCGGGGGAAATGTTGTTAGT	23-mer	57.9			
14717_U	CAACCACGACCAATGATATGAAAAAC	26-mer	57.5	51.4	120	B
14784_L	GGAGGTCGATGAATGAGTGGTTAATT	26-mer	57.6			
14783_U	ATACGCAAAACTAACCCCCTAATAAAA	27-mer	56.3	52.5	105	C
14839_L	GCCAAGGAGTGAGCCGAAGTT	21-mer	57.1			

HVR1 Primer

Primer	Sequence 5' --> 3'	Length	T_m	T_{Ann}	L_{Prod}	MP
16011_U	AGCACCCAAAGCTAAGATTCTAATTT	26-mer	54.7	51.9	130	A
16088_L	GTGGCTGGCAGTAATGTACGAAATAC	26-mer	57.1			
16071_U	GGGTACCACCCAAGTATTGACTCA	24-mer	55.8	52.2	134	B
16153_L	TGATGTGGATTGGGTTTTTATGTACTA	27-mer	55.0			
16119_U	GTACATTACTGCCAGCCACCATG	23-mer	55.9	52.5	138	C
16207_L	TGATAGTTGAGGGTTGATTGCTGTAC	26-mer	55.5			
16185_U	TACATAAAAACCCAATCCACATCAAAAC	28-mer	57.6	54.1	139	A
16271_L	GGTGGGTAGGTTTGTTGGTATCCT	24-mer	56.2			
16233_U	AGTACAGCAATCAACCCTCAACTATC	26-mer	54.1	52.4	127	B
16305_L	TGTACGGTAAATGGCTTTATGTACTATG	28-mer	54.4			
16274_U	AAAGCCACCCCTCACCCACTAG	22-mer	58.1	53.5	116	C
16345_L	TGGGGACGAGAAGGGATTTGAC	22-mer	58.8			
16340_U	ACATAAAGCCATTTACCGTACATAGCAC	28-mer	57.2	54.7	127	A
16413_L	CACTCTTGTGCGGGATATTGATTTC	25-mer	57.8			

Alternative HVR1 Primer

Primer	Sequence 5' --> 3'	Length	T_m	T_{Ann}	L_{Prod}	MP
16013_U	AGCACCCAAAGCTAAGATTCTAATTTAA	28-mer	56.0	52.9	196	A
16152_L	TGATGTGGATTGGGTTTTTATGTACTAC	28-mer	55.8			
16122_U	ATTACTGCCAGCCACCATGAAT	22-mer	55.0	54.3	196	B
16271_L	GGTGGGTAGGTTTGTTGGTATCCT	24-mer	56.2			
16235_U	AAGTACAGCAATCAACCCTCAACTATCAC	29-mer	58.3	54.5	163	C
16346_L	ATGGGGACGAGAAGGGATTTGA	22-mer	58.3			
16274_U	AAAGCCACCCCTCACCCACTAG	22-mer	58.1	55.9	182	A
16410_L	TTGTGCGGGATATTGATTTCACG	23-mer	58.4			

Supplementary Table 4. Sample haplogroups

Haplogroups (HGs) were determined using the online version of Haplogrep, based on the Phylotree rCRS built14; the ranking acts in accordance to the number of SNPs that could be assigned to the respective HG, mutations that have not been found yet or are missing for the most likely HG. Equally likely HGs are given in brackets behind the root of those, or the most conservative one was chosen. HG determination was based on coding region SNPs and the HVR1 (position 16040–16400) except for the following: NOV_9: HG based only on coding region SNPs; Pr4–6, 8, 9, 11 and 13: HG based only on HVR1 (for SNP details and HVR1 profiles see Supplementary Table 5 and 6). Ind. = Individual.

Site	Kurgan	grave	Ind.	Lab code	Haplogroup	Ranking
Arzhan 2	2	M5	1	A_2	A8	100.0
	2	M5	2	A_3	G	100.0
	2	M7		A_4	D4b1a2a	100.0
	2	M8		A_5	C5b1	100.0
	2	M12		A_6	U5a1f1	100.0
	2	M13a	1	A_7	Y1	93.7
	2	M13a	2	A_8	U4a3	92.0
	2	M13b		A_9	U5a1d2b	100.0
	2	M14	1	A_10	A11 (A8)	88.6 (88.0)
	2	M14	2	A_11	G	100.0
	2	M20	1	A_14	T1a	100.0
	2	M22		A_17	H (H1+16189)	(64.0)
	2	M24		A_19	C4	96.2
	2	M25		A_20	G2a	96.1
	2	M26		A_21	A4	95.7
Ak Alakha 1	1	1		Ak1_1	C4a1+16129	100.0
Ak Alakha 4	1			Ak4_1	A11 (A8)	91.7 (91.0)
Ak Alakha 5	1			Ak5_1	C4	98.8
	3	1		Ak5_4	D4b1	93.5
	3	2		Ak5_5	U2e1a	94.7
	4	1		Ak5_6	U2e1a	94.7
	4	2		Ak5_7	C4a1+16129	100.0
	5			Ak5_8	A11 (A8)	91.7 (91.0)
Alagail 2	8	12		Ala_1	C4a1+16129	100.0
	8	15		Ala_2	C4	95.6
	10			Ala_4	T	93.6
Barburgazy 1	4 (A)			B1_1	G	100.0
	7		1	B1_2	D4m2	97.9
Barburgazy 3	5		2	B3_1	T1a1b	100.0
Berel'	16			Be_2	D4	96.0
	16			Be_3	H-CRS	
	32			Be_4	A11 (A8)	91.7 (91.0)
	10			Be_6	A6	95.7

**Site**	**Kurgan**	**grave**	**Ind.**	**Lab code**	**Haplogroup**	**Ranking**
	34			Be_8	D4	100.0
	73			Be_9	A4f	100.0
	31			Be_11	C4a1+16129	100.0
	36			Be_12	HV2	100.0
	71			Be_14	A6	95.7
Barsucij Log		1	1	BL_1	C5+16093	100.0
		1	2	BL_2	U5a1a2 (U5a2c3)	91.3 (91.0)
		1	3	BL_3	C5+16093	100.0
		1	4	BL_4	C5+16093	100.0
		1	5	BL_5	U2e2	100.0
		2	1	BL_6	A4	98.6
Balik Sook	1			BS_1	X2b	100.0
	27			BS_2	U4b1a4	100.0
Borotal 2	2			Bt_1	T2b+@16296	100.0
	3		2	Bt_2	U2e2a	100.0
Dcholin 2	1			D_1	T1a1b	100.0
Ismailovo	6			Is_1	D4h1	100.0
	10		1	Is_2	HV-CRS	100.0
	27			Is_4	H2a1	100.0
Justyd 12	1			J12_1	T2b+@16296	100.0
	6			J12_3	U7	91.8
	10			J12_6	Z	100.0
	17		2	J12_7	T2b+@16296	100.0
	17		3	J12_8	A8	97.9
	18			J12_9	G1a1	97.0
Justyd 22	1			J22_1	T2b+@16296	100.0
Kuturguntas 1	1			K_1	H-CRS	
Kolbino 1	8	1		KOL_1	X4	100.0
	10			KOL_2	H8c	100.0
	10_I			KOL_3	U4	100.0
	44	4		KOL_5	J2b1a	100.0
Moinak 2	1		1	Mo_1	D4b1a2a1	97.9
	2		1	Mo_2	D4h4a	97.4
Novozavedennoe 2	3	1		NOV_5	H-CRS	
	2			NOV_7	H1c	84.7
	12			NOV_9	T2g1	100.0
	14			NOV_10	X	92.9
Pokrovka	2			Pr1	U3	83.4
	2			Pr3	M (M5/M7b'd/M13b)	each 100.0
	2			Pr4	U1a'c	100.0
	2			Pr5	T	100.0
	2			Pr6	F1b	90.9
	2			Pr7	N1a1a1a1a	72.3
	2			Pr8	T2	100.0
	2			Pr9	U2e2	100.0
	2			Pr10	H2a1f	81.8

Site	Kurgan	grave	Ind.	Lab code	Haplogroup	Ranking
	2			Pr11	T1a	100.0
	2			Pr13	U5a1d2b	96.6
Tar Asu	23			Ta_1	K2b1a	100.0
Ulandryk 1	9			U1_1	W4a	91.1
	11			U1_2	Z1a	98.1
Ulandryk 2	1			U2_1	F2a	92.5
	2			U2_2	F2a	92.5
Ulandryk 4	2			U4_1	U2e2	98.5
	3			U4_4	K	100.0
Verch Kal'dzin 1	1			VK1_1	K1 (K1b2/K1c2)	89.7 (96.5/93.3)
Zevakino	99b			Ze_2	T2b	100.0
	224			Ze_3	K1a1 (K1b1b)	je 100.0
	10	1		Ze_4	D4h4a (D4j+16311)	je 96.1
	10	2		Ze_5	I	100.0
	46	1		Ze_6	C4 (C4a1/C4b8/C4+152+16093)	98,6 (each 100)
	33			Ze_7	U4a1	100.0
	10	4		Ze_8	D4	100.0
	336			Ze_9	D4j3	97.3

Supplementary Table 5. HVR1 profiles

Mitochondrial HVR1 profiles of the 97 samples used for analyses. There was not enough material to reproduce the HVR1 profile of sample NOV_9 so it remains not determined (nd). For sample Pr4 the Y at position 16248 was reproduced in seven PCRs from two independent extractions.

Site	Lab code	HVR1 Polymorphisms					
Arzhan 2	A_2	16223T	16242T	16290T	16319A		
	A_3	16223T	16362C				
	A_4	16223T	16319A	16362C			
	A_5	16148T	16223T	16288C	16298C	16327T	
	A_6	16192T	16256T	16270T	16311C	16399G	
	A_7	16126C	16231C	16266T			
	A_8	16327A	16356C	16362C			
	A_9	16192T	16256T	16270T	16304C	16399G	
	A_10	16224C	16242T	16290T	16293C	16319A	
	A_11	16223T	16362C				
	A_14	16126C	16163G	16186T	16189C	16294T	
	A_17	16189C					
	A_19	16111A	16223T	16298C	16327T		
	A_20	16223T	16227G	16262T	16278T	16362C	
	A_21	16223T	16286T	16290T	16319A	16362C	
Ak Alakha 1	Ak1_1	16093C	16129A	16223T	16298C	16327T	
Ak Alakha 4	Ak4_1	16242T	16290T	16293C	16319A		
Ak Alakha 5	Ak5_1	16129A	16223T	16298C	16327T		
	Ak5_4	16223T	16239T	16319A	16362C		
	Ak5_5	16051G	16129C	16182C	16183C	16189C	16362C
	Ak5_6	16051G	16129C	16182C	16183C	16189C	16362C
	Ak5_7	16093C	16129A	16223T	16298C	16327T	
	Ak5_8	16242T	16290T	16293C	16319A		
Alagail 2	Ala_1	16093C	16129A	16223T	16298C	16327T	
	Ala_2	16086C	16223T	16287T	16298C	16327T	
	Ala_4	16126C	16189C	16292T	16294T		
Barburgazy 1	B1_1	16223T	16362C				
	B1_2	16042A	16223T	16243C	16362C		
Barburgazy 3	B3_1	16126C	16163G	16186T	16189C	16294T	
Berel'	Be_2	16223T	16362C				
	Be_3	rCRS					
	Be_4	16242T	16290T	16293C	16319A		
	Be_6	16223T	16290T	16319A	16362C		
	Be_8	16223T	16362C				
	Be_9	16223T	16290T	16292A	16319A	16362C	

Site	Lab code	HVR1 Polymorphisms						
	Be_11	16093C	16129A	16223T	16298C	16327T		
	Be_12	16217C						
	Be_14	16223T	16290T	16319A	16362C			
Barsucij Log	BL_1	16093C	16223T	16288C	16298C	16327T		
	BL_2	16256T	16270T	16309G				
	BL_3	16093C	16223T	16288C	16298C	16327T		
	BL_4	16093C	16223T	16288C	16298C	16327T		
	BL_5	16051G	16092C	16129C	16183C	16189C	16362C	
	BL_6	16189C	16223T	16290T	16319A	16362C		
Balik Sook	BS_1	16189C	16223T	16278T				
	BS_2	16356C						
Borotal 2	Bt_1	16126C	16294T	16304C				
	Bt_2	16051G	16092C	16129C	16183C	16189C	16362C	
Dcholin 2	D_1	16126C	16163G	16186T	16189C	16294T		
Ismailovo	Is_1	16174T	16223T	16362C				
	Is_2	rCRS						
	Is_4	16354T						
Justyd 12	J12_1	16126C	16294T	16304C				
	J12_3	16093C	16318T					
	J12_6	16185T	16223T	16260T	16298C			
	J12_7	16126C	16294T	16304C				
	J12_8	16223T	16242T	16278T	16290T	16319A		
	J12_9	16223T	16263C	16325C	16362C			
Justyd 22	J22_1	16126C	16294T	16304C				
Kuturguntas 1	K_1							
Kolbino 1	KOL_1	16183C	16189C	16223T	16266T	16274A	16278T	16390A
	KOL_2	16288C	16362C					
	KOL_3	16356C						
	KOL_5	16069T	16126C	16193T	16278T			
Moinak 2	Mo_1	16093C	16172C	16173T	16223T	16319A	16362C	
	Mo_2	16223T	16311C	16316G	16362C			
Novozavedennoe 2	NOV_5	rCRS						
	NOV_7	16136C						
	NOV_9	nd						
	NOV_10	16129C	16189C	16223T	16278T			
Pokrovka	Pr1	16188T	16327T	16343G				
	Pr3	16129A	16223T					
	Pr4	16182C	16183C	16189C	16248Y	16249C		
	Pr5	16126C	16294T					
	Pr6	16183C	16189C	16232A	16249C	16304C	16311C	16399G
	Pr7	16147A	16172C	16189C	16223T	16248T	16320T	16355T
	Pr8	16126C	16294T	16296T				

Site	Lab code	HVR1 Polymorphisms						
	Pr9	16051G	16092C	16129C	16183C	16189C	16362C	
	Pr10	16193T	16264T	16354T				
	Pr11	16126C	16163G	16186T	16189C	16294T		
	Pr13	16192T	16256T	16270T	16304C	16311C	16399G	
Tar Asu	Ta_1	16224C	16270T	16311C				
Ulandryk 1	U1_1	16188T	16189C	16223T	16286T			
	U1_2	16129A	16185T	16223T	16224C	16260T	16294T	16298C
Ulandryk 2	U2_1	16093C	16203G	16231C	16291T	16304C		
	U2_2	16093C	16203G	16231C	16291T	16304C		
Ulandryk 4	U4_1	16051G	16092C	16129C	16182C	16183C	16189C	16362A
	U4_4	16224C	16311C					
Verch Kal'dzin 1	VK1_1	16224C	16311C	16320T				
Zevakino	Ze_2	16126C	16294T	16296T	16304C			
	Ze_3	16093C	16224C	16311C				
	Ze_4	16171G	16223T	16311C	16362C			
	Ze_5	16129A	16223T	16391A				
	Ze_6	16093C	16223T	16298C	16327T			
	Ze_7	16134T	16356C					
	Ze_8	16223T	16362C					
	Ze_9	16184T	16223T	16265C	16311C	16362C		

Supplementary Table 6. Mitochondrial coding region SNPs

M01–M30 refer to the amplified fragments; SNP gives the SNP position according to the rCRS; grey indicates additional polymorphic sites besides the target SNP; ref = reference base according to the rCRS; - = no SNP; x = site missing

Site	Lab code		M01		M02			M03		M04		M05		M06	M07		M08			M09		M10	M11			M12		M13	
		SNP	456	477	654	663	689	3010	3027	4173	4216	4529	4553	4580	4824	4833	4883	4917	4924	5171	5178	5843	6340	6371	6392	6776	6779	6975	7028
		ref	C	T	T	A	T	G	T	A	T	A	T	G	A	A	C	A	G	A	C	A	C	C	T	T	A	T	C
Arzhan 2	A_2		-	-	-	G	-	-		-	-	-	-	-	G	-	-	-		-	-	-	-	-	-	-	-	-	T
	A_3		-	-	-	-	-	-	-	-	-	-	-	-	-	G	-	-	-	-	-	-	-	-	-	-	-	-	T
	A_4		-	-	-	-	-	A	-	-	-	-	-	-	-	-	T	-	-	-	A	-	-	-	-	-	-	-	T
	A_5		-	-	-	-	-	-	-	-	-	-	-	-	-	-	-	-	-	-	-	-	-	-	-	-	-	-	T
	A_6		-	-	-	-	-	-	-	-	-	-	-	-	-	-	-	-	-	-	-	-	-	-	-	-	-	-	T
	A_7		-	-	-	-	-	-	-	-	-	-	-	-	-	-	-	-	-	-	-	-	-	-	-	-	-	-	T
	A_8		-	-	-	-	-	-	-	-	-	-	-	-	-	-	-	-	-	-	-	-	-	-	-	-	-	-	T
	A_9		-	-	-	-	-	-	C	-	-	-	-	-	-	-	-	-	C	-	-	-	-	-	-	-	-	-	T
	A_10		-	-	-	G	-	-	-	-	-	-	-	-	-	G	-	-	-	-	-	-	-	-	-	-	G	-	T
	A_11		-	-	-	-	-	-	-	-	-	-	-	-	-	G	-	-	-	-	-	-	-	-	-	-	-	-	T
	A_14		-	-	-	-	-	-	-	-	C	-	-	-	-	-	-	G	-	-	-	-	-	-	-	-	-	-	T
	A_17		-	-	-	-	-	-	-	-	-	-	-	-	-	-	-	-	-	-	-	-	-	-	-	-	-	-	-
	A_19		-	-	-	-	-	-	-	-	-	-	-	-	-	-	-	-	-	G	-	-	-	-	-	-	-	-	T
	A_20		-	-	-	-	-	-	-	-	-	-	-	-	-	G	-	-	-	-	-	-	T	-	-	-	-	-	T
	A_21		-	-	-	G	-	-	-	-	-	-	-	-	G	-	-	-	-	-	-	-	-	-	-	-	-	-	T
Ak Alakha 1	Ak1_1*		-	-	-	-	-	-	-	-	-	-	-	-	-	-	-	-	-	-	-	-	-	-	-	-	-	-	T
Ak Alakha 4	Ak4_1		-	-	-	G	-	-	-	-	-	-	-	-	G	-	-	-	-	-	-	-	-	-	-	-	G	-	T
Ak Alakha 5	Ak5_1*		-	-	-	-	-	-	-	-	-	-	-	-	-	-	-	-	-	-	-	-	-	-	-	-	-	-	T
	Ak5_4*		-	-	-	-	-	A	-	-	-	-	-	-	-	-	T	-	-	-	A	-	-	-	-	-	-	-	T
	Ak5_5*		-	-	-	-	-	-	-	-	-	-	-	-	-	-	-	-	-	-	-	-	-	-	-	-	-	-	T
	Ak5_6*		-	-	-	-	-	-	-	-	-	-	-	-	-	-	-	-	-	-	-	-	-	-	-	-	-	-	T
	Ak5_7*		-	-	-	-	-	-	-	-	-	-	-	-	-	-	-	-	-	-	-	-	-	-	-	-	-	-	T
	Ak5_8*		-	-	-	G	-	-	-	-	-	-	-	-	G	-	-	-	-	-	-	-	-	-	-	-	G	-	T
Alagail 2	Ala_1		-	-	-	-	-	-	-	-	-	-	-	-	-	-	-	-	-	-	-	-	-	-	-	-	-	-	T
Alagail 2	Ala_2		-	-	-	-	-	-	-	-	-	-	-	-	-	-	-	-	-	-	-	-	-	-	-	-	-	-	T
Alagail 2	Ala_4*		-	-	-	-	-	-	-	-	C	-	-	-	-	-	-	G	-	-	-	-	-	-	-	-	-	-	T
Barburgazy 1	B1_1*		-	-	-	-	-	-	-	-	-	-	-	-	-	G	-	-	-	-	-	-	-	-	-	-	-	-	T
	B1_2*		-	-	-	-	-	A	-	-	-	-	-	-	-	-	T	-	-	-	A	-	-	-	-	-	-	-	T
Barburgazy 3	B3_1*		-	-	-	-	-	-	-	-	C	-	-	-	-	-	-	G	-	-	-	-	-	-	-	-	-	-	T

Site	Lab code		M14			M15			M16			M17	M18	M19			M20		M21		M22		M23			M24			M25			M26	M27	M28				M29		M30			
		SNP	8251	8273	9bp del	8392	8393	8414	8962	8989	8994	9090	10034	10118	10142	10143	10398	10400	10873	10876	11719	11732	11947	11965	11969	12308	12338	12346	12705	12732	12738	13263	13626	13708	13711	13720	13734	14766	14783	14793	14798	14815	14839
		ref	G	C	-	G	C	C	A	G	G	T	T	T	C	G	A	C	T	A	G	T	A	C	G	A	T	C	C	T	T	A	C	G	G	C	T	C	T	A	T	C	A
Arzhan 2	A_2		-	-	-	-	-	-	-	-	-	-	x	-	-	-	-	-	-	-	A	-	-	-	-	-	-	-	T	-	-	-	-	-	-	-	-	T	-	-	-	-	-
	A_3		-	-	-	-	-	-	-	-	-	-	x	-	-	-	G	T	C	-	A	-	-	-	-	-	-	-	T	-	-	-	-	-	-	-	-	x	C	-	-	-	-
	A_4		-	-	-	-	-	T	-	-	-	-	x	-	-	-	G	T	C	-	A	-	-	-	-	-	-	-	T	-	-	-	-	-	-	T	-	T	C	-	-	T	-
	A_5		-	-	-	-	-	-	-	-	-	-	x	-	-	-	G	T	C	-	A	-	-	-	-	-	-	-	T	-	-	G	-	-	-	-	-	T	C	-	-	-	-
	A_6		-	-	-	-	-	-	-	-	-	-	x	-	-	-	-	-	-	-	A	-	-	-	-	G	-	-	-	-	-	-	-	-	-	-	-	T	-	G	-	-	-
	A_7		-	-	-	A	-	-	-	-	-	-	x	-	-	-	G	-	-	-	A	-	-	-	-	-	-	-	T	C	-	-	-	-	-	-	-	T	-	-	-	-	-
	A_8		-	-	-	-	-	-	-	-	-	-	x	-	-	-	-	-	-	-	A	-	-	-	-	G	-	-	-	-	-	-	-	-	-	-	-	T	-	-	-	-	-
	A_9		-	-	-	-	-	-	-	-	-	-	x	-	-	-	-	-	-	-	A	-	-	-	-	G	-	-	-	-	-	-	-	-	-	-	-	T	-	G	-	-	-
	A_10		-	-	-	-	-	-	-	-	-	-	x	-	-	-	-	-	-	-	A	-	-	-	-	-	-	-	T	-	-	-	-	-	-	-	-	T	-	-	-	-	-
	A_11		-	-	-	-	-	-	-	-	-	-	x	-	-	-	G	T	C	-	A	-	-	-	-	-	-	-	T	-	-	-	-	-	-	-	-	T	C	-	-	-	-
	A_14		-	-	-	-	-	-	-	-	-	-	x	-	-	-	-	-	-	-	A	-	-	-	-	-	-	-	-	-	-	-	-	-	-	-	-	T	-	-	-	-	-
	A_17		-	-	-	-	-	-	-	-	-	-	x	-	-	-	-	-	-	-	-	-	-	-	-	-	-	-	-	-	-	-	-	-	-	-	-	-	-	-	-	-	-
	A_19		-	-	-	-	-	-	-	-	-	-	x	-	-	-	G	T	C	-	A	-	-	-	A	-	-	-	T	-	-	G	-	-	-	-	-	T	C	-	-	-	-
	A_20		-	-	-	-	-	-	-	-	-	-	x	-	-	-	G	T	C	-	A		-	-	-	-	-	-	T	-	-	-	-	-	-	-	-	T	C	-	-	-	-
	A_21		-	-	-	-	-	-	-	-	-	-	x	-	-	-	-	-	-	-	A	-	-	-	-	-	-	-	T	-	-	-	-	-	-	-	-	T	-	-	-	-	-
Ak Alakha 1	Ak1_1*		-	-	-	-	-	-	-	-	-	-	x	-	-	-	G	T	C	-	A	-	-	-	A	-	-	-	T	-	-	G	-	-	-	-	-	T	C	-	-	-	-
Ak Alakha 4	Ak4_1		-	-	-	-	-	-	-	-	-	-	x	-	-	-	-	-	-	-	A	-	-	-	-	-	-	-	T	-	-	-	-	-	-	-	-	T	-	-	-	-	-
Ak Alakha 5	Ak5_1*		-	-	-	-	-	-	-	-	-	-	x	-	-	-	G	T	C	-	A	-	-	-	A	-	-	-	T	-	-	G	-	-	-	-	-	T	C	-	-	-	-
	Ak5_4*		-	-	-	-	-	T	-	-	-	-	x	-	-	-	G	T	C	-	A	-	-	-	-	-	-	-	-	-	-	-	-	-	-	-	-	T	C	-	-	-	-
	Ak5_5*		-	-	-	-	-	-	G	-	-	-	x	-	-	-	-	-	-	G	A	C	-	-	-	G	-	-	-	-	-	-	-	-	-	-	C	T	-	-	-	-	-
	Ak5_6*		-	-	-	-	-	-	G	-	-	-	x	-	-	-	-	-	-	G	A	C	-	-	-	G	-	-	-	-	-	-	-	-	-	-	C	T	-	-	-	-	-
	Ak5_7*		-	-	-	-	-	-	-	-	-	-	x	-	-	-	G	T	C	-	A	-	-	-	A	-	-	-	T	-	-	G	-	-	-	-	-	T	C	-	-	-	-
	Ak5_8*		-	-	-	-	-	-	-	-	-	-	x	-	-	-	-	-	-	-	A	-	-	-	-	-	-	-	T	-	-	-	-	-	-	-	-	T	-	-	-	-	-
Alagail 2	Ala_1		-	-	-	-	-	-	-	-	-	-	x	-	-	-	G	T	C	-	A	-	-	-	A	-	-	-	T	-	-	G	-	-	-	-	-	T	C	-	-	-	-
Alagail 2	Ala_2		-	-	-	-	-	-	-	-	-	-	x	-	-	-	G	T	C	-	A	-	-	-	A	-	-	-	T	-	-	G	-	-	-	-	-	T	C	-	-	-	-
Alagail 2	Ala_4*		-	-	-	-	-	-	-	-	-	-	x	-	-	-	-	-	-	-	A	-	-	-	-	-	-	-	-	-	-	-	-	-	-	-	-	T	-	-	-	-	-
Barburgazy 1	B1_1*		-	-	-	-	-	-	-	-	-	-	x	-	-	r	G	T	C	-	A	-	-	-	-	-	-	-	T	-	-	-	-	-	-	-	-	T	C	-	-	-	-
	B1_2*		-	-	-	-	-	T	-	A	-	-	x	-	-	-	G	T	C	-	A	-	-	-	-	-	-	-	T	-	-	-	-	-	-	-	-	T	C	-	-	-	-
Barburgazy 3	B3_1*		-	-	-	-	-	-	-	-	-	-	x	-	-	A	-	-	-	-	A	-	-	-	-	-	-	-	-	-	-	-	-	-	-	-	-	T	-	-	-	-	-

			M01		M02			M03		M04		M05		M06	M07		M08			M09		M10	M11			M12		M13		M14			M15			M16			M17	M18	M19			M20		M21		M22		M23			M24			M25			M26	M27	M28				M29	M30				
		SNP	456	477	654	663	689	3010	3027	4173	4216	4529	4553	4580	4824	4833	4883	4917	4924	5171	5178	5843	6340	6371	6392	6776	6779	6975	7028	8251	8273	9bp del	8392	8393	8414	8962	8989	8994	9090	10034	10118	10142	10143	10398	10400	10873	10876	11719	11732	11947	11965	11969	12308	12338	12346	12705	12732	12738	13263	13626	13708	13711	13720	13734	14766	14783	14793	14798	14815	14839
Site	Lab code	ref	C	T	T	A	T	G	T	A	T	A	T	G	A	A	C	A	G	A	C	A	C	C	T	T	A	T	C	G	C	-	G	C	C	A	G	G	T	T	T	C	G	A	C	T	A	G	T	A	C	G	A	T	C	C	T	T	A	C	G	G	C	T	C	T	A	T	C	A
Berel	Be_2		-	-	-	-	-	A	-	-	-	-	-	-	-	-	T	-	-	-	A	-	-	-	-	-	-	C	T	-	-	-	-	-	T	-	-	-	-	-	-	-	-	G	T	C	-	A	-	-	-	-	-	-	-	T	-	-	-	-	-	-	-	-	T	C	-	-	-	-
	Be_3		-	-	-	-	-	-	-	-	-	-	-	-	-	-	-	-	-	-	-	-	-	-	-	-	-	-	-	-	-	-	-	-	-	-	-	-	-	-	-	-	-	-	-	-	-	-	-	-	-	-	-	-	-	-	-	-	-	-	-	-	-	-	-	-	-	-	-	-
	Be_4		-	-	-	G	-	-	-	-	-	-	-	-	G	-	-	-	-	-	-	-	-	-	-	-	G	-	T	-	-	-	-	-	-	-	-	-	-	-	-	-	-	-	-	-	-	A	-	-	-	-	-	-	-	T	-	-	-	-	-	-	-	-	T	-	-	-	-	-
	Be_6		-		C	G	-	-	-	-	-	-	C	-	G	-	-	-	-	-	-	-	-	-	-	-	-	-	T	-	-	-	-	-	-	-	-	-	-	x	-	-	-	-	-	-	-	A	-	-	-	-	-	-	-	T	-	-	-	-	-	-	-	-	T	-	-	-	-	-
	Be_8		x	x	-	-	-	A	-	-	-	-	-	-	-	-	T	-	-	-	A	-	-	-	-	-	-	-	T	-	T	-	-	-	T	-	-	-	-	x	-	-	-	G	T	C	-	A	-	-	-	-	-	-	-	T	-	-	-	-	-	-	-	-	T	C	-	-	-	-
	Be_9		-	-	-	G	-	-	-	-	-	-	-	-	G	-	-	-	-	-	-	-	-	-	-	-	-	-	T	-	-	-	-	-	-	-	-	-	-	-	-	-	-	-	-	-	-	A	-	-	-	-	-	-	-	T	-	-	-	-	-	-	-	-	T	-	-	-	-	-
	Be_11		-	-	-	-	-	-	-	-	-	-	-	-	-	-	-	-	-	-	-	-	-	-	-	-	-	-	T	-	-	-	-	-	-	-	-	-	-	-	-	-	-	G	T	C	-	A	-	-	-	A	-	-	-	T	-	-	G	-	-	-	-	-	T	C	-	-	-	-
	Be_12		-	-	-	-	-	-	-	-	-	-	-	-	-	-	-	-	-	-	-	-	-	-	-	-	-	-	T	-	-	-	-	-	-	-	-	-	-	-	-	-	-	-	-	-	-	-	-	-	-	-	-	-	-	-	-	-	-	-	-	-	-	-	-	-	-	-	-	-
	Be_14		-	-	C	G	-	-	-	-	-	-	C	-	G	-	-	-	-	-	-	-	-	-	-	-	-	-	T	-	-	-	-	-	-	-	-	-	-	-	-	-	-	-	-	-	-	A	-	-	-	-	-	-	-	T	-	-	-	-	-	-	-	-	T	-	-	-	-	-
Barsucij Log	BL_1		-	-	-	-	-	-	-	G	-	-	-	-	-	-	-	-	-	-	-	-	-	-	-	-	-	-	T	-	-	-	-	-	-	-	-	-	-	x	-	-	-	G	T	C	-	A	-	-	-	-	-	-	-	T	-	-	G	-	-	-	-	-	T	C	-	-	-	-
	BL_2		-	-	-	-	-	-	-	-	-	-	-	-	-	-	-	-	-	-	-	-	-	-	-	-	-	-	T	-	-	-	-	-	-	-	-	-	-	x	-	-	-	-	-	-	-	A	-	-	-	-	G	-	T	-	-	-	-	-	-	-	-	-	T	-	G	-	-	-
	BL_3		-	-	-	-	-	-	-	G	-	-	-	-	-	-	-	-	-	-	-	-	-	-	-	-	-	-	T	-	-	-	-	-	-	-	-	-	-	x	-	-	-	G	T	C	-	A	-	-	-	-	-	-	-	T	-	-	G	-	-	-	-	-	T	C	-	-	-	-
	BL_4		-	-	-	-	-	-	-	G	-	-	-	-	-	-	-	-	-	-	-	-	-	-	-	-	-	-	T	-	-	-	-	-	-	-	-	-	-	x	-	-	-	G	T	C	-	A	-	-	-	-	-	-	-	T	-	-	G	-	-	-	-	-	T	C	-	-	-	-
	BL_5		-	-	-	-	-	-	-	-	-	-	-	-	-	-	-	-	-	-	-	-	-	-	-	-	-	-	T	-	-	-	x	x	-	-	-	-	-	x	-	-	-	-	-	-	G	A	-	-	-	-	G	-	-	-	-	-	-	-	-	-	-	C	T	-	-	-	-	-
	BL_6		-	-	-	G	-	-	-	-	-	-	-	-	G	-	-	-	-	-	-	-	-	-	-	-	-	-	T	-	-	-	-	-	-	-	-	-	-	x	-	-	-	-	-	-	-	A	-	-	-	-	-	-	-	T	-	-	-	-	-	-	-	-	T	-	-	-	-	-
Balik Sook	BS_1		-	-	-	-	-	-	-	-	-	-	-	-	-	-	-	-	-	-	-	-	-	T	-	-	-	-	T	-	-	-	-	T	-	-	-	-	-	x	-	-	-	-	-	-	-	A	-	-	-	-	-	-	-	T	-	-	-	-	A	-	-	-	T	-	-	-	-	-
	BS_2*		-	-	-	-	-	-	-	-	-	-	-	-	-	-	-	-	-	-	-	-	-	-	-	-	-	-	T	-	-	-	-	-	-	-	-	-	-	x	-	-	-	-	-	-	-	A	-	-	T	-	G	-	-	-	-	-	-	-	-	-	-	-	T	-	-	-	-	-
Borotal 2	Bt_1		-	-	-	-	-	-	-	-	C	-	-	-	-	-	-	G	-	-	-	-	-	-	-	-	-	-	T	-	-	-	-	-	-	-	-	-	-	x	-	-	-	-	-	-	-	A	-	-	-	-	-	-	-	-	-	-	-	-	-	-	-	-	T	-	-	-	-	-
	Bt_2*		-	-	-	-	-	-	-	-	-	-	C	-	-	-	-	-	-	-	-	-	-	-	-	-	-	-	T	-	-	-	-	-	-	-	-	-	-	x	-	-	-	-	-	-	G	A	-	-	-	-	G	-	-	-	-	-	-	-	-	-	-	C	T	-	-	-	-	-
Dcholin 2	D_1*		-	-	-	-	-	-	-	G	-	-	-	-	-	-	-	G	-	-	-	-	-	-	-	-	-	-	T	-	-	-	-	-	-	-	-	-	-	x	-	-	-	-	-	-	-	A	-	-	-	-	-	-	-	T	-	-	-	-	-	-	-	-	T	-	-	-	-	-
Ismailovo	Is_1		-	-	-	-	-	A	-	-	-	-		-	-	-	T	-	-	-	A	-	-	-	-	-	-	-	T	-	-	-	-	-	T	-	-	-	-	-	-	-	-	G	T	C	-	A	-	-	-	-	-	-	-	T	-	-	-	-	-	-	-	-	T	C	-	-	-	-
	Is_2		-	-	-	-	-	-	-	-	-	-	-	-	-	-	-	-	-	-	-	-	-	-	-	-	-	-	T	-	-	-	-	-	-	-	-	-	-	x	-	-	-	-	-	-	-	-	-	-	-	-	-	-	-	-	-	-	-	-	-	-	-	-	-	-	-	-	-	-
	Is_4		-	-	-	-	-	-	-	-	-	-	-	-	-	-	-	-	-	-	-	-	-	-	-	-	-	-	-	-	-	-	-	-	-	-	-	-	-	-	-	-	-	-	-	-	-	-	-	-	-	-	-	-	-	-	-	-	-	-	-	-	-	-	-	-	-	-	-	-
Justyd 12	J12_1		-	-	-	-	-	-	-	-	C	-	-	-	-	-	-	G	-	-	-	-	-	-	-	-	-	-	T	-	-	-	-	-	-	-	-	-	-	x	-	-	-	-	-	-	-	A	-	-	-	-	-	-	-	-	-	-	-	-	-	-	-	-	T	-	-	-	-	-
	J12_3		-	-	-	-	-	-	-	-	-	-	-	-	-	-	-	-	-	-	-	-	-	-	-	-	-	-	T	-	-	-	-	-	-	-	-	-	-	x	-	T	-	-	-	-	-	A	-	-	-	-	G	-	-	-	-	-	-	-	-	-	-	-	T	-	-	-	-	-
	J12_6*		-	-	-	-	-	-	-	-	-	-	-	-	-	-	-	-	-	-	-	-	-	-	-	-	-	-	T	-	-	-	-	-	-	-	-	-	C	x	-	-	-	G	T	C	-	A	-	-	-	-	-	-	-	T	-	-	-	-	-	-	-	-	T	C	-	-	-	-
	J12_7*		-	-	-	-	-	-	-	-	C	-	-	-	-	-	-	G	-	-	-	-	-	-	-	-	-	-	T	-	-	-	-	-	-	-	-	-	-	x	-	-	-	-	-	-	-	A	-	-	-	-	-	-	-	-	-	-	-	-	-	-	-	-	T	-	-	-	-	-
	J12_8*		-	-	-	G	-	-	-	-	-	-	-	-	G	-	-	-	-	-	-	-	-	-	-	-	-	-	T	-	-	-	-	-	-	-	-	-	-	x	-	-	-	-	-	-	-	A	-	-	-	-	-	-	-	T	-	-	-	-	-	-	-	-	T	-	-	-	-	-
	J12_9		-	-	-	-	-	-	-	-	-	-	-	x	-	G	-	-	-	-	-	-	-	-	-	-	-	-	T	-	-	-	-	-	-	-	-	-	x	x	-	-	-	G	T	C	-	A	-	-	-	-	-	-	-	T	-	-	-	-	-	-	-	-	T	C	-	-	-	-
Justyd 22	J22_1*		-	-	-	-	-	-	-	-	C	-	-	-	-	-	-	G	-	-	-	-	-	-	-	-	-	-	T	-	-	-	-	-	-	-	-	-	-	x	-	-	-	-	-	-	-	A	-	-	-	-	-	-	-	-	-	-	-	-	-	-	-	-	T	-	-	-	-	-
Kuturguntas 1	K_1*		-	-	-	-	-	-	-	-	-	-	-	-	-	-	-	-	-	-	-	-	-	-	-	-	-	-	-	-	-	-	-	-	-	-	-	-	-	x	-	-	-	-	-	-	-	-	-	-	-	-	-	-	-	-	-	-	-	-	-	-	-	-	-	-	-	-	-	-
Kolbino 1	KOL_1		-	-	-	-	-	-	-	-	-	-	-	-	-	-	-	-	-	-	-	-	x	T	-	-	-	-	T	-	-	-	-	-	-	-	-	-	-	x	-	-	-	-	-	-	-	A	-	-	-	-	-	-	-	T	-	-	-	-	-	-	-	-	T	-	-	-	-	-
	KOL_2		-	-	-	-	-	-	-	-	-	-	-	x	-	-	-	-	-	-	-	-	-	-	-	-	-	-	-	-	-	-	-	-	-	-	-	r	-	x	-	-	-	-	-	-	-	-	-	-	-	-	-	-	-	-	-	-	-	-	-	A	-	-	-	-	-	-	-	-
	KOL_3		x	-	-	-	-	-	-	-	-	-	-	r	-	-	y	-	-	-	-	-	-	-	-	-	-	-	T	x	x	x	-	-	-	-	-	-	-	x	-	-	r	-	-	-	-	A	-	-	-	-	G	-	-	-	-	-	-	x	-	-	-	-	T	-	-	-	-	-
	KOL_5		x	x	-	-	-	x	x	x	x	-	-	-	-	-	x	x	x	-	-	-	-	-	x	x	x	-	T	-	-	-	x	x	x	x	x	x	-	x	x	x	x	G	-	-	-	A	-	-	-	-	-	-	-	-	-	-	-	-	A	-	-	-	T	-	-	-	-	-

			M01		M02			M03		M04		M05		M06	M07		M08			M09		M10	M11			M12		M13		M14			M15			M16			M17	M18	M19			M20		M21		M22		M23			M24			M25			M26	M27	M28				M29		M30			
	Lab	SNP	456	477	654	663	689	3010	3027	4173	4216	4529	4553	4580	4824	4833	4883	4917	4924	5171	5178	5843	6340	6371	6392	6776	6779	6975	7028	8251	8273	9bp del	8392	8393	8414	8962	8989	8994	9090	10034	10118	10142	10143	10398	10400	10873	10876	11719	11732	11947	11965	11969	12308	12338	12346	12705	12732	12738	13263	13626	13708	13711	13720	13734	14766	14783	14793	14798	14815	14839
Site	code	ref	C	T	T	A	T	G	T	A	T	A	T	G	A	A	C	A	G	A	C	A	C	C	T	T	A	T	C	G	C	-	G	C	C	A	G	G	T	T	T	C	G	A	C	T	A	G	T	A	C	G	A	T	C	C	T	T	A	C	G	G	C	T	C	T	A	T	C	A
Moinak 2	Mo_1*		-	-	-	-	-	A	-	-	-	-	-	-	-	-	T	-	-	-	A	-	-	-	-	-	-	-	T	-	-	-	-	-	T	-	-	-	-	x	-	-	-	G	T	C	-	A	-	-	-	-	-	-	-	T	-	-	-	-	-	-	T	-	T	C	-	-	T	-
	Mo_2*		-	-	-	-	-	A	-	-	-	-	-	-	-	-	T	-	-	-	A	-	-	-	-	-	-	-	T	-	-	-	-	-	T	-	-	-	-	x	-	-	-	G	T	C	-	A	-	-	-	-	-	-	-	T	-	-	-	-	-	-	-	-	T	C	-	-	-	-
Novozave-	NOV_5		-	-	-	-	-	-	-	-	-	-	-	-	-	-	-	-	-	-	-	-	-	-	-	-	-	-	-	-	-	-	-	-	-	-	-	-	-	x	-	-	-	-	-	-	-	-	-	-	-	-	-	-	-	-	-	-	-	-	-	-	-	-	-	-	-	-	-	-
dennoe 2	NOV_7		-	C	-	-	-	A	-	-	-	-	-	-	-	-	-	-	-	-	-	-	-	-	-	-	-	-	-	-	-	-	-	-	-	-	-	-	-	x	-	-	-	-	-	-	-	-	-	-	-	-	-	-	-	-	-	-	-	-	-	-	-	-	-	-	-	-	-	-
	NOV_9		-	-	-	-	-	-	-	-	C	-	-	-	-	-	-	G	-	-	-	-	-	-	-	-	-	-	T	-	-	-	-	-	-	-	-	-	-	x	-	-	-	-	-	-	-	A	-	-	-	-	-	-	-	-	-	-	-	-	-	-	-	-	T	-	-	C	-	G
	NOV_10		-	-	-	-	-	-	-	-	-	-	-	-	-	-	-	-	-	-	-	-	-	T	-	-	-	-	T	-	-	-	-	-	-	-	-	-	-	x	-	-	-	-	-	-	-	A	-	-	-	-	-	-	-	T	-	-	-	-	-	-	-	-	T	-	-	-	-	-
Pokrovka	Pr1		x	x	x	x	x	x	x	x	x	x	x	x	x	x	x	x	x	x	x	x	x	x	x	x	x	-	T	x	x	x	x	x	x	x	x	x	x	x	x	x	x	x	x	x	x	x	x	x	x	x	G	-	-	x	x	x	x	x	x	x	x	x	x	-	-	-	-	-
	Pr3		x	x	x	x	x	x	x	x	x	x	x	x	-	-	x	x	x	-	-	x	x	x	x	x	x	x	x	x	x	x	x	x	x	x	x	x	-	x	x	x	x	G	T	x	x	x	x	x	x	x	x	x	x	x	x	x	x	-	x	x	x	x	x	x	x	x	x	x
	Pr4		x	x	x	x	x	x	x	x	x	x	x	x	x	x	x	x	x	x	x	x	x	x	x	x	x	x	x	x	x	x	x	x	x	x	x	x	x	x	x	x	x	x	x	x	x	x	x	x	x	x	x	x	x	x	x	x	x	x	x	x	x	x	x	x	x	x	x	x
	Pr5		x	x	x	x	x	x	x	x	x	x	x	x	x	x	x	x	x	x	x	x	x	x	x	x	x	x	x	x	x	x	x	x	x	x	x	x	x	x	x	x	x	x	x	x	x	x	x	x	x	x	x	x	x	x	x	x	x	x	x	x	x	x	x	x	x	x	x	x
	Pr6		x	x	x	x	x	x	x	x	x	x	x	x	x	x	x	x	x	x	x	x	x	x	x	x	x	x	x	x	x	x	x	x	x	x	x	x	x	x	x	x	x	x	x	x	x	x	x	x	x	x	x	x	x	x	x	x	x	x	x	x	x	x	x	x	x	x	x	x
	Pr7		x	x	x	x	x	x	x	x	x	x	x	x	x	x	x	x	x	x	x	x	x	x	x	x	x	x	x	x	x	x	x	x	x	x	x	x	x	x	x	x	x	G	-	-	-	x	x	x	x	x	x	x	x	x	x	x	x	x	x	x	x	x	x	x	x	x	x	x
	Pr8		x	x	x	x	x	x	x	x	x	x	x	x	x	x	x	x	x	x	x	x	x	x	x	x	x	x	x	x	x	x	x	x	x	x	x	x	x	x	x	x	x	x	x	x	x	x	x	x	x	x	x	x	x	x	x	x	x	x	x	x	x	x	x	x	x	x	x	x
	Pr9		x	x	x	x	x	x	x	x	x	x	x	x	x	x	x	x	x	x	x	x	x	x	x	x	x	x	x	x	x	x	x	x	x	x	x	x	x	x	x	x	x	x	x	x	x	x	x	x	x	x	x	x	x	x	x	x	x	x	x	x	x	x	x	x	x	x	x	x
	Pr10		x	x	x	x	x	x	x	x	x	x	x	x	x	x	x	x	x	x	x	x	x	x	x	x	x	-	-	x	x	x	x	x	x	x	x	x	x	x	x	x	x	x	x	x	x	x	x	x	x	x	x	x	x	x	x	x	x	x	x	x	x	x	x	x	x	x	x	x
	Pr11		x	x	x	x	x	x	x	x	x	x	x	x	x	x	x	x	x	x	x	x	x	x	x	x	x	x	x	x	x	x	x	x	x	x	x	x	x	x	x	x	x	x	x	x	x	x	x	x	x	x	x	x	x	x	x	x	x	x	x	x	x	x	x	x	x	x	x	x
	Pr13		x	x	x	x	x	x	x	x	x	x	x	x	x	x	x	x	x	x	x	x	x	x	x	x	x	x	x	x	x	x	x	x	x	x	x	x	x	x	x	x	x	x	x	x	x	x	x	x	x	x	x	x	x	x	x	x	x	x	x	x	x	x	x	x	x	x	x	x
Tar Asu	Ta_1		-	-	-	-	-	-	-	-	-	-	-	-	-	-	-	-	-	-	-	-	-	-	-	-	-	-	T	-	-	-	-	-	-	-	-	-	-	-	-	-	-	-	-	-	-	A	-	-	-	-	G	-	-	-	-	-	-	-	-	-	-	-	T	-	-	C	-	-
Ulandryk 1	U1_1		-	-	-	-	-	-	-	-	-	-	-	-	-	-	-	-	-	-	-	-	-	-	-	-	-	-	T	A	-	-	-	-	-	-	-	A	-	x	-	-	-	-	-	-	-	A	-	G	-	-	-	-	-	T	-	-	-	-	-	-	-	-	T	-	-	-	-	-
	U1_2		-	-	-	-	-	-	-	-	-	-	-	-	-	-	-	-	-	-	-	-	-	-	-	-	-	-	T	-	-	-	-	-	-	-	-	-	C	x	-	-	-	G	T	C	-	A	-	-	-	-	-	-	-	T	-	-	-	-	-	-	-	-	T	C	-	-	-	-
Ulandryk 2	U2_1*		-	-	-	-	-	-	-	-	-	-	-	-	-	-	-	-	-	-	-	-	-	-	C	-	-	-	T	-	-	-	x	x	x	-	-	-	-	x	-	-	-	-	-	-	-	A	-	-	-	-	-	C	-	-	-	-	-	-	A	-	-	-	T	-	-	-	-	-
	U2_2		-	-	-	-	-	-	-	-	-	-	-	-	-	-	-	-	-	-	-	-	-	-	C	-	-	-	T	-	-	-	-	-	-	-	-	-	-	x	-	-	-	-	-	-	-	A		-		-	-	C	-	-	-	-	-	-	A	-	-	-	T	-	-	-	-	-
Ulandryk 4	U4_1		-	-	-	-	-	-	-	-	-	-	-	-	-	-	-	-	-	-	-	-	-	-	-	-	-	-	T	-	-	-	-	-	-	-	-	-	-	x	-	-	-	-	-	-	G	A	-	-	-	-	G	-	-	-	-	-	-	-	-	-	-	C	T	-	-	-	-	-
	U4_4		-	-	-	-	-	-	-	-	-	-	-	-	-	-	-	-	-	-	-	-	-	-	-	-	-	x	x	-	-	-	x	x	x	-	-	-	-	x	-	-	-	-	-	-	-	A	-	-	-	-	G	-	-	-	-	-	-	-	-	-	-	-	x	-	-	C	-	-
Verch Kal'dzin 1	VK1_1		-	-	-	-	-	-	-	-	-	-	-	-	-	-	-	-	-	-	-	-	-	-	-	-	-	-	T	-	-	-	-	-	-	-	-	-	-	x	-	-	-	G	-	-	-	A	-	-	-	-	G	-	-	-	-	G	-	-	-	-	-	-	T	-	-	C	-	-
Zevakino	Ze_2		-	-	-	-	-	-	-	-	C	-	-	-	-	-	-	G	-	-	-	-	-	-	-	-	-	-	T	-	-	-	-	-	-	-	-	-	-	-	-	-	-	-	-	-	-	A	-	-	-	-	-	-	-	-	-	-	-	-	-	-	-	-	T	-	-	-	-	-
	Ze_3		-	-	-	-	-	-	-	-	-	-	-	-	-	-	-	-	-	-	-	-	-	-	-	-	-	-	T	-	-	-	-	-	-	-	-	-	-	-	-	-	-	G	-	-	-	A	-	-	-	-	G	-	-	-	-	-	-	-	-	-	-	-	T	-	-	C	-	-
	Ze_4		-	-	-	-	-	A	-	-	-	-	-	-	-	-	T	-	-	-	A	-	-	-	-	-	-	-	T	-	-	-	-	-	T	-	-	-	-	-	-	-	-	G	T	C	-	A	-	-	-	-	-	-	-	T	-	-	-	-	-	-	-	-	T	C	-	-	-	-
	Ze_5		-	-	-	-	-	-	-	-	-	T	-	-	-	-	-	-	-	-	-	-	-	-	-	-	-	-	T	A	-	-	-	-	-	-	-	-	-	C	-	-	-	G	-	-	-	A	-	-	-	-	-	-	-	T	-	-	-	-	-	-	-	-	T	-	-	-	-	-
	Ze_6		-	-	-	-	-	-	-	-	-	-	-	-	-	-	-	-	-	-	-	-	-	-	-	-	-	-	T	-	-	-	-	-	-	-	-	-	-	x	-	-	-	G	T	C	-	A	-	-	-	A	-	-	-	T	-	-	G	-	-	-	-	-	T	C	-	-	-	-
	Ze_7		-	-	-	-	-	-	-	-	-	-	-	-	-	-	-	-	-	-	-	-	-	-	-	-	-	-	T	-	-	-	-	-	-	-	-	-	-	x	-	-	-	-	-	-	-	A	-	-	-	-	G	-	-	-	-	-	-	-	-	-	-	-	T	-	-	-	-	-
	Ze_8		-	-	-	-	-	A	-	-	-	-	-	-	-	-	T	-	-	-	A	-	-	-	-	-	-	-	T	-	-	-	-	-	T	-	-	-	-	x	-	-	-	G	T	C	-	A	-	-	-	-	-	-	-	T	-	-	-	-	-	-	-	-	T	C	-	-	-	-
	Ze_9		-	-	-	-	C	A	-	-	-	-	-	-	-	-	T	-	-	-	A	-	-	-	-	-	-	-	T	-	-	-	-	-	T	-	-	-	-	-	-	-	-	G	T	C	-	A	-	-	-	-	-	-	-	T	-	-	-	-	-	-	-	-	T	C	-	-	-	-

Supplementary Table 7. Summary statistics of defined sample groups.

Number of individuals (n); number of different Haplotypes (k); frequency of mitochondrial lineages common in modern populations of West Eurasia (WEA) and East Eurasia (EEA) with 95% confidence interval (CI); Haplotype diversity (H); nucleotide diversity (π); Fu's F_S values (F_S) with according p-values (F_S p-value); significant F_S values are given in bold (significance level: p-value ≤ 0.05).

	cultural groups	date [cent. BC]	n	k	WEA	EEA	95% CI	$\hat{H} \pm sd$	$\pi \pm sd$	F_S	F_S p-value
West	initial Scythians	8th – 7th	3	3	1	0	±0.35	1.000±0.272	0.009±0.008	-0.077	0.246
	Scythians	6th – 2nd	19	17	0.74	0.26	±0.21	0.988±0.021	0.018±0.010	**-9,140**	0.000
	Sarmatians	5th – 2nd	11	11	0.82	0.18	±0.25	1.000±0.039	0.022±0.013	**-4,998**	0.002
East	Zevakino-Chilikta	9th – 7th	11	11	0.54	0.46	±0.30	1.000±0.039	0.013±0.008	**-7,291**	0.000
	Aldy Bel	7th – 6th	15	14	0.33	0.67	±0.25	0.991±0.028	0.018±0.010	**-7,344**	0.000
	Tagar/Tes	8th – 1st cent. AD	16	12	0.50	0.50	±0.25	0.958±0.036	0.020±0.011	-2,701	0.081
	Pazyryk	4th – 3rd	71	47	0.48	0.52	±0.12	0.986±0.005	0.019±0.010	**-25,095**	0.000

Supplementary Table 8. Genetic distances between sample groups associated with the Scythian culture.

F_{ST}-values are shown in the lower diagonal and according p-values are shown in the upper diagonal. Significant F_{ST}-values in bold (significance level: p-value ≤ 0.05)

	Tagar/Tes	Aldy Bel	Pazyryk	Zevakino-Chilikta	initial-Scythians	Scythians	Early Sarmatians
Tagar/Tes		0.249±0.012	0.297±0.014	0.228±0.012	0.836±0.009	0.439±0.016	0.357±0.015
Aldy Bel	0.011		0.721±0.017	0.468±0.015	0.802±0.010	0.729±0.016	0.047±0.006
Pazyryk	0.005	-0.010		0.679±0.016	0.875±0.010	0.464±0.015	0.022±0.005
Zevakino-Chilikta	0.015	-0.002	-0.011		0.595±0.014	0.554±0.013	0.041±0.006
initial-Scythians	-0.088	-0.059	-0.076	-0.028		0.989±0.003	0.950±0.006
Scythians	-0.002	-0.014	-0.001	-0.007	-0.122		0.670±0.013
Sarmatians	0.006	**0.048**	**0.042**	**0.052**	-0.101	-0.013	

Supplementary Table 9. Model selection results for genetic continuity between the two eastern Scythian sample groups. Given for the 11 candidate scenarios considered (Supplementary Fig. 2) are the posterior probabilities using a simple rejection method, a logistic regression method and a non-linear regression method based on neural networks[26]. Model posteriors are given for two different thresholds of tolerance (1% and 0.5% of simulations with summary statistics closest to observed values), and are only displayed when enough simulations were retained within the tolerance levels. All analyses were performed using the package *abc*[28] in R v.2.15.1[29].

	1% closest			*0.5% closest*		
	rejection	*logistic*	*neural*	*rejection*	*logistic*	*neural*
1	0.000	-	-	0.000	-	-
2	0.000	0.000	0.003	0.000	0.000	0.001
3	0.003	0.003	0.063	0.004	0.001	0.044
4	0.004	0.003	0.070	0.004	0.002	0.063
5	0.002	0.001	0.042	0.002	0.001	0.025
**6**	**0.234**	**0.993**	**0.482**	**0.252**	**0.995**	**0.568**
7	0.000	-	-	0.000	-	-
8	0.000	-	-	0.000	-	-
9	**0.441**	0.000	0.168	**0.457**	0.000	0.175
10	0.300	0.000	0.157	0.273	0.000	0.112
11	0.017	0.000	0.016	0.009	0.000	0.012

Supplementary Table 10. Evaluation of confidence in the model choice procedure for the demographic history of the two eastern Scythian sample groups. For each scenario, we simulated 100 sets of summary statistics, and used these as the observed values in the model selection (using a logistic regression procedure based on the 1% closest simulations). Given for each set of simulated scenarios are the proportions of times that the model was correctly identified as the preferred one, as well as the proportions of times it was misidentified as another scenario. All analyses were performed using the package *abc*[28] in R v.2.15.1[29].

	Identified as							
Simulated as	*2*	*3*	*4*	*5*	*6*	*9*	*10*	*11*
2	**0.87**	0.00	0.00	0.00	0.00	0.00	0.00	0.06
3	0.01	**0.66**	0.46	0.00	0.02	0.04	0.00	0.00
4	0.01	0.31	**0.47**	0.08	0.00	0.01	0.03	0.00
5	0.01	0.00	0.05	**0.86**	0.00	0.00	0.00	0.05
**6**	0.04	0.00	0.00	0.00	**0.85**	0.13	0.03	0.03
9	0.01	0.03	0.02	0.01	0.09	**0.70**	0.26	0.00
10	0.00	0.00	0.00	0.03	0.04	0.12	**0.65**	0.02
11	0.05	0.00	0.00	0.02	0.00	0.00	0.03	**0.84**

Supplementary Table 11. Posteriors of demographic and mutational model parameters for the history of the two eastern Scythian sample groups. For each model parameter the median, mean and 95% confidence interval are given of the posterior distributions. Values obtained by performing ABC analyses based on a non-linear regression using neural networks[26] on the 0.5% closest simulations, with the package *abc*[28] in R v.2.15.1[29].

Model parameter	*Median*	*Mean*	*2.5%*	*97.5%*
Population Size (*N*)	$5.1.10^{5}$	$5.3.10^{5}$	$1.3.10^{5}$	$9.6.10^{5}$
Growth rate *r* (/generation)	$8.0.10^{-3}$	$8.1.10^{-3}$	$2.5.10^{-3}$	$1.4.10^{-2}$
Mutation rate (/locus/generation)	$2.2.10^{-3}$	$2.1.10^{-3}$	$8.0.10^{-4}$	$3.0.10^{-3}$

Supplementary Table 12. Model selection results for the origins and relations between Scythian populations from the Iron Age. Given for the four candidate scenarios considered (Figure S4a) are the posterior probabilities using a simple rejection method, a logistic regression method and a non-linear regression method based on neural networks[26]. Model posteriors are given for two different thresholds of tolerance (1% and 0.5% of simulations with summary statistics closest to observed values), and are only displayed when enough simulations were retained the tolerance levels. All analyses performed using the package *abc*[28] in R v.2.15.1[29].

	1% closest			0.5 % closest		
Scenario	*Rejection*	*Logistic*	*Neural*	*Rejection*	*Logistic*	*Neural*
Eastern 1	0.278	0.003	0.055	0.195	0.006	0.017
Eastern 2	0.100	0.000	0.000	0.051	0.000	0.000
Western	**0.472**	0.237	0.250	**0.566**	0.286	0.267
**Multiregion**	0.150	**0.760**	**0.695**	0.189	**0.708**	**0.715**

Supplementary Table 13. Model selection for continuity of gene flow under the Multi-region model of origins and relations between Scythian populations from the Iron Age. Given are the posterior probabilities for the continuous gene flow model in pair wise comparisons to alternative scenarios (see Figure S4b for details). Model selection was performed using a simple rejection method, a logistic regression method and a non-linear regression method based on neural networks[26]. Model posteriors are given for two different thresholds of tolerance (1% and 0.5% of simulations with summary statistics closest to observed values). Values larger than 0.50 imply the continuous gene flow model is preferred over the alternative model. All analyses performed using the package *abc*[28] in R v.2.15.1[29].

	1% closest			0.5 % closest		
Alternative model	*Rejection*	*Logistic*	*Neural*	*Rejection*	*Logistic*	*Neural*
First gene flow from West-Europeans into east Scythians, then gene flow from east into west Scythians	0.525	0.888	0.963	0.542	0.902	0.926
Two separate periods of gene flow between east and west Scythians	0.498	0.704	0.746	0.508	0.704	0.767
First gene flow from west into east Scythians, then gene flow from east into west Scythians	0.550	0.802	0.683	0.565	0.785	0.931

Supplementary Table 14. Evaluation of confidence in the model choice procedure for the origins and relations between Scythian populations from the Iron Age. For each scenario, we simulated 100 sets of summary statistics, and used these as the observed values in the model selection (using a neural networks procedure based on the 1% closest simulations). Given for each set of simulated scenarios are the proportions of times that the model was correctly identified as the preferred one, as well as the proportions of times it was misidentified as another scenario. Analyses performed using the package *abc*[28] in R v.2.15.1[29].

	Identified as			
Simulated as	*Eastern 1*	*Eastern 2*	*Western*	*Multiregion*
Eastern 1	**0.70**	0.28	0.02	0.00
Eastern 2	0.22	**0.78**	0.00	0.00
Western	0.06	0.02	**0.89**	0.03
**Multiregion**	0.01	0.00	0.09	**0.90**

Supplementary Table 15. Posterior distribution of model parameters of the preferred scenario (Multi-region) for the origins and relations between Scythian populations from the Iron Age. Given are the median, mean and 95% percentage confidence intervals for population size (N), population growth rate (r) and migration rate (m) parameters, obtained by performing an non-linear regression (using the neural networks method using the package *abc*[28] in R v.2.15.1[29]. See Figure S4a for details on the preferred scenario (Multi-region hypothesis).

Parameter	*Median*	*Mean*	*2.5% percentile*	*97.5% percentile*
N_{WEU}	$5.6.10^{5}$	$5.5.10^{5}$	$8.3.10^{4}$	$9.8.10^{5}$
N_{WScyth}	$5.0.10^{5}$	$5.0.10^{5}$	$5.2.10^{4}$	$9.7.10^{5}$
N_{EScyth}	$2.8.10^{5}$	$3.5.10^{5}$	$2.9.10^{4}$	$9.2.10^{5}$
N_{Han}	$4.7.10^{5}$	$4.9.10^{5}$	$8.9.10^{4}$	$9.7.10^{5}$
r_{WEU}	$9.0.10^{-3}$	$9.4.10^{-3}$	$4.5.10^{-3}$	$1.7.10^{-2}$
r_{WScyth}	$1.0.10^{-2}$	$1.0.10^{-2}$	$6.0.10^{-4}$	$1.9.10^{-2}$
r_{EScyth}	$1.4.10^{-2}$	$1.3.10^{-2}$	$1.1.10^{-3}$	$2.0.10^{-2}$
r_{Han}	$6.9.10^{-3}$	$7.1.10^{-3}$	$4.0.10^{-4}$	$1.1.10^{-2}$
$m_{WS->ES}$	$8.2.10^{-3}$	$8.0.10^{-3}$	$5.3.10^{-3}$	$9.8.10^{-3}$
$m_{ES->WS}$	$5.2.10^{-3}$	$5.3.10^{-3}$	$1.7.10^{-3}$	$9.4.10^{-3}$

Supplementary Table 16. Model selection results for placing the ancestry of Andronovo/Fedorovo on the scenario of multiregional origin of Scythian populations. Given for the four candidate scenarios considered (Supplementary Fig. 4c) are the posterior probabilities using a simple rejection method, a logistic regression method and a non-linear regression method based on neural networks[26]. Model posteriors are given for two different thresholds of tolerance (1% and 0.5% of simulations with summary statistics closest to observed values). All analyses performed using the package *abc*[28] in R v.2.15.1[29].

Scenario (ancestral to:)	1% closest			0.5 % closest		
	Rejection	*Logistic*	*Neural*	*Rejection*	*Logistic*	*Neural*
western Europeans	0.005	0.000	0.000	0.004	0.000	0.000
western Scythians	0.124	0.010	0.011	0.098	0.011	0.004
**eastern Scythians**	**0.648**	**0.988**	**0.978**	**0.651**	**0.986**	**0.991**
Han Chinese	0.223	0.002	0.012	0.248	0.002	0.005

Supplementary Table 17. Evaluation of confidence in the model choice procedure for the ancestry of Andronovo/Fedorovo in relation to Scythian populations. For each scenario, we simulated 100 sets of summary statistics, and used these as the observed values in the model selection (using a neural networks procedure based on the 1% closest simulations). Given for each set of simulated scenarios are the proportions of times that the model was correctly identified as the preferred one, as well as the proportions of times it was misidentified as another scenario. Analyses performed using the package *abc*[28] in R v.2.15.1[29].

Simulated as ancestral to	*Identified as ancestral to*			
	W-Europeans	W-Scythians	E-Scythians	Han Chinese
W-Europeans	**0.87**	0.11	0.00	0.02
W-Scythians	0.27	**0.72**	0.01	0.00
E-Scythians	0.00	0.01	**0.88**	0.11
Han Chinese	0.00	0.00	0.06	**0.94**

Supplementary Table 18. Evaluation of confidence in the model choice procedure for the contemporary descent from Iron Age Scythian populations. For each scenario, we simulated 250 sets of summary statistics, and used these as the observed values in the model selection (using a logistic regression based on the 1% closest simulations). Given for each set of simulated scenarios are the proportions of times that the model was correctly identified as the preferred one, as well as the proportions of times it was misidentified as another scenario. Analyses performed using the package *abc*[28] in R v.2.15.1[29].

Simulated as	*Identified as*			
	Ancestral to W-Scythians	Descent from W-Scythians	Ancestral to E-Scythians	Descent from E-Scythians
Ancestral to W-Scythians	**0.93**	0.06	0.01	0.00
Descent from W-Scythians	0.10	**0.90**	0.00	0.00
Ancestral to E-Scythians	0.01	0.00	**0.96**	0.03
Descent from E-Scythians	0.00	0.00	0.03	**0.97**

Supplementary Table 19. Sample characteristics for 86 contemporary Eurasian populations.

Given for each population, are its sample site, code, sample size (*S*) and original publication reference. Also given are four within-population summary statistics for each contemporary sample: the number of haplotypes (*Nh*), the average number of pairwise differences (*k*), nucleotide diversity (π) and Tajima's *D*.

Ethnic/language group	*Sample*	*Site Name*	*Code*	*S*	*Nh*	*k*	π	*D*	*Reference*
Caucasus	Abazinians	Caucasus region	ABA	23	19	4.530	0.015	-2.018	76
	Chechens	Caucasus region	CHE	23	18	4.229	0.014	-1.668	76
	Cherkessians	Caucasus region	CRK	44	35	4.706	0.016	-2.046	76
	Darginians	Caucasus region	DAR	37	25	4.478	0.015	-2.059	76
	Georgians	Batumi, Georgia	GES	28	26	4.685	0.016	-2.047	77
	Ingushans	Caucasus region	ING	35	25	4.316	0.014	-1.483	76
	Kabardinian	Caucasus region	KAB	50	35	4.589	0.015	-2.248	76
Chinese	Northern Han	Northern China	HAN	50	38	5.777	0.019	-1.738	14
Dravidian	Brahui	Southwestern Pakistan, Baluchistan	BRQ	30	19	4.570	0.015	-1.704	78
East-Slavic	Russians	Russia	RUS	50	42	4.272	0.014	-1.775	13
Indo-European	Armenians	Yerevan, Armenia	AMS	30	27	5.444	0.018	-2.006	77
	Baluch	Southwestern Pakistan, Baluchistan	BAQ	30	22	4.460	0.015	-1.781	78
	Gilaki	Northern Iran	GIQ	30	26	5.200	0.017	-1.835	78
	Hazara	NW Frontier Province / Balochistan	HZQ	23	21	5.953	0.020	-1.595	78
	Iranians	Tehran, Iran	IRA	50	45	5.416	0.017	-1.939	79
		Tehran, Iran	IRS	30	25	4.954	0.017	-1.852	77
		Northern Iran	IRTN	22	22	5.836	0.020	-1.685	79
		Southern Iran	IRTS	50	42	6.128	0.022	-2.160	79
	Kalash	Northwestern Frontier Province	KLQ	40	10	3.505	0.012	0.148	78
	Kurdish	Turkmenistan	KTQ	30	21	6.021	0.020	-1.401	78
	Pathan	NW Frontier Province / Balochistan	PTQ	40	36	5.069	0.017	-2.091	78
	Persians	Central / southern Central Iran	PEQ	40	36	5.376	0.018	-1.944	78
		Eastern Iran	PER	50	44	5.375	0.018	-2.066	80
	Shugnan	High Pamirs, Pakistan	SHQ	40	30	5.390	0.018	-1.746	78
	Tajiks	Boukhara	TAB	24	23	4.239	0.014	-1.781	81
		Ching (Penjinkent)	TDS	48	14	5.832	0.020	-1.063	82
		Urmetan (Aïni)	TDU	31	22	4.699	0.016	-1.368	82

Ethnic/language group	*Sample*	*Site Name*	*Code*	*S*	*Nh*	*k*	*π*	*D*	*Reference*
		Agalic (Samarkand)	TJA	40	22	4.228	0.014	-1.545	83
		Nimich (Gharm)	TJE	32	21	5.105	0.017	-1.382	82
		Ferghana (Kaptarana)	TJK	31	29	4.691	0.016	-1.779	83
		Navdi (Gharm)	TJN	39	28	4.210	0.014	-2.096	82
		Richtan (Kokand)	TJR	34	24	6.057	0.020	-1.888	83
		Nouchor (Tadjikabad)	TJT	29	26	4.855	0.016	-1.909	82
	Yagnobs	Saferodak (Douchambe)	TJY	40	17	4.210	0.014	-0.622	82
Mongolian	Buryats	Irkoutsk, lake Baïkal	BUR	50	43	5.703	0.019	-1.889	84
	Daur	Ewenkizu Zizhiqi, China	DAU	43	29	6.195	0.021	-1.734	85
	Kalmyks	Kalmyk Republic	KAL	50	42	5.933	0.020	-1.945	80
	Mongolians	Mongolia	MOC	48	41	6.527	0.021	-1.924	86
		Uiaan-Bataar, Mongolia	MOD	40	32	7.196	0.024	-1.719	80
		Hohhot, China	MOH	50	42	5.660	0.019	-1.868	86
Tungusic	Evenks	Buryat Republic	EEV	40	12	5.199	0.017	0.047	80
	Khamnigans	Buryat Republic	KHM	50	38	6.204	0.021	-1.868	80
	Evenks	Krasnoyarsk region	WEV	50	25	5.682	0.019	-1.344	80
Turkic	Altai-Kazakh	Tobeler	AKZ	41	25	4.907	0.017	-1.616	CNRS database
	Altai-Kishi	Altai Republic	AKD	50	38	5.963	0.020	-1.524	80
		Kulada	AKI	44	34	5.873	0.020	-1.687	CNRS database
	Azeri	Baku, Azerbadjan	AZS	30	28	6.336	0.021	-1.801	77
	Karakalpaks	Mouinak (Noukous)	KKK	50	44	5.294	0.018	-1.944	87
		Chimbaï (Noukous)	OTU	50	41	5.856	0.020	-1.906	87
	Kazakhs	Kegen valley, Almaty, Kazakhstan	KAC	50	41	6.305	0.021	-1.685	88
		Kashen, Xinjang, China	KAY	30	27	6.315	0.021	-1.553	89
		Kungrat (Noukous)	KAZ	50	44	5.113	0.017	-1.936	87
		Gasli (Bukara)	LKZ	30	24	5.075	0.017	-1.589	82
	Khakassians	Kazanovka	HKS	39	24	5.155	0.017	-1.319	CNRS database
		Khakassians	KH	50	34	6.594	0.022	-1.275	84
	Kyrgyz	Bichkek	KIB	29	26	6.833	0.023	-1.556	81
		Ordaj (Andijan)	KRA	48	32	5.159	0.017	-1.947	82
		Ak-Mouz	KRB	30	24	5.101	0.017	-1.823	81
		Talas, Kyrgyzie	KRC	48	39	6.170	0.021	-1.773	88

Ethnic/language group	*Sample*	*Site Name*	*Code*	*S*	*Nh*	*k*	*π*	*D*	*Reference*
		Djerghetal (Naryn)	KRG	20	19	5.716	0.019	-1.270	82
		Kulanak	KRL	24	20	5.645	0.019	-1.644	81
		Dobolu (Naryn)	KRM	26	22	5.778	0.020	-1.240	82
		Sary-Tash	KRS	46	33	5.879	0.020	-1.945	88
		Tamga	KRT	29	24	5.510	0.019	-1.701	81
	Shors	Kemerovo region	SHO	50	22	6.376	0.021	-0.946	80
	Sojots	Sojot	ST	31	15	4.692	0.016	-1.423	84
	Telenghits	Kokorya, Altai Republic	TLG	50	37	6.569	0.022	-1.986	CNRS database
	Teleuts	Kemerovo region	TEU	50	32	5.918	0.020	-1.587	80
	Todjins	Todja district, Tuva Republic	TD	48	26	5.340	0.018	-1.340	84
	Tofalars	Tofalar	TF	50	14	5.246	0.018	-0.532	84
	Tubulars	Ust'-Pyzha	TUB	50	16	6.011	0.020	-1.205	CNRS database
	Turkish	Eastern and western Azerbaijan	TIQ	40	32	4.524	0.015	-2.117	78
	Turkmen	Turkmenistan	TKQ	40	34	5.863	0.020	-1.990	78
		Ourguentch	TUR	50	36	5.262	0.018	-1.634	87
	Tuvans	Tuva	TV	50	29	7.202	0.024	-1.079	84
	Uighurs	Taldy-Corgan, Kazakhstan	UGC	50	40	5.454	0.018	-2.002	88
		Xinjang. China	UGY	45	38	5.688	0.019	-1.990	89
	Uzbeks	Novmetan, Bukhara	LUZ	46	26	5.610	0.019	-1.654	81
		Andijan, Uzbekistan	UZA	31	26	5.232	0.018	-1.635	81
		Kungrat (Noukous), Uzbekistan	UZB	40	33	5.387	0.018	-2.028	81
		Surkhandarya, Uzbekistan	UZQ	40	35	5.015	0.017	-2.019	78
		Urtoqqichloq, Tajikistan	UZT	40	24	5.047	0.017	-1.741	81
	Volga-Tatars (Kazan)	Aznakaevo, Russia	VTK	50	28	4.766	0.016	-1.491	90
	Volga-Tatars (Mishar)	Buinsk, Russia	VTM	50	36	4.716	0.016	-2.123	90
	Yakuts	Sakha, Yakutia Republic	YAK	30	15	4.618	0.016	-0.988	80
Volga-Finns	Moksha	Russia	MKS	21	15	4.419	0.015	-1.492	91

Supplementary Table 20. Genomic capture samples.

Information on the six Scythian individuals for which genome-wide capture data was obtained and the number of SNPs overlapping the Human Origins array for the samples only shotgun sequencing was performed (IS2 and Ze6).

Harvard ID	Mainz ID	Site	Culture/Label	Date	# SNPs	Sex
I0562	Be9	Berel', Kazakhstan	Pazyryk_IA	4th–3rd c. BC	549958	F
I0563	Be11	Berel', Kazakhstan	Pazyryk_IA	4th–3rd c. BC	420749	M
I0574	PR9	Pokrovka, Russia	EarlySarmatian_IA	5th–2nd c. BC	186890	F
I0575	PR3	Pokrovka, Russia	EarlySarmatian_IA	5th–2nd c. BC	306498	M
I0576	A17	Arzhan, Russia	AldyBel_IA	7th–6th c. BC	108952	F
I0577	A10	Arzhan, Russia	AldyBel_IA	7th–6th c. BC	427557	M
IS2	IS2	Ismailovo, Kazakhstan	ZevakinoChilikta_IA	9th–7th c. BC	74469	M
Ze6	Ze6	Zevakino, Kasakhstan	ZevakinoChilikta_IA	9th–7th c. BC	163338	F

Supplementary Table 21. Shotgun sequencing results.
Results of the shotgun sequencing and contamination estimate using mitochondrial DNA. Cov=coverage; Std=standard deviation.

sample	*Be9*	*Ze6*	*Is2*
raw reads	314014958	258245806	153461062
kept after quality filtering	244331550	242562332	142302734
endogenous DNA [%]	9.63	5.99	13.62
aligned reads	23529958	14522660	19378544
aligned pairs	11764979	7261330	9689272
aligned pairs without duplicates	11655425	10148169	3702997
Cov Ø	0.3	0.28	0.12
Std	0.64	0.77	0.4
mitochondrial contamination estimate			
mtDNA covered [%]	98.87	100	97.47
Sequencing depth	38.36 +/- 22.40	182.69 +/- 51.31	15.96 +/- 7.39
Estimated error rate	0.0079	0.0171	0.0083
Contamination estimate [%]	0.20–2.20	0.01–0.59	0.03–3.31
5' C to T transition	0.17	0.17	0.15

Supplementary Table 22. Y-chromosome haplogroups

ID	Y-haplogroup	Polymorphisms
I0563	R1a1a1b2	Z93:7552356G->A
I0575	R1b1a2a2	CTS1078:7186135G->C, S20902:18383837C->T
I0577	R1a1a1b	S441:7683058G->A
IS2	Q1a	F903:7014317G->C, M1168:22155597G->A

Supplementary Table 23. Testing whether (*Test*, Yamnaya_Samara) are descended from a single stream of ancestry in relation to outgroups Ust_Ishim, Kostenki14, MA1, Papuan, Onge. P-values greater than 0.05 are highlighted.

Test	**P-value for rank=0**
Pazyryk_IA	2.64E-78
Russia_IA	6.38E-63
Karasuk	4.27E-49
Zevakino_Chilikta_IA	7.24E-23
Russia_LBA	1.23E-22
Okunevo	8.52E-21
Aldy_Bel_IA	8.75E-11
Sintashta	2.48E-05
Srubnaya	3.09E-05
Early_Sarmatian_IA	1.18E-04
Andronovo	1.08E-03
Mezhovskaya	4.33E-03
Samara_IA	1.64E-02
Potapovka	6.85E-02
Poltavka	4.43E-01
Afanasievo	6.16E-01
Russia_EBA	7.02E-01
Yamnaya_Kalmykia	7.67E-01

Supplementary Table 24. Testing whether (*Test*, Yamnaya_Samara, LBK) are descended from two streams of ancestry in relation to outgroups Ust_Ishim, Kostenki14, MA1, Papuan, Onge. P-values greater than 0.05 are highlighted. The Mixture proportion of Yamnaya_Samara ancestry with its standard error is given. Population labels in bold are those which can be modelled as a mix of Yamnaya_Samara and LBK but could not be modelled as a simple clade with Yamnaya_Samara (Supplementary Table 23).

		**Yamnaya_Samara**	
Test	**P-value for rank=1**	**Proportion**	**s.e.**
Russia_IA	1.16E-61	-341.692	143.765
Pazyryk_IA	1.94E-58	-0.271	1.326
Karasuk	4.90E-50	0.705	0.399
Russia_LBA	5.13E-25	-111.131	22.167
Zevakino_Chilikta_IA	1.52E-23	0.782	0.347
Okunevo	1.45E-19	1.401	0.234
Aldy_Bel_IA	1.67E-09	0.723	0.098
Early_Sarmatian_IA	1.01E-02	0.686	0.090
**Mezhovskaya**	5.72E-02	0.764	0.082
**Sintashta**	1.15E-01	0.691	0.063
**Samara_IA**	2.68E-01	0.717	0.098
Poltavka	3.21E-01	0.968	0.063
**Andronovo**	3.73E-01	0.729	0.065
Afanasievo	4.46E-01	0.999	0.064
**Srubnaya**	5.61E-01	0.746	0.045
Russia_EBA	5.85E-01	1.075	0.174
Potapovka	6.01E-01	0.750	0.091
Yamnaya_Kalmykia	6.66E-01	1.028	0.060

Supplementary Table 25. Testing whether (*Test*, Yamnaya_Samara, Han) are descended from two streams of ancestry in relation to outgroups Ust_Ishim, Kostenki14, MA1, Papuan, Onge. P-values greater than 0.05 are highlighted. The Mixture proportion of Yamnaya_Samara ancestry with its standard error is given. Population labels in bold are those which can be modelled as a mix of Yamnaya_Samara and Han but could not be modelled as a simple clade with Yamnaya_Samara (Supplementary Table 23).

		**Yamnaya_Samara**	
Test	**P-value for rank=1**	**Proportion**	**s.e.**
Okunevo	7.06E-09	0.739	0.033
Sintashta	3.85E-05	0.957	0.023
Srubnaya	8.53E-04	0.950	0.016
Andronovo	9.62E-04	0.970	0.021
Russia_LBA	1.34E-02	0.535	0.042
**Samara_IA**	5.37E-02	0.934	0.031
**Karasuk**	9.77E-02	0.723	0.015
Potapovka	1.55E-01	0.943	0.030
**Russia_IA**	2.24E-01	0.612	0.018
**Aldy_Bel_IA**	2.47E-01	0.789	0.029
**Early_Sarmatian_IA**	2.55E-01	0.882	0.026
**Mezhovskaya**	3.11E-01	0.904	0.028
**Pazyryk_IA**	3.30E-01	0.491	0.022
Afanasievo	4.61E-01	0.994	0.022
Poltavka	4.67E-01	0.978	0.019
Russia_EBA	5.48E-01	1.011	0.046
**Zevakino_Chilikta_IA**	6.33E-01	0.622	0.033
Yamnaya_Kalmykia	7.00E-01	0.988	0.019

Supplementary Table 26. Testing whether (*Test*, Yamnaya_Samara, Nganasan) are descended from two streams of ancestry in relation to outgroups Ust_Ishim, Kostenki14, MA1, Papuan, Onge. P-values greater than 0.05 are highlighted. The Mixture proportion of Yamnaya_Samara ancestry with its standard error is given. Population labels in bold are those which can be modelled as a mix of Yamnaya_Samara and Nganasan but could not be modelled as a simple clade with Yamnaya_Samara (Supplementary Table 23).

		**Yamnaya_Samara**	
Test	**P-value for rank=1**	**Proportion**	**s.e.**
Okunevo	2.91E-07	0.653	0.041
Sintashta	2.13E-05	0.956	0.030
Srubnaya	3.54E-04	0.944	0.020
Andronovo	7.04E-04	0.970	0.027
Russia_LBA	1.70E-02	0.419	0.053
Samara_IA	4.02E-02	0.923	0.040
**Potapovka**	1.18E-01	0.936	0.038
**Early_Sarmatian_IA**	1.56E-01	0.857	0.033
**Aldy_Bel_IA**	1.85E-01	0.734	0.037
**Mezhovskaya**	2.32E-01	0.882	0.035
**Russia_IA**	4.03E-01	0.511	0.023
Poltavka	4.54E-01	0.974	0.025
Afanasievo	4.59E-01	0.992	0.027
**Pazyryk_IA**	5.36E-01	0.354	0.029
Russia_EBA	5.46E-01	1.012	0.057
**Karasuk**	6.06E-01	0.648	0.018
Yamnaya_Kalmykia	7.15E-01	0.984	0.023
**Zevakino_Chilikta_IA**	7.27E-01	0.521	0.041

Supplementary Table 27. Phenotypic results for genomic capture samples.
Allele counts for select SNPs of phenotypic effect assessed in capture data (Mainz sample IDs in parentheses). Anc=ancestral; Der=derived.

Gene	SNP	Anc/Der	East			West	
			I0562 (Be9)	I0563 (Be11)	I0576 (A17)	I0574 (PR9)	I0575 (PR3)
HERC2	rs12913832	A/G	2/7	5/8	0/0	1/0	1/0
SLC24A5	rs1426654	G/A	0/3	1/1	0/0	0/0	0/0
SLC45A2	rs16891982	C/G	24/0	7/7	1/0	1/0	0/4
TYR	rs1042602	C/A	8/7	11/0	0/0	1/0	1/0
LCT	rs4988235	C/T	17/0	11/0	0/0	0/0	1/0
NADSYN1	rs7940244	C/T	0/22	9/2	0/0	2/0	2/0
FADS1	rs174546	T/C	14/0	10/0	1/0	0/0	0/0
EDAR	rs3827760	A/G	2/2	0/0	0/0	0/0	1/0

Supplementary Table 28. Allele frequencies for phenotypic SNPs.

In A. all Scythians and B. eastern (48N) v. western (6N) Scythians. Modern frequencies taken from 1000 Genomes release 16 Oct 2014[92]. [1]Allele selected in Europeans; [2]Allele selected in Asians.

A.

**Gene**	**SNP**	**Anc>Der**	**Anc. Frequency**	**Der. Frequency**	**No. Alleles**	**Modern Der. Freq. EUR**	**Modern Der. Freq. ASN**
HERC2	rs12913832	A>G[1]	0.70	0.30	44	0.64	<0.01
SLC24A5	rs1426654	G>A[1]	0	1	4	~1	.01
SLC45A2	rs16891982	C>G[1]	0.39	0.61	28	0.94	0.01
TYR	rs1042602	C>A[1]	0.94	0.06	16	0.37	<0.01
LCTa	rs4988235	C>T[1]	0.97	0.03	68	0.51	0
LCTb	rs182549	G>A[1]	0.98	0.02	48	0.51	0
ADH1Ba	rs3811801	C>T[2]	0.97	0.03	38	0	0.51
ADH1Bb	rs1229984	G>A[2]	1	0	8	0.03	0.70
ABCB1a	rs1128503	C>T	0.69	0.31	16	0.42	0.63
ABCB1b	rs2032582	G/T[2]/A	0.72	0.25/0.03	36	.02/.41	.13/.40
ABCB1c	rs1045642	C>T[2]	0.45	0.55	44	0.52	0.40
ABCC11	rs17822931	C>T[2]	0.70	0.30	40	0.14	0.78

B.

**Gene**	**SNP**	**Anc>Der**	**Derived Frequency (2N)**	
			**EAST**	**WEST**
HERC2	rs12913832	A>G[1]	0.25 (40)	0.75 (4)
SLC24A5	rs1426654	G>A[1]	1 (4)	
SLC45A2	rs16891982	C>G[1]	0.58 (26)	1 (2)
TYR	rs1042602	C>A[1]	0.07 (14)	0 (2)
LCTa	rs4988235	C>T[1]	0.03 (62)	0 (6)
LCTb	rs182549	G>A[1]	0.02 (42)	0(6)
ADH1Ba	rs3811801	C>T[2]	0.03 (32)	0 (6)
ADH1Bb	rs1229984	G>A[2]	0 (8)	
ABCB1a	rs1128503	C>T	0.29 (14)	0.50 (2)
ABCB1b	rs2032582	G/T[2]/A	0.22T/0.03A (32)	0.5T (4)
ABCB1c	rs1045642	C>T[2]	0.53 (40)	0.75 (4)
ABCC11	rs17822931	C>T[2]	0.35 (34)	0 (6)

Supplementary Note

1) Approximate Bayesian Computation

Materials & Methods

Approximate Bayesian computation (*ABC*) is a flexible framework for making demographic inferences on molecular genetic data[1] and was used to explore the demographic history underlying the Scythian groups analysed in this study. Briefly, *ABC* compares summary statistics computed from empirical data (S^*) to statistics simulated (S) under various scenario and determines the simulations for which S is the closest to S^*. Using a proximity measure to select S sufficiently close to S^*, these summary statistics can then be used to evaluate the likelihood of different candidate scenarios[2,3]. The simulated model parameters are drawn from prior distributions and their association with the selected S allows their posterior distributions to be obtained. It leads to a quantification of the parameter probabilities of the investigated scenarios[1,4]. This approach has been successfully applied in genetic studies of human evolutionary history[5,6], including recent studies of ancient DNA[7-11]. Here, we utilized HVR1 mtDNA sequence data for populations associated with the Scythian culture to make inferences about their origins and relations to other ancient and contemporary populations. Specifically, we were interested in exploring four topics: 1) The genetic continuity during the Iron Age in eastern Scythian populations, specifically between Pazyryk and earlier Scythian cultures from the Altai region; 2) The relationship between eastern and western Scythian populations, with specific focus on the putative origins of the Scythian culture; 3) The relationship of Iron Age Scythians to nomadic groups (Andronovo/Fedorovo) from the preceding Bronze Age; and 4) The descent of Iron Age Scythians in contemporary Eurasian populations. Although these four analyses differed in the scenarios evaluated, their general methodology is similar and will hence be presented as one.

Demographic scenarios

Genetic continuity between the eastern Scythian groups.

Genetic continuity over the time period of interest (roughly from 1000–200 years BC) was evaluated by pooling available samples into two temporal groups: 26 individuals from the 9th–6th century BCE (labelled as ES69bc), and another 67 individuals from the 4th–3rd century BCE (labelled as ES34bc) (Tagar/Tes individuals were excluded due to their dating from the 8th century BCE to the 1st century CE). Assuming a generation

length of 25 years[12], these samples were on average taken at t = 94 generations before present (g BP) and t = 108 g BP, i.e. 14 generations apart. We evaluated the following scenarios that can explain their relationship (Supplementary Fig. 2): two samples from one growing, constant or bottlenecked population or two samples from (growing or constant) populations diverged in the more distant past. Effective population size priors were uniformly sampled from 100–1,000,000 individuals, whereas splitting time priors were uniformly sampled from 108–4,000 g BP (~ 2.7–100 ky BP). To assess potential bias due to the choice of this particular splitting time prior, an additional set of scenarios sampled splitting times from a reduced prior distribution (108 – 400 g BP). For scenarios with (exponential) population growth, rates of population size change per generation were uniformly sampled from 0–2% and growth was stopped after populations merged into the ancestral population. Scenarios with sudden population size changes introduced moderate (10%) and strong (1%) bottlenecks between the sampling times of the two groups (95–107 g BP), after which original population sizes were regained. See Supplementary Fig. 2 for full details on evaluated scenarios.

Relationship between western and eastern Scythian groups and their origins.

Following the evaluation of genetic continuity in eastern Scythian groups, we tested several hypotheses on the relationship with western Scythians. Moreover, we also included contemporary samples representative of genetic diversity on the extremes of Eurasia to further assess the geographic origins of Scythians. For the western Scythians, we combined 34 individual sequences into one sample group (labelled WS34bc), dated on average at t = 94 g BP. These western samples included some Early Sarmatians that may have a different demographic history from Scythians. However, analyses that excluded these individuals yielded very similar results, and hence they were included here to increase the sample size of the western group. Sequences from Bramanti et al. (2009)[13] and Tajima et al. (2004)[14] were taken to form representative samples (n=50) of contemporary western Europeans and (northern) Han Chinese, respectively. Briefly, we tested four different hypotheses of the origins of Scythians (Supplementary Fig. 4a): a western origin, an eastern origin (two variants), and a multiregional origin. In these scenarios, the Scythian groups split from western or eastern Eurasians at t = 200 g BP (~ 5 ky BP), after which they start to exchange migrants. Effective population sizes and growth rates were sampled from the same priors as before, and growth was stopped after all populations have been merged into the ancestral population at t = 1600 g BP (~ 40 ky BP). Migration rates were sampled uniformly from 0.001–0.01 individuals per generation. Under the preferred scenario, we evaluated gene flow patterns in more detail in additional analyses (Supplementary Fig. 4b), with the same priors on effective population sizes, growth rates and gene flow.

Relation of Bronze Age mobile groups to Scythians.

Thirdly, we evaluated whether Bronze Age mobile groups, specifically representative samples from the Andronovo/Fedorovo and Krotovo cultures, were ancestral to eastern or western Scythians. For this purpose, we combined available sequences: from individuals assigned to the Andronovo culture (n=9) from the Krasnoyarsk area[15] and to the Late Krotovo culture (n=20) and Andronovo culture (n=20) from the Baraba forest steppe in western Siberia[16] to form a representative sample (n=47) of Central Asian Bronze Age groups, dated on average at t = 151 g BP. The descent of this sample was tested by placing it onto the four available branches of the population tree at the time of its sampling (Supplementary Fig. 4c), with its own population dynamic parameters. All effective population size, growth rate and migration rate priors were sampled as specified before.

Relation of contemporary Eurasian populations to Iron Age Scythians.

Finally, we investigated the descendants of the Iron Age Scythian populations among contemporary human populations in Eurasia (Supplementary Fig. 7). We compiled a database consisting of representative samples (n=3410) from 86 contemporary populations distributed throughout Eurasia (Supplementary Table 19) and extended the preferred model for the relationship between western and eastern Scythians (see above) to include populations sampled at present. In our model selection procedure (Supplementary Fig. 7), contemporary populations could be placed on four different places in the demographic tree: they could have descended either directly from western Scythians or from eastern Scythians, or share a common ancestor in the more distant past with either of the Scythian groups. The simulation was parameterized as follows: model parameter priors for Scythian populations (effective population sizes, growth rates and migration rates) were sampled uniformly from posteriors of the preferred model. Next, the ability to discern between competing models depended strongly on splitting time; hence these (t_{12} and t_{34} in Supplementary Fig. 7) were sampled from t_{min} to 800 g BP. We used a model selection procedure on 3000 pseudo-observed, simulated sets of summary statistics to determine, for each split, the minimum splitting time (t_{min}), defined as the threshold with at least 90% statistical power to correctly identify a population not directly descended from the Scythian populations (Supplementary Fig. 8). This resulted in t_{min} = 350 g BP for the western split (t_{12} in Supplementary Fig. 7) and t_{min}=300 g for the eastern split (t_{34} in Supplementary Fig. 7). Thus, we had a higher statistical power to discern descent from the eastern Scythian groups, likely due to the presence of two ancient samples for comparison. The simulated candidate scenarios were also not compatible with contemporary populations that split from either Scythian population between 94 and 300 – 350 g BP (between about 2.5 and 7–8 ky BP), and we adopted a conservative approach to identifying those contemporary populations that were associated with each candidate demographic history (at least $p > 0.90$ posterior probability for the preferred scenario).

Mutation model and summary statistics

Gene genealogies for each demographic scenario were simulated 500,000 times under the coalescent in Bayesian Serial SimCoal[17,18]. Genetic data were simulated under a HKY model, assuming a gamma distribution of mutation rate heterogeneity across the sequence, with an average mutation rate (per year and per site) sampled from a uniform distribution between $4.0.10^{-8}$ and $4.0.10^{-7}$ [19], 56% invariable sites, a 0.9375 transition/transversion rate bias, a θ parameter of 0.3 and 10 discrete classes[20]. These aspects of the mutation model were inferred from the empirical genetic data using Jmodeltest v1.0[21]. For subsequent evaluations of Scythian origins, Bronze Age mobile groups and contemporary populations, simulations were performed with a mutation rate of 0.0025 mutations per locus per generation (but with a gamma distribution for mutation rate heterogeneity across the sequence) thought to be more appropriate for the HVR1 and time period of study[19,22].

Samples were drawn from each simulated genealogy, corresponding in timing and size to the empirical data (see above). For the analyses on Scythian descent in contemporary populations, where the contemporary empirical data (Supplementary Table 19) have varying sample sizes for each population, simulations were run at different sample sizes (S=30, S=40, S=50) for the contemporary population samples. The empirically observed data sets were then compared to simulated data with the closest sample sizes. We calculated four within-population summary statistics per sample: number of haplotypes (*Nh*), average number of pairwise differences between two sequences (*k*), nucleotide diversity (π) and Tajima's *D*. Between each pair of samples, we calculated F_{ST}[23] and the percentage of haplotypes shared between two population samples (*PHS*). These summary statistics were calculated on the empirical data using DNASp v.5.0[24].

Scenario selection, confidence in model choice and parameter estimation

First, we verified that simulated summary statistics adequately approximated the observed values (by inspecting their prior distributions). Then, we selected the 1.0% (5,000) or the 0.5% (2,500) simulations with S closest to S^* to calculate posterior probabilities of each demographic scenario, using a polychotomous logistic regression[25] or a non-linear regression method based on neural networks[26], after correcting the data for heteroscedasticity. Both these methods have considerably better statistical performance than the rejection method based on a proximity criterion[25]. The non-linear regression method is preferred when many summary statistics are used, such as was the case in our analyses[26].

Given the importance of evaluating the model choice procedure in *ABC*[27], we quantified model choice confidence through a leave-one-out cross-validation analysis. Here a simulation under any demographic scenario was randomly selected and its summary statistics were then used as pseudo-observed values, and the scenario choice procedure was repeated with all the other simulations, performed 100 times per scenario simulated. For each demographic scenario, error I rates were calculated as the percentage of misidentified simulations (when the demographic scenarios is true), and error II rates were calculated as the percentage of times a different scenario was incorrectly identified as the scenario under question.

Posterior distributions of the model parameters of the preferred scenario were calculated through a non-linear regression using neural networks[26] on the 0.5% simulations with S closest to S^*, after applying a logit transformation to the parameter values to improve fit[1]. To further check the robustness of our analysis, 10,000 post-hoc sets of summary statistics were simulated under model parameters drawn from their posterior distributions, and these distributions were compared to observed values. All analyses were performed using the package *abc*[28] in R v.2.15.1[29].

Genetic continuity between Iron Age Scythian groups:

Our simulation settings successfully reproduced summary statistics that were similar to those observed on the empirical data (Supplementary Fig. 3), and 10 summary statistics were used for model selection. These analyses reveal the highest support for a demographic scenario where the two eastern Scythian sample groups were derived from one single population, expanding over the time period considered (Supplementary Table 9). This scenario (scenario 6, Supplementary Fig. 2) was highly supported in our model selection procedure (tolerance = 0.5%, logistic regression, p=0.995, neural networks method p=0.568). Other models where the eastern Scythian samples were also derived from one temporally continuous population also received relatively high support. Importantly, scenarios that assumed that these eastern Scythian groups were derived from two previously diverged populations received very little support (cumulative posterior probabilities logistic regression p=0.000, neural networks method p=0.009). This result remained unchanged when splitting times were sampled from a narrower prior (108–400 g BP) (cumulative posterior probabilities logistic regression p=0.008, neural networks method p=0.001). Moreover, low type I (2.1 %) and type II (3.3%) error rates support a high power of the model selection procedure to uncover the most likely scenario (Supplementary Table 10). Estimation of the posteriors of the model parameters for the preferred scenario suggests that the eastern Scythian population was undergoing expansion during the first millennium BC. Hence the genetic data used in these analyses indeed contain information on the underlying demographic history (Supplementary Table

11). Finally, post-hoc simulations confirm that the uncovered demographic model was able to reproduce the genetic diversity patterns empirically observed (Supplementary Fig. 3).

A multiregional origin of Iron Age Scythians:

As before, simulation settings were successfully able to reproduce summary statistics similar to those observed on the empirical data (Supplementary Fig. 5), and 35 summary statistics were subsequently used for model selection. The model selection procedure revealed that a multiregional model of Scythian origins received the highest support for the empirically observed genetic diversity patterns (Supplementary Table 12, 0.5% closest simulations, posterior probability p=0.701 for logistic regression, p=0.715 for neural networks method), while a model of western origin also received some support (Supplementary Table 12, 0.5% closest simulations, posterior probability p=0.286 for logistic regression, p=0.267 for neural networks method). These inferences become more pronounced when repeating analyses with only the western and multiregional candidate models (multiregional model; 0.5% closest simulations, posterior probability p=0.917 for logistic regression, p=0.929 for neural networks method), and furthermore show that model choice is independent of tolerance rate (Supplementary Figure 5b). When computing Bayes factors between those two scenarios, support for the multiregional model ranges from 'substantial' to 'strong' for the logistic regression method, and from 'weak' to 'substantial' for the neural networks method, depending on the tolerance rate (Supplementary Figure 5b). An evaluation of confidence in the model selection procedure revealed low overall error I (3.3 %) and error II (1%) rates (Supplementary Table 14), confirming that the model selection procedure had a high power to discern scenarios and uncover the most likely one. Focusing on the two best fitting models, a western origins model had 3% chance of being mistaken for a multiregional model; *vice versa* a multiregional model had a 9% chance of being incorrectly identified as a western model. Still, model selection under ABC has been the topic of considerable debate (e.g.[30]) and we therefore repeated analyses using a novel approach based on random forests[31], which confirmed that the multiregional model has the highest support (model posterior 0.672 in a two-way analysis based on a sample of 5.10^4 and a regression forest of 500 trees, in the R-package *abcrf*). Together these analyses show that while a western origin model can also explain the observed genetic diversity patterns and can indeed not be completely discounted, a multiregional origin model has higher statistical support, is discernable from a western model, and is thus considered the preferred model.

Based on the model parameter posteriors, the preferred demographic model can be described as follows: the western and eastern Scythian groups arose independently, perhaps in their respective geographic regions, and thereafter experience significant population expansions (during the 1st millennium BCE). Importantly, gene flow between

the Iron Age Scythian groups was ongoing and substantial, with asymmetrical gene flow from western to eastern groups, rather than vice versa (see Supplementary Table 15 for details). A more detailed evaluation of gene flow patterns under the preferred demographic scenario did not change the inferences made above, as neither of these alternative scenarios of gene flow provided a better fit to the observed genetic data (Supplementary Table 13). A post-hoc evaluation confirms the ability of this demographic model to reproduce the genetic diversity patterns observed in the empirical data (Supplementary Fig. 5).

Bronze Age mobile groups linked to the Andronovo culture are ancestral to eastern Scythians:

An inspection of the prior distributions of the 54 summary statistics available for this analysis confirms a good fit to the observed genetic diversity patterns (Supplementary Fig. 6, grey lines). A subset of 14 of these summary statistics (within-population and between-population summary statistics involving the Bronze Age sample) was used for model selection procedures. The model selection procedure (Supplementary Table 16) uncovered a strong support for Bronze Age mobile groups being ancestral to the eastern Scythian branch (0.5% threshold, p=0.986 logistic regression, p=0.991 neural networks method). Moreover, an evaluation of the confidence in model choice (Supplementary Table 17) showed that this scenario was characterized by low error I (4.0%) and error II (2.3%) rates, suggesting high power of the procedure to identify the best scenario. All 54 summary statistics were then used to estimate posteriors, but an inspection of the retained summary statistic values for this analysis (Supplementary Fig. 6, red lines) revealed that some of these provided a relatively poor fit to the observed data. Specifically, the simulations appeared to somewhat overestimate the number of haplotypes (*Nh*) and underestimate Tajima's *D* within the Bronze Age sample. Post-hoc simulated summary statistics values (Supplementary Fig. 6, dotted black lines) however provided a better fit. Therefore, while these analyses strongly imply that Iron Age Scythians from the East have descended from earlier mobile groups from the Bronze Age, and that the Scythian culture may have hence emerged first in the East, the scenario considered here may not accurately capture the full complexity of the underlying demographic history of groups maintaining a nomadic way of life in Eurasia during the Bronze and Iron Ages.

Contemporary descent from western Iron Age Scythians is mainly found among various Eurasian groups, whereas contemporary descent from eastern Iron Age Scythians is almost exclusively Turkic:

We used 10 summary statistics for our model selection procedure (within-population statistics for each contemporary population, and F_{ST} and *PHS* between contemporary population and each Scythian sample group). An inspection of simulated values suggested that these were successful in approximating the observed values, regardless of

the sample size of simulated contemporary populations (Principal Components Analysis in Supplementary Fig. 9). An evaluation of confidence in model choice (Supplementary Table 18) suggested a generally high power to differentiate between the four scenarios, with low error I (ranging from 1.1% to 3.3%) and error II (ranging from 1.1 to 3.7%) rates.

We then applied this model selection procedure to 86 contemporary population samples and the main findings can be summarized as follows (Supplementary Fig. 10 and 11). Firstly, contemporary populations likely to be directly descended from western Scythians were mainly found in geographic proximity to the archaeological sites, consisting of Indo-European, Iranian, Slavic and Caucasian groups, but also included some Uzbeks (Supplementary Fig. 10a and Supplementary Fig. 11). The populations with the highest likelihood of direct descent were either located in close proximity (e.g. Russians, Mohska), the Caucasus (e,g, Azeris, Abazinians) or in Central Asia (e.g. some Uzbeks, Tajiks) (Supplementary Fig. 10a). Secondly and similarly, contemporary populations most likely to share a common ancestor with western Scythians were primarily found among Iranian and Caucasian groups, predominantly situated in the western part of our sampling range (Supplementary Fig. 10c and Supplementary Fig. 11). Though supported by lower model posteriors, these included Iranians, Chechens, Cirkassians and also (again) Uzbeks. Thirdly, contemporary populations with the highest likelihood of being directly descended from eastern Scythian groups are almost exclusively Turkic language speakers (Supplementary Fig. 10b). Particularly high statistical support was documented for some Turkic speaking groups geographically located close to the archaeological sites of the eastern Scythians (e.g. Telenghits, Tubular, Tofalar), but also among Turkic speaking populations located in Central Asia (e.g. Kyrgyz, Kazakhs and Karakalpaks) (Supplementary Fig. 11). These same results were found for some Turkic groups located even further to the West, such as the Kazan Volga-Tatars. Finally, contemporary populations likely to share a common ancestor with eastern Scythians were mainly found among Turkic, Mongolian and Siberian groups located in eastern Eurasia (Supplementary Fig. 10d and Supplementary Fig. 11). In summary, these results provide further support for a multi-regional origin of the various Scythian groups from the Iron Age.

2) Genomic capture analysis of ancient Scythians

We used in-solution hybridization[32] to capture data from six individuals (Early Sarmatian, Aldy Bel, Pazyryk) on a target set of 1,233,553 SNPs[33]. We also analysed shotgun data from two individuals of the Zevakino Chilikta culture. All eight newly reported Iron Age (IA) individuals are listed in Table 1 and Supplementary Table 20 with the number of SNPs overlapping the Human Origins[34,35] array (out of a total of 592,1462) that were used for subsequent analyses.

Principal components analysis

We performed principal components analysis (PCA)[36] of 777 present-day West Eurasians[33,34,37] on which we projected the eight newly reported samples as well as 167 other samples from Europe, the Caucasus, and Siberia from the literature[33,38,39] (Fig. 4). The two Sarmatian samples from Europe cluster with an Iron Age sample from the Samara district[33] and are generally close to the Early Bronze Age Yamnaya samples from Samara[33,37] and Kalmykia[39] and the Middle Bronze Age Poltavka samples from Samara[33]. The samples from Pazyryk, Aldy Bel, and Zevakino Chilikta are part of a loose cluster with other samples from Inner Asia[39], including Okunevo, Late Bronze Age and Iron Age Russia, and Karasuk[39]. These samples contrast with earlier samples of the Eurasian steppe belonging to the Andronovo, Sintashta[39] and Srubnaya[33] who overlap Late Neolithic/Bronze Age individuals from mainland Europe[33,34] and are shifted 'southwards' in PCA, towards the early farmers of Europe and Anatolia[33].

The PCA of West Eurasia does not allow one to examine the relationship of the ancient samples to world populations, so we also carried out principal components analysis of all 2,345 individuals of the Human Origins dataset[34] in which we also projected the ancient individuals (Fig. 5), which makes evident that the Iron Age samples we analysed are arrayed in their ancestry between present-day West Eurasians and eastern non-Africans.

f-statistics

Using *f*4-statistics we can see both effects (Fig. 6). We plot *f*4(*Test*, LBK; EHG, Mbuti) against. *f*4(*Test*, LBK; Han, Mbuti). This presents the form of a V-shape, with Yamnaya at the apex. The first of these statistics is zero for the part of the ancestry of *Test* that forms a clade with LBK. Thus, there are some populations with LBK-related ancestry (e.g., present-day Europeans and Sintashta) who are shifted relative to Yamnaya indicating that they have such ancestry. The second of these statistics is positive for populations that have Han-related ancestry. Iron Age groups are arrayed along cline from

Yamnaya to Ami, consistent with having ancestry from these two groups. We also computed statistics of the form $f3$(*Test*; Yamnaya_Samara, Han) which test whether a *Test* population has intermediate allele frequencies between Yamnaya_Samara and Han, which can only occur if it is a mixture of populations related to these two sources[33]. These statistics are significantly negative for the Iron Age groups, proving admixture (Supplementary Fig. 13).

ADMIXTURE analysis

We carried out ADMIXTURE analysis[40,41] of 2,345 present-day humans[34] genotyped on the Human Origins array[34,35] and 175 ancient individuals on a set of 296,340 SNPs after linkage disequilibrium pruning in PLINK[42,43] with parameters –indep-pairwise 200 25 0.4, varying K, the number of ancestral populations from 2 to 15. Ten replicates with different random seeds were performed, and the best replicate (highest log likelihood) was retained for each value of K.

We show the results for the ancient individuals in Fig. 7 and the complete analysis in Supplementary Fig. 14. Early farmers from Europe and Anatolia[33] belong primarily to an 'orange' component, but with some ancestry from the 'blue' component dominating the previous hunter-gatherers from Europe. A third, 'green' component is maximized in the Caucasus hunter-gatherers from Georgia[38]. All steppe populations have ancestry from both the blue and green components. A subset of them (including Srubnaya, Sintashta, and Andronovo), also have ancestry from the orange (early farmer component), while a different subset (including all Iron Age samples), also have ancestry from a different 'light blue' component that is maximized in the Nganasan (Samoyedic people from north Siberian), and is pervasive across diverse present-day people from Siberia and Central Asia. At lower values of K, the Iron Age samples have ancestry from ancestral components that are maximized in East Eurasian populations, a type of ancestry that occurs at trace levels, if at all, among earlier steppe inhabitants, consistent with the observations from PCA and f-statistics about this type of admixture.

Y chromosomes

We determined the sex of the eight individuals by examining the ratio of reads aligning to the X and Y chromosomes[44]. We determined the Y-chromosome haplogroup of four male individuals using the nomenclature of the International Society of Genetic Genealogy (www.isogg.org).

Individual I0563 (Pazyryk) belonged to the Z93 clade[45] which is frequent in Central Asia[45,46] and was also recorded in Bronze Age individuals from Mongolia[47] and the

Sintashta culture from Samara[33]. Individual I0577 (Aldy Bel) also belonged to haplogroup R1a1a1b but could not be determined more downstream. Individual I0575 (Sarmatian) belonged to haplogroup R1b1a2a2, and was thus related to the dominant Y-chromosome lineage of the Yamnaya (Pit Grave) males from Samara[37] (~3000BCE). Individual IS2 belonged to haplogroup Q1a which was also found in the Eneolithic period in Samara[33] in Europe but is most commonly found in present-day people from Siberia and the Americas[48] (Supplementary Table 22).

Modelling ancient steppe populations

It is now known that the Early Bronze Age population of the Yamnaya culture in the Eurasian steppe was a mix of the previous Eastern European hunter-gatherers (EHG) and a population of southern origin[37]. This process of admixture had begun by the Eneolithic period[49], and the resulting mix persisted down to at least the Middle Bronze Age Poltavka culture[33]. The Yamnaya from Samara[37] and Kalmykia[39] were very similar[33] to each other and to the people belonging to the Afanasievo culture[39] in Siberia. It is also known that the southern element[37] in the composition of this population had considerable antiquity as it may correspond to the Caucasus Hunter-Gatherers (CHG) from Georgia[38] who lived during Epipaleolithic to Mesolithic times.

The migration of southern population into the steppe was followed during the Middle to Late Bronze Age by migration of populations related to the early European farmers of mainland Europe into both the European part of the steppe (represented by the Srubnaya Late Bronze Age culture[33]) and further east into the Asian part of the steppe (represented by the Sintashta and Andronovo cultures[39]). By contrast, populations of later periods also exhibited admixture from the opposite end of the steppe, having ancestry related to East Asians during such cultures as Karasuk[39]. Steppe populations of the Bronze Age period migrated massively into mainland Europe[37] and are tenuously linked on the basis of the Y-chromosome to South Asia[33]. Thus, the steppe was a conduit of population movement, its populations influencing and being influenced by the surrounding settled agriculturalists of Europe, the Caucasus, and East Asia.

We attempted to model steppe populations as mixtures of the Early Bronze Age Yamnaya, who were the first truly mobile population on the steppe, spreading both westward[37] and eastward[37,39] across vast distances and the agriculturalists of Europe (represented by the Linearbandkeramik (LBK) farmers from central Europe, who were very similar to other European farmers[34,37]) or East Asia (represented by the Han Chinese in the absence of ancient samples from the region). We used the method of *qpWave/qpAdm*[37] which relates a set of Left populations to a set of Right ones, provides a statistical test for the number of streams of ancestry into the Left populations from the Right ones, and allows one to estimate mixture proportions. These programs were run

with the *allsnps*: YES mode, which uses all SNPs available for each quadruple of populations of an f4-statistic, rather than the intersection of all SNPs available across all Left and Right populations.

In our application, we set Right=(Ust_Ishim[50], Kostenki14[51], MA1[52], Papuan, Onge). This set includes Upper Paleolithic Eurasians and eastern non-Africans and can be used to differentiate between West and East Eurasian ancestry in the studied populations.

We first set Left=(*Test*, Yamnaya_Samara), thus testing whether *Test* and the Yamnaya from Samara could be descended from a single stream of ancestry from the Right set. Results are shown in Supplementary Table 23 and show that other Early and Middle Bronze Age populations from the Eurasian steppe are consistent with this hypothesis, while all the others are not descended from a single stream of ancestry with the Yamnaya from Samara.

Next, we set Left=(*Test*, Yamnaya_Samara, LBK), thus testing whether *Test*, the Yamnaya from Samara and the LBK farmers from central Europe could be descended from two streams of ancestry, in which case *Test* could potentially be modelled as a mixture of the other two populations. Indeed, we find that several populations that could not be modelled without LBK ancestry, can now be successfully modelled (Supplementary Table 24). Note that populations that could be modelled without LBK ancestry (Supplementary Table 23), are now shown to have Yamnaya_Samara ancestry not significantly different from 100%, while a number of new populations such as Srubnaya, Sintashta, Andronovo, Karasuk and others have less than 100% of their ancestry from the Yamnaya and the remainder from a population related to the early farmers of Europe, consistent with the 'southern' shift of these populations in the West Eurasian PCA (Fig. 4).

Nonetheless, several populations cannot be modelled as mixtures of the Yamnaya and the LBK, so we consider an alternative model in which we treat them as a mix of Yamnaya and the Han (Supplementary Table 25). This model fits all the Iron Age Scythian groups, consistent with them having ancestry related to East Asians not found in the other populations. The Early Sarmatians have minimal such ancestry (~10%), while the Pazyryk maximal (~50%). The other two groups (Aldy Bel and Zevakino-Chilikta) are intermediate (~20–40%). Note, however, that the two individuals from each of these groups are apparently heterogeneous (Fig. 4, 5). The Iron Age Scythian groups can also be modelled as a mix of Yamnaya and the Nganasan (Supplementary Table 26).

3) Shotgun analyses

We generated shotgun data for three Iron Age samples from eastern Central Asia: All samples originate from archaeological sites situated in East Kazakhstan: Ze6 and Is2 of the Zevakino-Chilikta culture, dating to the 9th–7th century BCE, and Be9 of the Pazyryk culture, dating to the 4th–3rd century BCE.

Sample preparation for shotgun sequencing

DNA extractions were performed as previously described, with the following exceptions: the volume of EDTA was increased to 6.7ml and the incubation time was prolonged to 48 hours.

Libraries were prepared as described in Kircher & Meyer 2012[53] including all adapter and primer sequences with slight modifications:
No use of USER enzyme for removing uracil in blunt-end reaction,
Final concentration of Adapter-Mix was 1.25µM per sample during ligation and
The first amplification of the libraries was performed in three parallels using AmpliTaq Gold® DNA polymerase
Library blank controls were carried through the entire library protocols and subsequently checked with Qubit fluorometric quantification and Agilent Bioanalyzer measurement.

All libraries were prepared out of 50µL DNA extract each. Library products were amplified in three parallels in a total reaction volume of 50µL consisting of 10µL Library product, UV-HPLC-H_2O, AmpliTaq Gold® DNA polymerase (0.05U/µL), 1xGold Buffer, 2.5mM $MgCl_2$ (Applied Biosystems, Life technologies, Darmstadt, Germany), 200µM of each dNTP (Qiagen, Hilden, Germany), and 200nM upper and lower primer (specified below). Cycling conditions were as follows: initial denaturation at 94 °C for 6 min followed by 12 (Ze6 and Be9) or 14 (Is2) cycles of denaturation at 94 °C for 40 sec, annealing at 60 °C for 40 sec and elongation at 72 °C for 40 sec with one additional final elongation step at 72 °C for 5 min.

Libraries Ze6
Six libraries were prepared in parallel with primer Is4 (200nM) and a sample specific 5' tailed indexing-primer (200nM) for the first amplification step (12 cycles).
PCR products of each library were combined, purified using the Qiagen MinElute purification kit and eluted in 22µL EB.
A second PCR was performed in three parallels with 7µL indexed library using Herculase II Fusion DNA Polymerase in a reaction volume of 100µL with 63µL UV-HPLC-H_2O,

20µL 5× Herculase II Reaction Buffer, 250 mM dNTPs each, Primer Is5 and Is6 (400nM each). Cycling conditions: inactivation step at 95 °C for 3 min followed by 10 cycles of denaturation at 95 °C for 30 sec, annealing at 60 °C for 30 sec and elongation at 72 °C for 30 sec followed by a final elongation step at 72 °C for 5 min.
PCR products were combined, purified using the Qiagen MinElute purification kit and eluted in 58µL EB.
For a shotgun sample pool between 10–20µL from every library were mixed to a final concentration of 3.65µg and gradually diluted for sequencing.
Since primer Is4 has not incorporated a specific index sequence a 7bp section of the 5'tailed primer Is4 has been read as individual index for correct assignment of raw reads to the sample after sequencing.

Libraries Be9
Five separate libraries were prepared with 50µL extract each as target.
The first amplification step was performed as described for Ze6, with the exception that primer Is7 was used instead of Is4. PCR products were purified with Invisorb MSB® Spin PCRapace kit. Parallels from one library were combined and eluted in 33µL EB.
Reamplification was performed using Herculase II Fusion DNA Polymerase with the same reaction set up as for sample Ze6; except that primer Is6 and Is7 (300nM each) were used. All amplified libraries were purified with Invisorb MSB® Spin PCRapace kit.
A third PCR was performed to add a second index on the short P5 site of the adapter sequence: 5µL from each library were taken and mixed before dividing it to three aliquots for PCR reaction with Herculase II Fusion DNA Polymerase and 10 cycles as already described; primer Is6 and a specific indexing-primer were used (300nM each).

Library Is2
For this sample only one library was prepared. The first amplification step was performed as described above except that P5 and P7 index primers were already used for the first amplification step (14 cycles).

Sequencing

Sequencing was performed on the Illumina HiSeq 2000 of the sequencing facilities of the faculty of Biology at the Johannes Gutenberg University, Mainz, Germany. Samples were sequenced as paired-end runs with a single sample per lane. The read length was 100bp.

Sequence data processing

Sequences were trimmed using KeyAdapterTrimFastQ_cc[54] with default parameters and disabling chimeric sequences as well as key filtering. Trimmed sequences were then filtered with QualityFilterFastQ_cc[54]. Reads were removed if 5 of the bases have quality of <15. Mate pairs were searched and separated from reads with no partner using an in-house python script. The mate pairs were then overlapped and merged into one single sequence using fastq-join from ea-utils with default parameters[55].
The resulting merged reads as well as the unmerged read pairs were aligned against the human reference genome (GRCh37/hg19) with bwa using default parameters[56]. Mapped reads are filtered for mapping quality 25 and for the proper pair property in case of unmerged reads[57]. Duplicates are removed using MarkDuplicates from picard-tools[58]. After duplicate removal sequences around indels were realigned and base qualities recalibrated using the Genome Analysis Toolkit with the recommended parameters and references[59] (Supplementary Table 21).

Variant Detection

For the determination of variant sites the Haplotype caller from the GATK Package Version 3.2[59] was used with the emit reference confidence option set to base-pair resolution. A file with all variations from the HapMap Project[60] and the Human Diversity Panel[61] that fell in the range of the covered regions of the alignments was provided as target sites. For downstream analysis detected sites with a genotype-quality below 15 were discarded.

Damage patterns and mitochondrial contamination

For each of the three samples used in shotgun sequencing, damage patterns were analysed using mapdamage 2.0[62] (Supplementary Fig. 12 and Supplementary Table 21). Mitochondrial contamination and sequencing error was estimated with a Bayesian approach, described in[50].

4) Phenotypic SNPs

We examined the state of SNPs having phenotypic impact or inferred to be under selection in ancient Europeans in five of the Scythians for whom capture data was generated[49] (Supplementary Table 27). We also examined a suite of 20 phenotypic markers in 52 Scythian individuals, prepared using single- and multiplex amplification, Sanger, and 454 sequencing and accepting only those genotypes replicated at least 3x. This dataset includes two Pazyryk individuals from the Berel' site for whom capture data was also produced (Supplementary Table 20, Supplementary Table 28), and excludes three potentially related individuals from the site of Barsucij Log. We used the 52-individual dataset to estimate allele frequencies for 24 eastern (48N) and 3 western (6N) Scythians (Supplementary Table 28). Concordance between the 5-individual capture dataset and the 52-individual dataset was 100% for shared positions.

Focusing on SNPs showing evidence of selection in either modern European or Asian populations, or having relatively high F_{ST} between these continental groups, we find evidence that the ancient Scythian population may have exhibited a mosaic of European and Asian-associated genotypes (Supplementary Table 28). In the capture data, we find a presence of the derived allele at rs3827760 in *EDAR* in a Pazyryk individual, consistent with the presence of East Eurasian admixture in this population. We do not find any derived alleles for rs4988235 in the *LCT* gene. Both ancestral and derived alleles were present in SNPs in four genes affecting pigmentation (*HERC2*, *SLC24A5*, *SLC45A2*, and *TYR*).

In the 52-individual dataset, we observe derived (reduced-pigmentation) alleles at pigmentation markers in *HERC2*, *SLC24A5*, and *SLC45A2* in both eastern and western Scythians, including individuals who are homozygous for the derived alleles at least for one of these markers. The derived alleles at these loci are under selection in Europeans[63-65]. At the two loci associated with lactase persistence, the derived allele is observed at very low frequency (~3%) and only in heterozygotes. The Scythians in this dataset are nearly fixed for the ancestral allele at *ADH1B* rs3811801 and fixed for the ancestral allele at *ADH1B* rs1229984, which are the European-associated states of these markers. One copy of the derived allele at rs3811801 was observed in the eastern population. The derived alleles at rs3811801 and rs1229984 confer some resistance to alcoholism and are under selection in East Asians[66-68]. The three putatively-related individuals are all carriers of the *HERC2* derived allele (two homozygotes and one heterozygote), are all heterozygous at the rs4988235 *LCTa* locus, and are homozygous for the derived allele at *SLC45A2*.

References

1 Beaumont, M. A., Zhang, W. & Balding, D. J. Approximate Bayesian computation in population genetics. *Genetics* **162**, 2025-2035 (2002).

2 Bertorelle, G., Benazzo, A. & Mona, S. ABC as a flexible framework to estimate demography over space and time: some cons, many pros. *Mol Ecol* **19**, 2609-2625 (2010).

3 Csillery, K., Blum, M. G., Gaggiotti, O. E. & Francois, O. Approximate Bayesian Computation (ABC) in practice. *Trends Ecol Evol* **25**, 410-418 (2010).

4 Tavare, S., Balding, D. J., Griffiths, R. C. & Donnelly, P. Inferring coalescence times from DNA sequence data. *Genetics* **145**, 505-518 (1997).

5 Verdu, P. *et al.* Origins and genetic diversity of pygmy hunter-gatherers from Western Central Africa. *Curr Biol* **19**, 312-318 (2009).

6 Laval, G., Patin, E., Barreiro, L. B. & Quintana-Murci, L. Formulating a historical and demographic model of recent human evolution based on resequencing data from noncoding regions. *PLoS One* **5**, e10284 (2010).

7 Ghirotto, S. *et al.* Inferring genealogical processes from patterns of Bronze-Age and modern DNA variation in Sardinia. *Molecular Biology and Evolution* **27**, 875-886 (2010).

8 Haak, W. *et al.* Ancient DNA from European early neolithic farmers reveals their near eastern affinities. *PLoS Biology* **8**, e1000536 (2010).

9 Der Sarkissian, C. *et al.* Ancient DNA reveals prehistoric gene-flow from siberia in the complex human population history of north East europe. *PLoS genetics* **9**, e1003296 (2013).

10 Bollongino, R. *et al.* 2000 years of parallel societies in Stone Age Central Europe. *Science* **342**, 479-481 (2013).

11 Fehren-Schmitz, L. *et al.* Climate change underlies global demographic, genetic, and cultural transitions in pre-Columbian southern Peru. *Proc Natl Acad Sci U S A* **111**, 9443-9448 (2014).

12 Fenner, J. N. Cross-cultural estimation of the human generation interval for use in genetics-based population divergence studies. *Am J Phys Anthropol* **128**, 415-423 (2005).

13 Bramanti, B. *et al.* Genetic discontinuity between local hunter-gatherers and central Europe's first farmers. *Science* **326**, 137-140 (2009).

14 Tajima, A. *et al.* Genetic origins of the Ainu inferred from combined DNA analyses of maternal and paternal lineages. *J Hum Genet* **49**, 187-193 (2004).

15 Keyser, C. *et al.* Ancient DNA provides new insights into the history of south Siberian Kurgan people. *Hum Genet* **126**, 395-410 (2009).

16 Molodin, V. I. *et al.* in *Population Dynamics in Prehistory and Early History - New Approaches Using Stable Isotopes and Genetics* (eds. Burger, J., Kaiser, E., Schier, W.) 93-112 (De Gruyter, 2012).

17 Excoffier, L., Novembre, J. & Schneider, S. SIMCOAL: a general coalescent program for the simulation of molecular data in interconnected populations with arbitrary demography. *The Journal of heredity* **91**, 506-509 (2000).

18 Anderson, C. N., Ramakrishnan, U., Chan, Y. L. & Hadly, E. A. Serial SimCoal: a population genetics model for data from multiple populations and points in time. *Bioinformatics* **21**, 1733-1734 (2005).

19 Endicott, P., Ho, S. Y. W., Metspalu, M. & Stringer, C. Evaluating the mitochondrial timescale of human evolution. *TRENDS IN ECOLOGY & EVOLUTION* **24**, 515–521 (2009).

20 Chaix, R., Austerlitz, F., Hegay, T., Quintana-Murci, L. & Heyer, E. Genetic traces of east-to-west human expansion waves in Eurasia. *Am J Phys Anthropol* **136**, 309-317 (2008).
21 Posada, D. jModelTest: phylogenetic model averaging. *Mol Biol Evol* **25**, 1253-1256 (2008).
22 Henn, B. M., Gignoux, C. R., Feldman, M. W. & Mountain, J. L. Characterizing the time dependency of human mitochondrial DNA mutation rate estimates. *Mol Biol Evol* **26**, 217-230 (2009).
23 Hudson, R. R., Slatkin, M. & Maddison, W. P. Estimation of levels of gene flow from DNA sequence data. *Genetics* **132**, 583-589 (1992).
24 Librado, P. & Rozas, J. DnaSP v5: a software for comprehensive analysis of DNA polymorphism data. *Bioinformatics* **25**, 1451-1452 (2009).
25 Beaumont, M. in *Simulations, Genetics and Human Prehistory McDonald Institute Monographs* (ed S.; Forster Matsumura, P.; Renfrew, C.) 208 (McDonald Institute for Archaeological Researche, 2008).
26 Blum, M. G. B. & Francois, O. Non-linear regression models for Approximate Bayesian Computation. *Stat Comput* **20**, 63-73 (2010).
27 Robert, C. P., Cornuet, J. M., Marin, J. M. & Pillai, N. S. Lack of confidence in approximate Bayesian computation model choice. *Proc Natl Acad Sci U S A* **108**, 15112-15117 (2011).
28 Csillery, K., Francois, O. & Blum, M. G. B. abc: an R package for approximate Bayesian computation (ABC). *Methods Ecol Evol* **3**, 475-479, doi:DOI 10.1111/j.2041-210X.2011.00179.x (2012).
29 R_Core_Team. *R: A language and environment for statistical computing.*, <http://www.R-project.org/> (2013).
30 Robert, C. P., Cornuet, J. M., Marin, J. M. & Pillai, N. S. Lack of confidence in approximate Bayesian computation model choice. *Proc Natl Acad Sci U S A* **108**, 15112-15117, (2011).
31 Pudlo, P. et al. Reliable ABC model choice via random forests. *Bioinformatics* **32**, 859-866, (2016).
32 Fu, Q. *et al.* DNA analysis of an early modern human from Tianyuan Cave, China. *Proc. Natl. Acad. Sci. USA* **110**, 2223–2227, (2013).
33 Mathieson, I. *et al.* Genome-wide patterns of selection in 230 ancient Eurasians. *Nature* **528**, 499-503, (2015).
34 Lazaridis, I. *et al.* Ancient human genomes suggest three ancestral populations for present-day Europeans. *Nature* **513**, 409-413, (2014).
35 Patterson, N. *et al.* Ancient admixture in human history. *Genetics* **192**, 1065-1093, (2012).
36 Patterson, N., Price, A. L. & Reich, D. Population structure and eigenanalysis. *PLoS Genet.* **2**, e190, (2006).
37 Haak, W. *et al.* Massive migration from the steppe was a source for Indo-European languages in Europe. *Nature* **522**, 207-211, (2015).
38 Jones, E. R. *et al.* Upper Palaeolithic genomes reveal deep roots of modern Eurasians. *Nat. Commun.* **6**, 8912, (2015).
39 Allentoft, M. E. *et al.* Population genomics of Bronze Age Eurasia. *Nature* **522**, 167-172, (2015).
40 Alexander, D. & Lange, K. Enhancements to the ADMIXTURE algorithm for individual ancestry estimation. *BMC Bioinformatics* **12**, 246, (2011).
41 Alexander, D. H., Novembre, J. & Lange, K. Fast model-based estimation of ancestry in unrelated individuals. *Genome Res.* **19**, 1655-1664, (2009).

42 Chang, C. *et al.* Second-generation PLINK: rising to the challenge of larger and richer datasets. *GigaScience* **4**, 7, (2015).
43 Purcell, S. *et al.* PLINK: a tool set for whole-genome association and population-based linkage analyses. *Am. J. Hum. Genet.* **81**, 559-575, (2007).
44 Skoglund, P., Storå, J., Götherström, A. & Jakobsson, M. Accurate sex identification of ancient human remains using DNA shotgun sequencing. *J. Archaeol. Sci.* **40**, 4477-4482, (2013).
45 Underhill, P. A. *et al.* The phylogenetic and geographic structure of Y-chromosome haplogroup R1a. *Eur. J. Hum. Genet.* **23**, 124–131, (2014).
46 Pamjav, H., Fehér, T., Németh, E. & Pádár, Z. Brief communication: New Y-chromosome binary markers improve phylogenetic resolution within haplogroup R1a1. *Am. J. Phys. Anthropol.* **149**, 611-615, (2012).
47 Hollard, C. *et al.* Strong genetic admixture in the Altai at the Middle Bronze Age revealed by uniparental and ancestry informative markers. *Forensic Science International: Genetics* **12**, 199-207, (2014).
48 Karafet, T. M. *et al.* New binary polymorphisms reshape and increase resolution of the human Y chromosomal haplogroup tree. *Genome Res.* **18**, 830-838, (2008).
49 Mathieson, I. *et al.* Eight thousand years of natural selection in Europe. *bioRxiv* 016477; doi: http://dx.doi.org/10.1101/016477, (2015).
50 Fu, Q. *et al.* Genome sequence of a 45,000-year-old modern human from western Siberia. *Nature* **514**, 445-449, (2014).
51 Seguin-Orlando, A. *et al.* Genomic structure in Europeans dating back at least 36,200 years. *Science* **346**, 1113-1118, (2014).
52 Raghavan, M. *et al.* Upper Palaeolithic Siberian genome reveals dual ancestry of Native Americans. *Nature* **505**, 87-91, (2014).
53 Kircher, M., Sawyer, S. & Meyer, M. Double indexing overcomes inaccuracies in multiplex sequencing on the Illumina platform. *Nucleic Acids Research* **40**, e3, doi:10.1093/nar/gkr771 (2012).
54 Kircher, M. in *Ancient DNA - Methods and Protocols Methods in Molecular Biology* (eds B Shapiro & M Hofreiter) (Springer, 2012).
55 Aronesty, E. *ea-utils: "Command-line tools for processing biological sequencing data"*, <http://code.google.com/p/ea-utils> (2011).
56 Li, H. & Durbin, R. Fast and accurate short read alignment with Burrows-Wheeler transform. *Bioinformatics* **25**, 1754-1760 (2009).
57 Li, H. *et al.* The Sequence Alignment/Map format and SAMtools. *Bioinformatics* **25**, 2078-2079 (2009).
58 Picard. *picard-tools*, <http://broadinstitute.github.io/picard/>
59 McKenna, A. *et al.* The Genome Analysis Toolkit: a MapReduce framework for analyzing next-generation DNA sequencing data. *Genome Res* **20**, 1297-1303 (2010).
60 TheInternationalHapMapConsortium. The International HapMap Project. *Nature* **426**, 789-796 (2003).
61 Cann, H. M. *et al.* A human genome diversity cell line panel. *Science* **296**, 261-262 (2002).
62 Jonsson, H., Ginolhac, A., Schubert, M., Johnson, P. L. & Orlando, L. mapDamage2.0: fast approximate Bayesian estimates of ancient DNA damage parameters. *Bioinformatics* **29**, 1682-1684 (2013).
63 Soejima, M., Tachida, H., Ishida, T., Sano, A. & Koda, Y. Evidence for recent positive selection at the human AIM1 locus in a European population. *Mol Biol Evol* **23**, 179-188 (2006).
64 Sabeti, P. C. *et al.* Genome-wide detection and characterization of positive selection in human populations. *Nature* **449**, 913-918 (2007).

65 Wilde, S. *et al.* Direct evidence for positive selection of skin, hair, and eye pigmentation in Europeans during the last 5,000 y. *Proc. Natl Acad. Sci.* **111**, 4832-4837 (2014).
66 Han, Y. *et al.* Evidence of positive selection on a class I ADH locus. *Am J Hum Genet* **80**, 441-456 (2007).
67 Li, H. *et al.* Diversification of the ADH1B gene during expansion of modern humans. *Annals of Human Genetics* **75**, 497-507 (2011).
68 Peng, Y. *et al.* The ADH1B Arg47His polymorphism in East Asian populations and expansion of rice domestication in history. *BMC Evol Biol* **10**, 15 (2010).
69 Clisson, I. *et al.* Genetic analysis of human remains from a double inhumation in a frozen kurgan in Kazakhstan (Berel' site, Early 3rd Century BC). *Int J Legal Med* **116**, 304-308 (2002).
70 Voevoda, M. I., Romaschenko, A. G., Sitnikova, V. V., Shulgina, E. O. & Kobsev, V. F. A Comparison of Mitochondrial DNA Polymorphism in Pazyryk and Modern Eurasian Populations. *Archaeology, Ethnology & Anthropology of Eurasia* **4**, 88-94 (2000).
71 Ricaut, F. X., Keyser-Tracqui, C., Bourgeois, J., Crubezy, E. & Ludes, B. Genetic analysis of a Scytho-Siberian skeleton and its implications for ancient Central Asian migrations. *Hum.Biol.* **76**, 109-125 (2004).
72 Ricaut, F. X., Keyser-Tracqui, C., Cammaert, L., Crubezy, E. & Ludes, B. Genetic analysis and ethnic affinities from two Scytho-Siberian skeletons. *Am.J.Phys.Anthropol.* **123**, 351-360 (2004).
73 González-Ruiz, M. *et al.* Tracing the origin of the east-west population admixture in the Altai region (Central Asia). *PloS one* **7**, e48904 (2012).
74 Pilipenko, A., Romaschenko, A., Molodin, V., Parzinger, H. & Kobzev, V. Mitochondrial DNA studies of the Pazyryk people (4th to 3rd centuries BC) from northwestern Mongolia. *Archaeological and Anthropological Sciences* **2**, 231-236, doi:10.1007/s12520-010-0042-z (2010).
75 Der Sarkissian, C. *Mitochondrial DNA in Ancient Human Populations of Europe*, University of Adelaide, (2011).
76 Nasidze, I. & Stoneking, M. Mitochondrial DNA variation and language replacements in the Caucasus. *Proc Biol Sci* **268**, 1197-1206 (2001).
77 Schönberg, A., Theunert, C., Li, M., Stoneking, M. & Nasidze, I. High-throughput sequencing of complete human mtDNA genomes from the Caucasus and West Asia: high diversity and demographic inferences. *Eur J Hum Genet* **19**, 988-994 (2011).
78 Quintana-Murci, L. *et al.* Where west meets east: the complex mtDNA landscape of the southwest and Central Asian corridor. *American Journal of Human Genetics* **74**, 827-845 (2004).
79 Terreros, M. C., Rowold, D. J., Mirabal, S. & Herrera, R. J. Mitochondrial DNA and Y-chromosomal stratification in Iran: relationship between Iran and the Arabian Peninsula. *J Hum Genet* **56**, 235-246 (2011).
80 Derenko, M. *et al.* Phylogeographic analysis of mitochondrial DNA in northern Asian populations. *Am J Hum Genet* **81**, 1025-1041 (2007).
81 Aime, C. *et al.* Human genetic data reveal contrasting demographic patterns between sedentary and nomadic populations that predate the emergence of farming. *Mol Biol Evol* **30**, 2629-2644 (2013).
82 Segurel, L. *et al.* Sex-specific genetic structure and social organization in Central Asia: insights from a multi-locus study. *PLoS Genet* **4**, e1000200 (2008).
83 Heyer, E. *et al.* Genetic diversity and the emergence of ethnic groups in Central Asia. *BMC Genet* **10**, 49 (2009).
84 Derenko, M. V. *et al.* Diversity of Mitochondrial DNA Lineages in South Siberia. *Annals of Human Genetics* **67**, 391-411 (2003).

85 Kong, Q. P. *et al.* Phylogeny of east Asian mitochondrial DNA lineages inferred from complete sequences. *American Journal of Human Genetics* **73**, 671-676 (2003).
86 Cheng, B. *et al.* Genetic imprint of the Mongol: signal from phylogeographic analysis of mitochondrial DNA. *J Hum Genet* **53**, 905-913 (2008).
87 Chaix, R., Austerlitz, F., Hegay, T., Quintana-Murci, L. & Heyer, E. Genetic traces of east-to-west human expansion waves in Eurasia. *Am J Phys Anthropol* **136**, 309-317 (2008).
88 Comas, D. *et al.* Trading genes along the silk road: mtDNA sequences and the origin of central Asian populations. American journal of human genetics 63, 1824-1838 (1998).
89 Yao, Y. G., Lu, X. M., Luo, H. R., Li, W. H. & Zhang, Y. P. Gene admixture in the silk road region of China: evidence from mtDNA and melanocortin 1 receptor polymorphism. *Genes Genet Syst* **75**, 173-178 (2000).
90 Malyarchuk, B., Derenko, M., Denisova, G. & Kravtsova, O. Mitogenomic diversity in Tatars from the Volga-Ural region of Russia. *Mol Biol Evol* **27**, 2220-2226 (2010).
91 Sajantila, A. *et al.* Genes and languages in Europe: an analysis of mitochondrial lineages. *Genome Research* **5**, 42-52 (1995).
92 Abecasis, G. R. *et al.* An integrated map of genetic variation from 1,092 human genomes. *Nature* **491,** 56-65 (2012).
